# First Total Synthesis of the Unnatural (+)-Talcarpine and (−)‑*N*_4_‑Methyl,*N*_4_‑21-*seco*talpinine

**DOI:** 10.1021/acsomega.5c13509

**Published:** 2026-04-29

**Authors:** Kamal Prasad Pandey, Md Toufiqur Rahman, Anna Benko, Gregory H. Imler, Jefferey R. Deschamps, James M. Cook

**Affiliations:** † Department of Chemistry and Biochemistry, 14751 University of Wisconsin—Milwaukee, Milwaukee, Wisconsin 53211, United States; ‡ 6856 RTI International, Center for Therapeutics, Toxicology, and Devices, Research Triangle Park, Durham, North Carolina 27713, United States; § 41487 Naval Research Laboratory, 4555 Overlook Avenue, Washington, D.C. 20375, United States

## Abstract

The indole scaffold is highly valued in medicinal chemistry due to its structural similarity to important molecules such as tryptamine and serotonin. We herein report the enantiospecific total synthesis studies of the unnatural C-19 methylated macroline-type indole alkaloids, (+)-talcarpine (**1**) and (−)-*N*_4_-methyl,*N*_4_-21-*seco*talpinine (**2**), via the asymmetric Pictet–Spengler reaction/Dieckmann cyclization process starting from commercially available and DNA encoded L-tryptophan (**3**). The desired stereochemistry was confirmed by using X-ray crystallography of the key intermediates and optical rotations.

## Graphical abstract

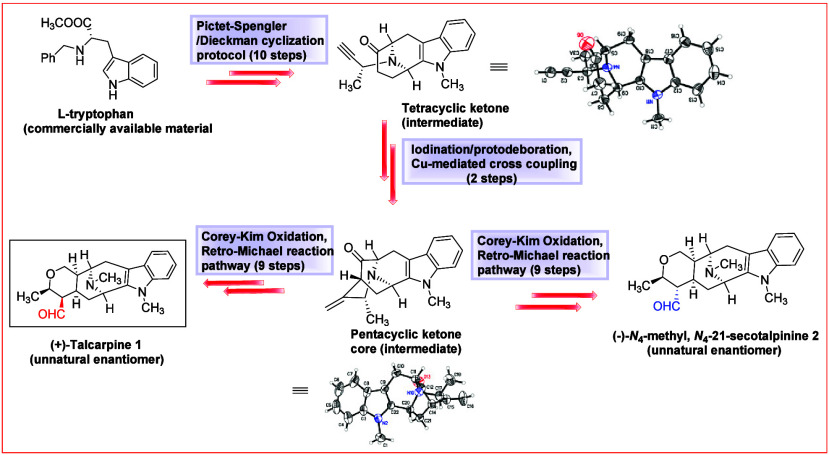

## Introduction

Nature has long served as a rich and enduring reservoir of biologically active compounds for medicinal purposes throughout history including cancer, malaria, leishmaniasis, cardiovascular diseases, and antiarrhythmic agents.^1–3^ Usually, molecules from nature are chiral secondary metabolites and purportedly used in the defense mechanism of plants or organisms.^3^ Many pharmaceuticals find their origin or inspiration from natural products.^4^ Besides their use from historical times, importantly, natural products bring new possibilities to modern medicinal chemistry as drugs “*beyond Lipinski’s rule of 5*”.^2,5^ Evidently, more than 60% of small-molecule pharmaceuticals approved by the FDA since the 1980s are either natural products, derived from natural products, or inspired by natural compounds.^1^ Many of these clinical applications correlate well with folk or traditional medicinal applications and continue to inspire new clinical applications. Despite the tremendous record of natural products in drug discovery and medicinal chemistry, natural-product-based drug discovery suffers from several hurdles, including the amount of active ingredient isolated in pure form from the natural source. Due to the paucity of material, structural complexity, synthetic challenges, and unsustainable supply of materials, further research and innovation are required in this vein.

Despite the challenges, it is anticipated that the advent of modern technologies such as automation, quantum computing, and cheminformatics will advance natural product-based drug discovery.^1,6^ Emerging technologies such as genome mining and engineering, advancements in analytical tools, artificial intelligence (AI), and progress in microbial culturing hold substantial promise for enhancing natural product-based drug discovery.^2,7^ Molecules of life are inherently chiral and predominantly produce chiral molecules under natural or physiological conditions. The natural enantiomer of any optically active natural product exists only in one particular enantiomer, and thus, it is not possible to isolate and screen the biological activity of the enantiomer.

From a rational standpoint, unnatural enantiomers could exhibit comparable or superior activity to natural enantiomers in certain instances. In particular, the enzymatic or metabolic stability of the unnatural enantiomer could be better compared to the natural enantiomer which might increase the potency and bioavailability of the unnatural enantiomer, resulting in a better drug. Furthermore, access to the unnatural enantiomer and its derivatives provides entry to novel chemical space and intellectual property, making this field attractive to pharmaceutical industries. Examples of successful unnatural enantiomers in medicinal chemistry include the blockbuster drug discovered by Professor Dennis C. Liotta, lamivudine (brand name: Epivir), an unnatural L-nucleoside-based medication and one of the WHO’s listed essential drugs. It has been very effective in saving HIV patients’ lives.^1,8^ Shiomi *et al.* reported the total synthesis of the unnatural (+)-quinine and 19-epi-quinine.^9^ Lindsley *et al.* reported the unnatural canthine alkaloids and their important activity for Akt inhibition.^10^ In a distinct investigation, the C′-20 urea enantiomeric derivates of vinblastine (a marketed drug) were found to be 10–200 times higher in anticancer potency against human tumor cell lines, as compared to the natural vinblastine urea analogues.^1,11^

The C-19 methyl-substituted sarpagine/macroline/ajmaline alkaloids are an important subgroup of indole alkaloids, first detailed by Lounasma.^6,12^ Over 70 of these monomeric alkaloids have been isolated, primarily from Alstonia species.^1,13,14^ These bioactive compounds are distinguished by at least one monomeric unit with a C-19 methyl group, which likely comes from precursors similar to their demethylated counterparts. Their synthesis is feasible using common intermediates, as sarpagine, macroline, and ajmaline alkaloids share a common azabicyclo[3.3.1]­nonane core **4**.^1^ To date, a number of alkaloids with important biological activity have been isolated and synthesized from this group by using this common core. Hashimoto’s team (2022) developed an enantioselective method for constructing an *N*-bridged [3.3.1] ring system, aiding synthetic efforts for these alkaloids via biocatalytic asymmetric desymmetrization and amide insertion.^15^ Recently, Zhang’s team (2023) synthesized a structurally related tetracyclic sarpagine core starting from L-tryptophan via a Bischler–Napieralski reaction followed by a homo-Mannich sequence in four synthetic steps.^16^ In addition, the team constructed a sarpagine scaffold from a bridged skeleton through key transformations, including a tandem sequential oxidative cyclopropanol ring-opening cyclization and ketone α-allenylation.^17^

A sarpagine-related indole alkaloid, (−)-talpinine **5** (Figure 1), was isolated from the stem bark and root bark of *Pleiocarpa talbotii* and exhibited anticancer activity against KB/VJ300 cells with IC_50_ values ranging between 14 and 22 μM in the presence of 0.1 μM added vincristine.^19,20^ Structurally related *O*-acetyltalpinine **6**, also a sarpagine-type indole alkaloid, was isolated from the stem bark and leaves of *Alstonia angustifolia*.^20^ This indole alkaloid also exhibited anticancer activity against the KB/VJ300 cells.^20^ A macroline-type indole alkaloid, (+) *N*_4_-methyl,*N*_4_-21-*seco*talpinine **7** isolated from the stem bark of *A. macrophylla* and *A. angustifolia* exhibited antileishmanial activity against the promastigotes of *Leishmania mexicana* with an IC_50_ value of 57.8 μM.^21,22^ Alstonerinal **8** (macroline-type) was isolated from the stem bark and leaves of *A. macrophylla* and the *A. angustifolia* plant.^18,23,24^ This alkaloid also exhibited strong activity against colon cancer cell lines (HT-29 cells with an ED_50_ value of 8.6 μM). Moreover, it was also antileishmanial against the promastigotes of *L. mexicana* (IC_50_ 145.4 μM).^18^ (−)-Talcarpine **9** is a macroline-type indole alkaloid. It was isolated from the stem bark and leaves of various plant species: *Pleiocarpa talbotii Wernham*, *A. macrophylla*, and *A. angustifolia*.^18,25–28^ This monomeric indole alkaloid exhibited weak antimalarial activity against *Plasmodium falciparum* (IC_50_ 40.3 ± 2.9 μM).^28^ A sarpagine-type C-19 methyl substituted indole alkaloid, isolated from the stem bark and leaves of *A. angustifolia*, (−)-*N*_4_-methyltalpinine **10** exhibited potent anticancer activity against the NF-κB cell line with an ED_50_ value of 1.2 μM.^22,29^

**Figure 1:**
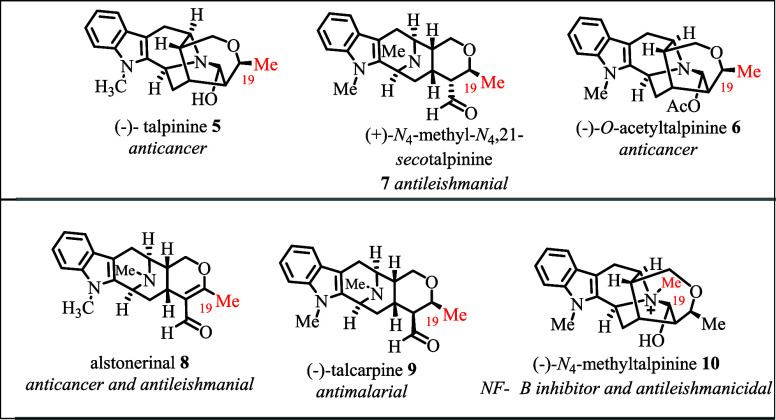
Natural C-19 methyl-substituted sarpagine/macroline-related alkaloids (representative examples) with their biological activity.^1,6,18,19^

Practical and large-scale access to the key intermediate is a prerequisite for a successful medicinal chemistry campaign that deals with complex alkaloids. In addition, a general strategy would ensure access to most if not all of these structurally related alkaloids. This is also applicable to the efficient synthesis of the enantiomers. The Milwaukee team has created an effective and feasible approach for synthesizing various bioactive indole alkaloids that are related to sarpagine, macroline, and ajmaline.^6,30,31^ Furthermore, the unified general approach was applied to complete the total synthesis of over a dozen indole alkaloids belonging to this subfamily.^6,30,32–36^ The ambidextrous Pictet–Spengler/Dieckmann cyclization method, developed by Rahman *et al.*, provides a highly efficient and stereospecific approach for synthesizing both natural and synthetic enantiomers of various alkaloids either from d- or L-tryptophan as the starting materials (Scheme 1).^1,12^ The total synthesis of several alkaloids (talpinine **5**, *O*-acetyl talpinine **6**, *N*_4_-methyltalpinine **10**) was reported employing this method.^3^

**Scheme 1:**
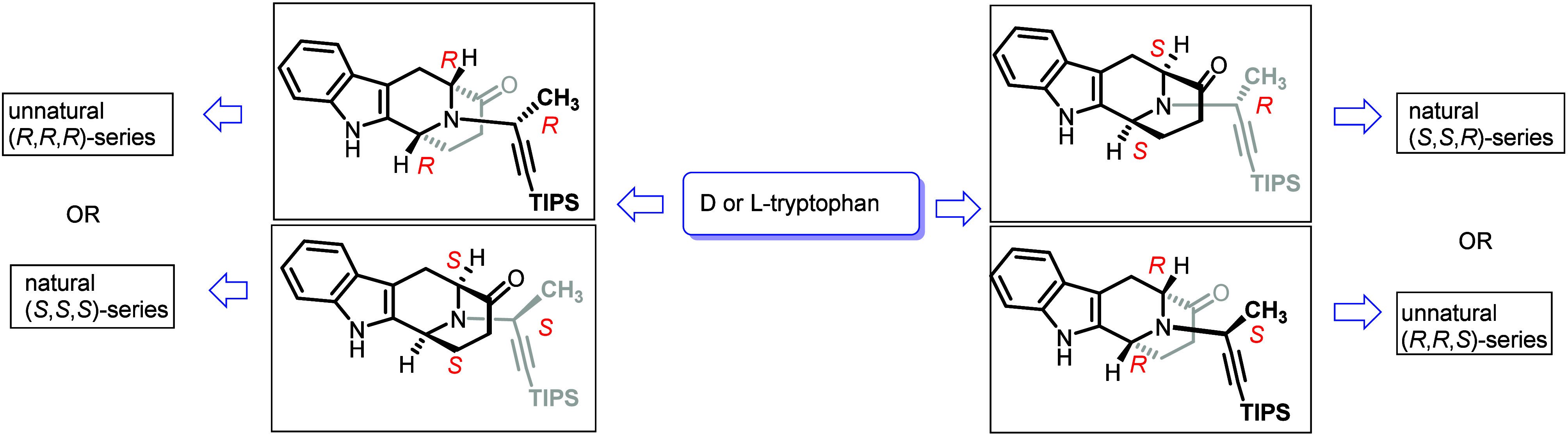
Access to Both Natural and Unnatural Enantiomers of C-19 Methyl-Substituted Sarpagine/Macroline Indole Alkaloids from Either d- or l-Tryptophan Methyl Ester^1,12,18^

By employing a unified general strategy, the enantiospecific total synthesis of the first example of sarpagine-type alkaloids that belong to this C-19 methylated group was reported by Edwankar *et al.* ^32^ Employing the same strategy, the formal synthesis of (−)-talcarpine **9** which belongs to the same group was reported. Afterward, Rahman *et al.* reported a robust and less expensive copper-mediated enolate-driven cross-coupling process to access the core pentacyclic ketone **11**.^33^ We disclose the total synthesis of two unnatural indole alkaloids: (+)-talcarpine **1** and (−)-*N*_4_-methyl,*N*_4_-21-*seco*talpinine **2** via the efficient asymmetric Pictet–Spengler reaction/Dieckmann cyclization process and copper-mediated enolate-driven cross coupling process starting from commercially available L-tryptophan **3 (** Figure 2 **).**

**Figure 2:**
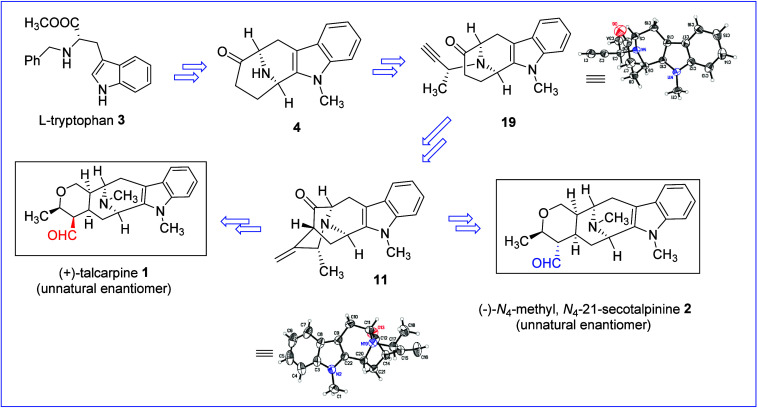
Schematic representation of the enantiospecific synthesis of the unnatural enantiomers: (+)-talcarpine **1** and (−)-*N*_4_-methyl,*N*_4_-21-*seco*talpinine **2**.

## Results and Discussion

The L-tryptophan methyl ester **12**, which resulted from commercially available L-tryptophan **3** via a Fischer-esterification reaction (420 g scale, 99% yield), was converted into the *N*_b_-benzyl L-tryptophan methyl ester **13** on 320 g scale in 84% yield by the employment of benzaldehyde and the reducing agent (sodium borohydride) at low temperature, as shown in Scheme 2. It is crucial to add the reducing agent at a lower temperature and portionwise to circumvent the possibility of the racemization of the imine intermediate at this step.^37^

**Scheme 2:**
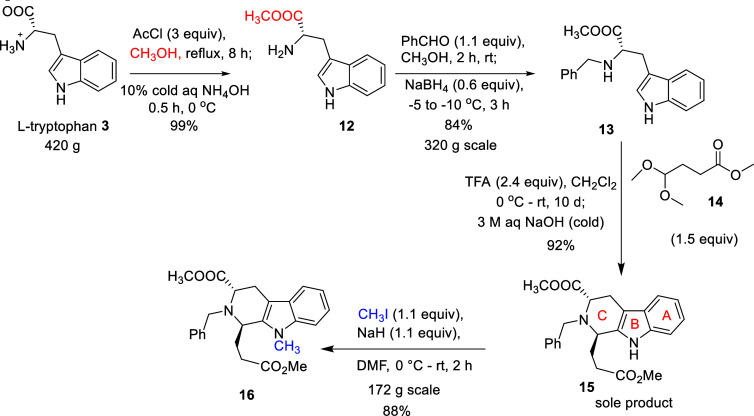
Large-Scale Synthesis of l-Tryptophan Methyl Ester (**12**) and *N*_b_-Benzyl l-Tryptophan Methyl Ester (**13**)

The 4,4-dimthoxybutyrate **14**, a precursor needed for Pictet–Spengler reaction, was prepared on large scale (610 g) via a Michael addition reaction and a Nef reaction following the published literature procedure.^36^

For decades, the asymmetric Pictet–Spengler reaction has been highly studied and executed on a large scale in Milwaukee, which resulted in the successful total synthesis of many natural products.^37–40^ The employment of *N*_a_-H,*N*_b_-benzyl L-tryptophan **13** and the methyl 4,4-dimethoxy butyrate **14** in the presence of a strong acid (trifluoroacetic acid) provided the *trans*-diester **15** as the sole product in 92% yield after stirring for 10 days. The rate of this reaction can also be improved by heating the reaction mixture in chloroform for 10 h.

The employment of the d-tryptophan-based starting material and the use of the weaker acid (acetic acid) via a *cis*-specific Pictet–Spengler reaction/Dieckman cyclization protocol also allows the desired intermediate to proceed to the unnatural enantiomers.^12^ However, the L-tryptophan **3** starting material with a *trans*-specific Pictet–Spengler reaction was carried out because of the much lower cost of commercially available natural L-tryptophan, compared to the d-tryptophan. The *trans*-diester **15** which resulted was immediately converted into the *N*_a_-Me, *trans-*diester **16** (Scheme 2) in 88% yield. Both intermediates, *N*_a_-H *trans-*diester **15** and *N*_a_-Me *trans-*diester **16**, were purified by crystallization (see Experimental Section for details).

After installing the C-ring (*N*_a_-H, *N*_b_-benzyl, *trans*-diester **15**) attached to the indole via a Pictet–Spengler reaction and the methylation of the subsequent product, the synthesis progressed toward building on the fourth ring (D) containing the *N*_a_-Me, *N*_b_-benzyl tetracyclic ketone **17** on large scale. The *N*_a_-Me, *N*_b_-benzyl, *trans*-diester **16** (202 g) was converted into the β-keto ester **18** via a Dieckmann cyclization under refluxing conditions, which resulted in an 87% yield of the enol on a large (196 g) scale.^36^ The two chiral centers in the β-keto ester **18** formed in this step contained the desired *R*, *R* stereochemistry, which was later confirmed by X-ray crystallography of intermediate **19**. The ester intermediate **18**, which resulted from the Dieckmann cyclization reaction, was used directly in the next step without further purification and characterization.

The methyl ester of the crude product (β-keto ester **18**) was hydrolyzed (in an acidic medium) into the corresponding carboxylic acid (structure not shown) on a 196 g scale (Scheme 3). This was followed by decarboxylation under refluxing conditions in the same reaction vessel. To reduce the amount of base required to basify salt **20**, most of the solvents (glacial acetic acid and conc. hydrochloric acid) were removed under reduced pressure prior to basification of the crude mixture. These two chemical transformations resulted in the desired *N*_a_-Me, *N*_b_-benzyl, tetracyclic ketone **17** in 88% yield (145 g), which was then purified by crystallization.

**Scheme 3:**
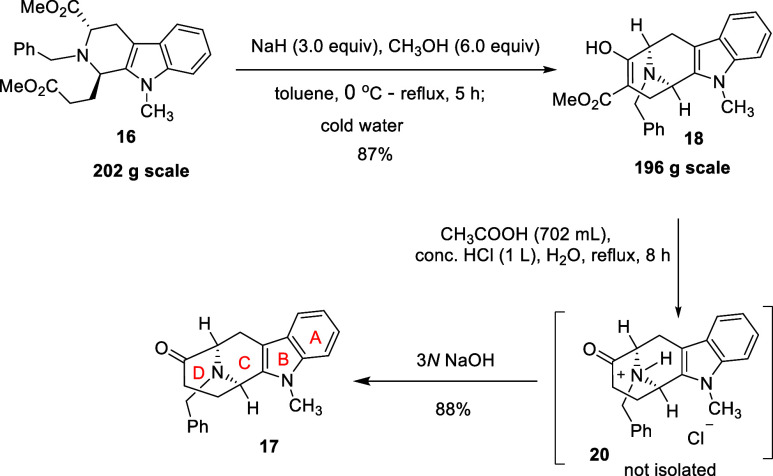
Large-Scale Synthesis of the *N*_a_-Methyl *N*_b_-Benzyl Tetracyclic Ketone **17**

To conduct catalytic debenzylation reaction on the ketone **17**, the ketone starting material **17** on a 43.3 g scale was first converted into the quaternary ammonium salt (structure not shown) using acetyl chloride in ethanol. This process involves the *in situ* generation of dry HCl gas required to form the salt. The salt, thus formed, was then subjected to the debenzylation process using a catalytic amount of Pd/C (5 mol %) and H_2_ gas at room temperature.^32^ This resulted in crucial azabicyclo[3.3.1]­nonane core **4** with the desired A, B, C, and D ring structures. The ketone **4** which resulted (86% yield) after filtration through Celite and an alkaline workup was pure by spectroscopic analysis (^1^H NMR and ^13^C NMR, see the ). Thus, no further purification of the product was required using this modified method, which was an improvement over the previously reported procedure. As reported in the literature, the starting material can also be converted into its hydrochloride salt by adding ethanol (saturated with anhydrous HCl gas); however, the purification steps required either crystallization or column chromatography by this method.^3,12,30,36^ The tetracyclic core **4**, a key intermediate, can now be employed in the total synthesis of unnatural enantiomers of C-19 methyl substituted sarpagine/macroline/ajmaline-type indole alkaloids.^1^ Also, tetracyclic ketone **4** was an optically active enantiomer of the core structure prepared from d-tryptophan, as reported in published articles.^30,34^ Moreover, the synthesis of this core structure **4** can also be executed via the ambidextrous Pictet–Spengler reaction either from d- or L-tryptophan.^12^ The large-scale (250 g scale) preparation of the (*S*)-4-*tri*isopropylsilyl-3-butyn-2-yl tosylate **21** was carried out by following the procedure published by Edwanker *et al.* for the synthesis of its natural enantiomer (*R*)-4-*tri*isopropylsilyl-3-butyn-2-yl tosylate (structure not shown).^30,35,36,41^

After successful synthesis of (*S*)-TIPS tosylate **21**, and (*R*,*R*)-tetracyclic ketone **4** (core structure with A, B, C and D ring) on a large scale, the research direction turned toward the preparation of the E-ring (the fifth ring), which contained the pentacyclic ketone **11**. The (*R*,*R*)-tetracyclic ketone **4** was alkylated using (*S*)-TIPS tosylate **21** and potassium carbonate under refluxing conditions, with stirring (Scheme 4) to yield 91% of *N*_b_-alkylated (*R*,*R*)-tetracyclic ketone **22**, which was purified by flash chromatography. The *N*_b_-alkylated (*R*,*R*)-tetracyclic ketone **22** underwent deprotection of the *tri*-isopropyl silane (TIPS) group (73.1 g scale) as shown in Scheme 4 in the presence of fluoride ion (TBAF·H_2_O) at 0 °C to afford ketone **19** in 98% yield. The desired (*R*,*R*,*R*) stereochemistry required in acetylene **19** was confirmed by X-ray crystallography. The X-ray crystallographic data were very encouraging because the synthetic process that we carried out was efficient to control absolute stereochemistry and atomic configuration as desired. Moreover, the value of the optical rotation (see Figure 3 and for X-ray crystallographic data) of the ketone **19** was in complete agreement with the reported value in the literature for its natural enantiomer.^30,34,36^ Although Rahman *et al.* introduced a shorter route for synthesizing tetracyclic ketone intermediate **19**, the traditional Pictet–Spengler–Dieckmann protocol remains a more reliable and practical option. This approach bypasses the need for purification, as crystals can be directly obtained, eliminating the necessity of labor-intensive large-scale chromatography.^12^ Additionally, the Rahman *et al.* method was not adapted or optimized for the larger-scale synthesis outlined in this report. Notably, much of the work described here was completed before the ambidextrous method was published.

**Scheme 4:**
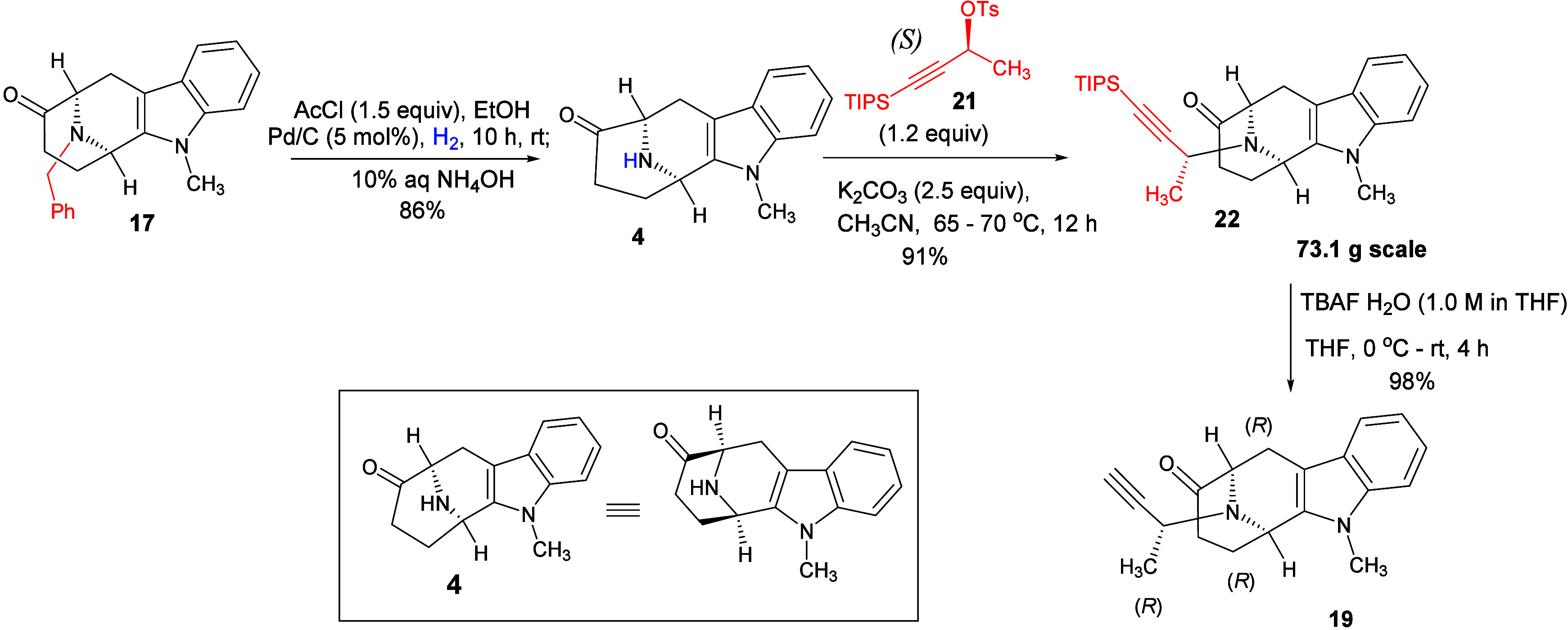
Synthesis of (*R*,*R*,*R*)-Acetylenic Tetracyclic Ketone (**19**) via Alkylation of Tetracyclic Intermediate **4** and the Removal of the TIPS Functional Group

**Figure 3:**
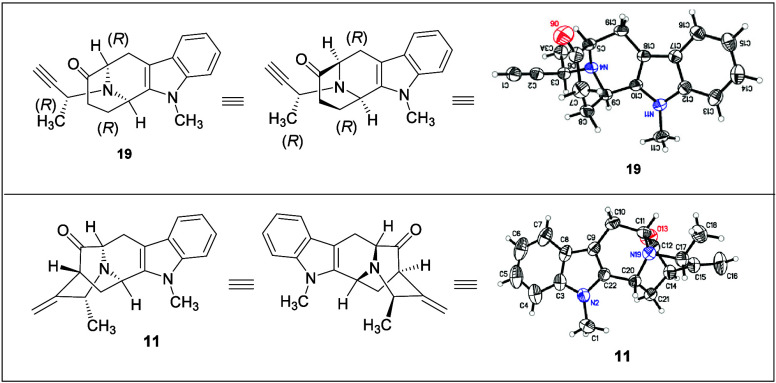
Molecular structure and ORTEP view of **19** and **11**.

There were several different procedures reported to prepare vinyl iodides, including the use of a regioselective B-iodo-9-BBN [BBN = borabicyclo (3.3.1) nonane] iodoboration/protodeboration,^36,43^ as well as the Mo-catalyzed hydrostannation or the Pd-catalyzed stannation.^30^ However, none of these techniques worked well for the synthesis of the required vinyl iodide [enantiomer of **23**], as discussed by Edwankar *et al.* ^30,36^ The bicyclohexyl iodoborane [IB­(Cy)_2_] was reported as a highly efficient regioselective reagent for the iodoboration for the alkenyl (*S,S,S*)-tetracyclic ketone (not shown).^30^ Consequently, the desired vinyl iodide **23** was synthesized following the published work of Edwankar *et al.* using regioselective IB­(Cy)_2_.^30^ The iodoboration of ethynyl ketone **19** was carried out by the employment of 2.5 equiv of IB­(Cy)_2_, whereas protodeboronation was carried out by using 11 equiv of glacial acetic acid, which was added with stirring, at 0 °C. This was followed by the addition of 3 M NaOH and hydrogen peroxide to oxidize boron byproducts. This process was followed by the addition of 5% aqueous sodium bisulfite to remove excess iodine (Scheme 5). The vinyl iodide **23** resulted in 80% yield after flash chromatography (please see Experimental Secion for details).

**Scheme 5:**
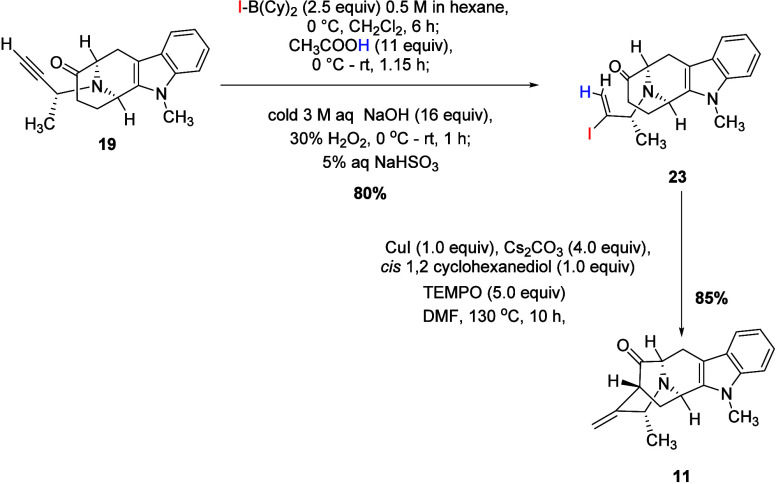
Synthesis of Vinyl Iodide (**23**) and Pentacyclic Ketone (**11**)

The vinyl iodide **23** which resulted was converted into the pentacyclic ketone **11** via a Cu-mediated cross-coupling reaction by following the procedure reported by Rahman *et al.* ^33^ This procedure involved the employment of 1 equiv of copper iodide, *cis* 1, 2-cyclcohexane diol (ligand), 4 equiv of cesium carbonate, and 5 equiv of TEMPO (as a free radical scavenger).^33^ The Cu-mediated cross-coupling process to prepare the pentacyclic ketone (enantiomer of **11**) reported herein was an improved method with an increased yield without the undesired 7-membered by product (structure not shown), as compared to the use of a palladium catalyst.^30,33^

The key intermediate, pentacyclic ketone **11**, was afforded in an 85% yield after purification by flash column chromatography. A small amount of ketone **11** was crystallized using ethyl acetate to obtain X-ray crystallographic data. Examination of the ORTEP view here (Figure 3) confirmed the desired stereochemistry and atomic configuration. Moreover, the spectroscopic data (^1^H NMR, ^13^C NMR), LCMS, *R*_f_ value, and the optical rotation (opposite direction) of **11** were in complete agreement with that reported for its natural enantiomer.^33^

After synthesis of the pentacyclic ketone **11**, it was then subjected to a one carbon homologation process via a Wittig reaction, according to the published procedure.^30,32,33,36^ The ylide (not shown) was prepared by stirring the Wittig reagent [(methoxymethyl)­triphenylphosphonium chloride] with potassium *tert*-butoxide in dry benzene at room temperature. The yellow color that formed in the reaction mixture changed to a dark red color after the mixture was stirred for 30 min, indicating the formation of the ylide, after which the starting material was added to the reaction solution at 0 °C (Scheme 6). The pentacyclic methyl enol ether (intermediate) **24** which resulted was not isolated but was subjected to acid hydrolysis to afford the pentacyclic aldehyde **25** (86%). The phosphorus byproducts present in the reaction mixture were removed by washing with hexanes 6–7 times, shaking vigorously, and decanting the upper layer (hexane layer). The pentacyclic aldehyde **25** was found to have the α stereochemistry at C because the *R*_f_ value matched precisely with the authentic sample of its enantiomer (structure not shown), in which the α position had been confirmed by X-ray crystallography.^29^ Pentacyclic aldehyde **25**, without isolation, was then reduced to pentacyclic alcohol **26** (92% yield) with sodium borohydride (1.5 equiv) as the reducing agent. Skipping the isolation step of aldehyde **25** resulted in an increase of the yield of the reaction by 5% because of the possible loss of α aldehyde **25** during silica gel chromatography. The pentacyclic alcohol **26** (white-colored waxy solid), which formed after the reduction of aldehyde **25** in the next step, was purified by flash column chromatography.

**Scheme 6:**
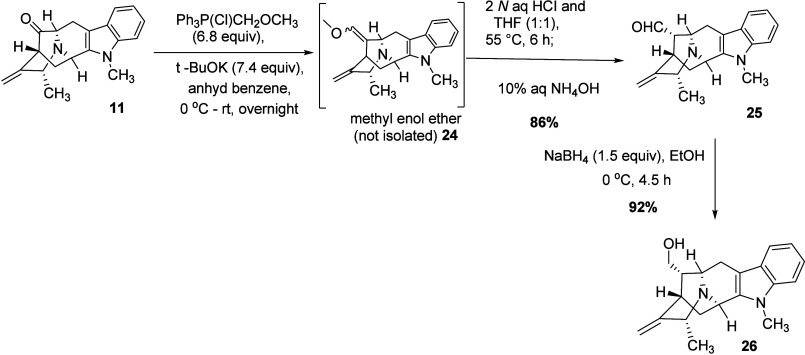
Synthesis of Pentacyclic Alcohol **26**

The pentacyclic alcohol **26** was then protected by a TIPS protecting group using the hindered base (2,6-lutidine) and TIPS-triflate (Scheme 7). This step resulted in silyl ether **27** in excellent yield (91%). The silyl ether **27** which resulted was then subjected to hydroboration (DMS.BH_3_) and a Kabalka oxidation on 3.5 g scale by following the published procedure.^30,34,36^ The application of hydrogen peroxide as an oxidizing agent was not utilized because of the susceptibility to the formation of an *N*_b_-oxide (structure not shown), which would be difficult to remove, as discussed by Edwankar *et al.* ^30^ Thus, sodium perborate was employed as an oxidizing reagent. The crude mixture which resulted was heated to reflux with 5 equiv of sodium carbonate in methanol to cleave the *N*_b_-boronate bond.^30^ This hydroboration and Kabalka oxidation step resulted in pentacyclic alcohol **28** in 61% yield. The primary alcohol **28** was purified by flash column chromatography and obtained as a white waxy solid.

**Scheme 7:**
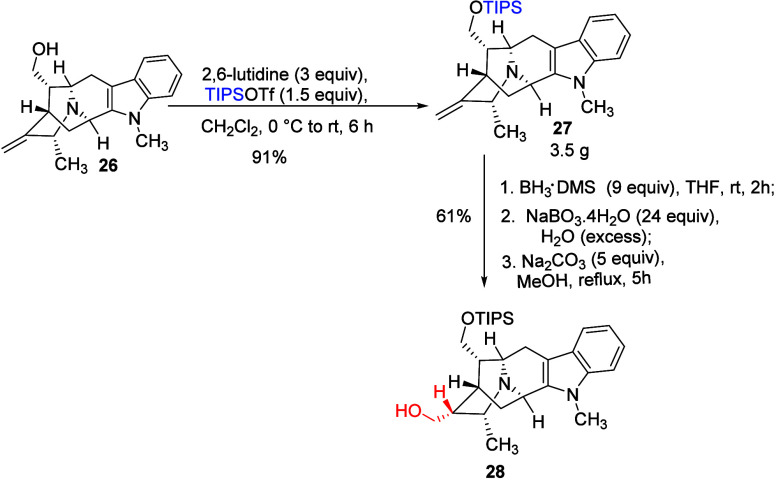
Synthesis of Silyl Ether (**27**) and the Synthesis of the Desired Primary Alcohol (**28**)

An efficient and modified Corey-Kim oxidation was reported by the Milwaukee group earlier.^30,36^ The use of other oxidizing agents such as Dess-Martin periodinane or hydrogen peroxide were avoided because of the susceptibility of the *N*_4_ nitrogen atom to form the corresponding *N*-oxide adduct, as discussed.^30^ The primary alcohol **28** was oxidized following the procedure as reported to obtain the mixture of aldehydes (α and β) as shown in Scheme 8.^30^ In this oxidation reaction, the formation of a white precipitate was observed immediately after adding DMS dropwise to the solution of NCS in dichloromethane, indicating the formation of the reactive sulfur cationic species. This was followed by the addition of alcohol **28** at −78 °C. The mixture of aldehydes (α and β) were converted into the more stable α aldehyde **29** (thermodynamic product) as the sole epimer in 70% yield using methanol and triethylamine. The aldehyde **29** was purified by flash column chromatography as an oily material. The formation of an aldehyde was confirmed by LCMS, HRMS, and spectral analysis (^1^H NMR, ^13^C NMR, DEPT-135 ^13^C spectra; please see Experimental Section for details).

**Scheme 8:**
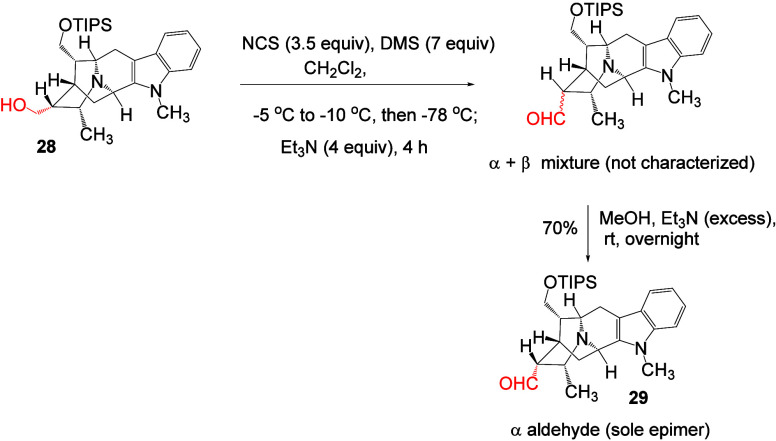
Synthesis of α-Aldehyde (**29**)

After the synthesis of the α-aldehyde **29**, it was subjected to quaternarization of the *N*_b_-nitrogen atom using an excess of iodomethane in methanol. The formation of the quaternary salt was easily confirmed by LCMS, HRMS, *R*_*f*_ (as compared with the authentic sample: enantiomer of **30** by Rahman *et al.*).^34^ In addition, the analysis of HRMS and NMR (crude ^1^H NMR and ^13^C NMR) spectroscopic data matched those of the natural enantiomer. The quaternary ammonium salt **30** which resulted was then employed in the *retro*-Michael reaction by a modification of a process by LeQuesne *et al.* At this step, when a solid NaHMDS base was used, the reaction did not work (Table 1). However, when NaHMDS (1.0 M) in THF was employed, the reaction proceeded, albeit in low yield (41% crude yield), as compared to that reported in the literature for its enantiomer.^34^ The α,β-unsaturated aldehyde **31** was characterized by LCMS and HRMS analysis; however, it was not isolated to avoid the loss of material during purification. Moreover, the *R*_*f*_ value (0.27, silica gel, 5% MeOH in CH_2_Cl_2_) when compared to the authentic sample of its enantiomer also matched.^34^ The crude aldehyde **31** was then used in the final step. The TIPS protecting group in the crude α,β-unsaturated aldehyde **31** was removed by refluxing in the mixture of aqueous HCl and THF, and the alcohol which resulted *in situ* underwent an *oxa*-Michael reaction (Scheme 9). After refluxing the reaction mixture for 6 h, the analysis by TLC indicated the formation of two components: one intense component (higher *R*_*f*_) and another fainter (lower *R*_*f*_) component. The *R*_*f*_ values of both components were identical, as expected for those of the authentic samples of the corresponding natural enantiomers [(−)-talcarpine **1** and (+) *N*_4_-methyl,*N*_4_-21-*seco*talpinine **2**].^34,41^ The crude material was purified by using flash column chromatography. The formation of the unnatural enantiomer (+)-talcarpine **1** was confirmed by NMR, LCMS, HRMS, and comparison of the *R*_***f***_ value (with the natural enantiomer), as shown in the . The lower faint indole component after isolation by flash chromatography was analyzed by LCMS, HRMS, and *R*_*f*_ value and compared to the natural (+)*N*_4_-methyl,*N*_4_-21-*seco*talpinine **2**. These analytical data were in complete agreement with those of the natural enantiomer. Thus, the minor product obtained in this reaction was the desired unnatural (−)*N*_4_-methyl,*N*_4_-21-*seco*talpinine **2** indole alkaloid. Unfortunately, this material was not present in a high enough amount for further characterization.

**Table 1:** Optimization of the *Retro*-Michael Reaction

Scale	Reagent	Equivalent	Time	% Yield
40 mg	NaHMDS (solid)	1.5	10 h	No reaction
55 mg	NaHMDS (solid)	2.0	12 h	No reaction
45 mg	NaHMDS (solid)	2.5	20 h	No desired product seen by TLC and LCMS analysis. Only baseline impurities seen.
15 mg	NaHMDS (1 M solution in THF)	3.0	16 h	41%

**Scheme 9:**
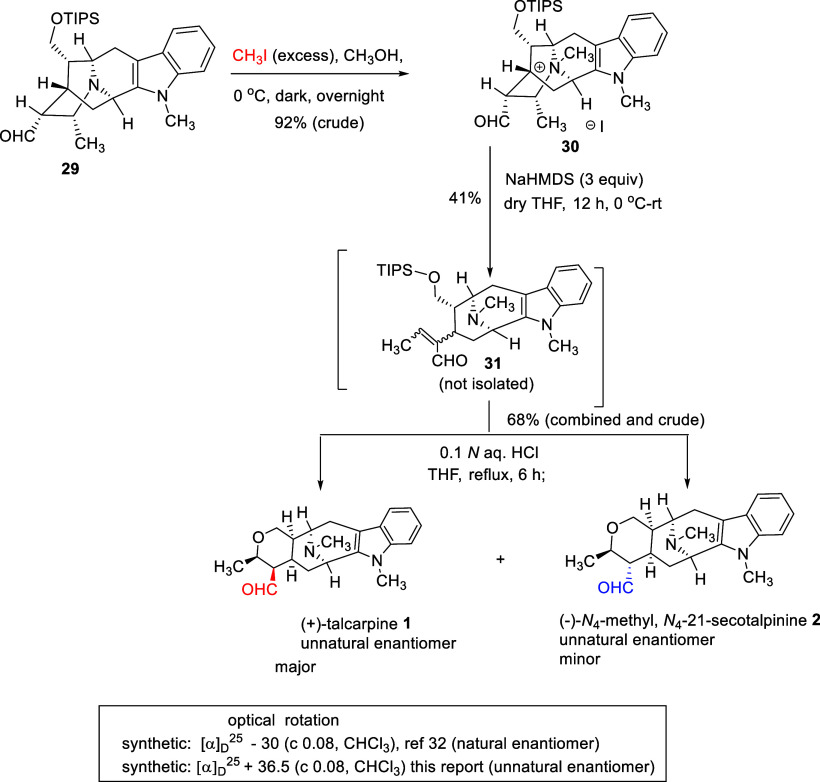
Synthesis of (+)-Talcarpine **1**, and (−)*N*_4_-Methyl,*N*_4_-21-*seco*talpinine **2** via Quaternization of the α-Aldehyde (**29**), *retro*-Michael Reaction, Acidic Hydrolysis, and then Intramolecular Cyclization (*In Situ*)

## Conclusion

Utilizing the Pictet–Spengler reaction/Dieckmann protocol, the enantiospecific total synthesis of the unnatural enantiomer (+)-talcarpine **1** was accomplished for the first time. The robust synthesis was initiated from cost-effective, naturally occurring, and DNA-encoded L-tryptophan **3**. The desired stereochemical control was excellently achieved, as evidenced by X-ray analysis of key intermediates (tetracyclic ketone **19** and pentacyclic ketone **11**), along with the determination of optical rotation values and spectral data (^1^H NMR and ^13^C NMR), which were subsequently compared to those of the natural enantiomers. The formation of another unnatural indole alkaloid, (−)-*N*_4_-methyl,*N*_4_-21-*seco*talpinine **2** in the final ring closing step was confirmed by LCMS, HRMS, and the comparison of *R*_*f*_ value with the natural enantiomer. This alkaloid **2** was not present in a high enough amount for further characterization. Large-scale synthesis of early intermediates was implemented, and crystallization techniques were applied to purify the reaction products at most steps whenever feasible. The Pictet–Spengler/Dieckmann protocol afforded excellent yields in most of the steps, except in the *retro*-Michael ring opening reaction. This provides the proof of concept that by employing both the Pictet–Spengler reaction/Dieckmann reaction and ambidextrous Pictet–Spengler method, the natural and unnatural enantiomers of the C-19 methyl substituted sarpagine/macroline/ajmaline type indole alkaloids can be synthesized. A scale up of the final 3 steps will be required to provide the necessary material for biological screening.

## Experimental Section

### General Experimental Conditions

Unless specified otherwise, oven-dried round-bottom flasks or screw-cap test tubes were utilized in all reactions. Chemicals were purified through standard methods and procured from commercially available vendors. Flash column chromatography employing silica gel (230–400 mesh, Dynamic Adsorbents), neutral alumina, or basic alumina was employed for the purification of compounds. Melting points were determined using a Stuart melting point SMP3 apparatus from Barloworld Scientific US Ltd. The ^1^H and ^13^C NMR spectra were acquired on a Bruker Spectrospin 500 or 300 MHz instruments in CDCl_3_ or in methanol-d^4^. Isopropyl alcohol was purified and dried by distillation over calcium sulfate and drying with molecular sieves (3 Å). Chemical shifts are reported in δ (ppm), and multiplicities are denoted as follows: singlet (s), broad signal (br), doublet (d), triplet (t), quartet (q), dd (doublet of doublets), dt (doublet of triplets), td (triplet of doublets), and multiplet (m). High-resolution mass spectrometry (HRMS) was conducted on an LCMS-IT-TOF instrument at the Milwaukee Institute for Drug Discovery (MIDD) in the Shimadzu Laboratory for Advanced and Applied Analytical Chemistry. Optical rotations were measured by using a JASCO Model DIP-370 digital polarimeter. **Benzene was distilled over CaH**_**2**_ **and dried using molecular sieves (3 Å), with careful handling in a hood.**

### Synthesis of L-Tryptophan Methyl Ester (12)

To a three-neck round-bottom flask (5 L) were added L-tryptophan **3** (420 g, 2.05 mol) and 2000 mL of anhydrous methanol. The temperature of the flask was lowered to 0 °C (ice bath). To the reaction mixture, 3 equiv (6.16 mol, 440 mL) of acetyl chloride was added dropwise and the reaction mixture was allowed to warm to rt and then heated to reflux for 8 h. The completion of the reaction was confirmed on analysis by TLC (*R*_*f*_ 0.4 in 5% methanol/dichloromethane + a few drops of 14% aq ammonium hydroxide solution). The free base **12** was prepared by basifying the reaction mixture (pH 10) by dropwise addition of 10% aq NH_4_OH solution at 0 °C. The aq layer was extracted with CH_2_Cl_2_ (3 × 100 mL). The combined organic layers were washed with brine (100 mL) and dried (K_2_CO_3_). The removal of the organic solvents under reduced pressure furnished a pure light brown colored crystalline product: [α]_D_^25^ + 35.4 (c. 1.18, MeOH); Lit. value for its enantiomer: [α]_D_^27^ - 37.2 (c 1.0, MeOH);^36^ ^**1**^ **H NMR** (500 MHz, CDCl_3_) δ 8.36 (s, 1H), 7.64 (d, *J* = 7.9 Hz, 1H), 7.37 (d, *J* = 8.1 Hz, 1H), 7.22 (t, *J* = 7.4 Hz, 1H), 7.15 (t, *J* = 7.4 Hz, 1H), 7.06 (d, *J* = 1.4 Hz, 1H), 3.86 (dd, *J* = 7.6, 4.8 Hz, 1H), 3.74 (s, 3H), 3.31 (dd, *J* = 14.4, 4.7 Hz, 1H), 3.15–3.04 (m, 1H), 1.70 (s, 2H); ^**13**^ **C NMR** (126 MHz, CDCl_3_) δ 175.8, 136.3, 127.5, 123.0, 122.2, 119.5, 118.7, 111.0, 54.9, 52.1, 30.7. The spectroscopic data (^1^H and ^13^C NMR) and optical rotation value for this compound **12** were in excellent agreement with that reported in the literature for its enantiomer.^30,32,37^

### Methyl (1*R*,3*S*)-2-Benzyl-1-(3-methoxy-3-oxopropyl)-2,3,4,9-tetrahydro-1*H*-pyrido­[3,4-*b*]­indole-3-carboxylate (15)

To a 3-neck round-bottom flask (5 L), which contained a solution of *N*_b_-benzyl-L-tryptophan methyl ester **13** (150 g, 0.48 mol) in CH_2_C1_2_ (1400 mL), methyl 4,4-dimethoxybutyrate acetal **14** (112.5 mL, 0.7 mol), and TFA (82.2 mL, 2.4 equiv) were added at 0 °C. The reaction mixture was stirred at rt for 10 d and then cooled to 0 °C (ice bath), and brought to pH 8 with a dropwise addition of cold aq solution of NaOH (3 *N*). The layers were separated, and the aq layer was extracted with CH_2_C1_2_ (3 × 900 mL). The combined organic layer was washed with brine (2 × 400 mL) and dried (K_2_CO_3_). The volume of the solution was reduced to 150 mL under reduced pressure, after which EtOAc (50 mL) and hexanes (100 mL) were added to the solution. The solution was cooled to −20 °C. The *trans* diester **15** precipitated (EtOAc/hexane, 1:5) out as white crystals, and the mother liquor was concentrated. The mother liquor which resulted was purified by flash chromatography (silica gel, EtOAc/hexane, 1:4) to provide an additional (5 g) amount of trans-diester **15**. The combined solids were dried under vacuum overnight (169 g, 76%): [α]_D_^25^ + 36.2 (c 1.02, CHCl_3_), Lit. value for its enantiomer: [α]_D_^27^ - 35.7 (c 1.4, CHCl_3_);^36^ ^**1**^ **H NMR** (500 MHz, CDCl_3_) δ 7.53 (d, *J* = 7.7 Hz, 1H), 7.32 (dt, *J* = 15.0, 7.5 Hz, 5H), 7.19 (dd, *J* = 22.2, 14.9 Hz, 2H), 7.12 (t, *J* = 7.4 Hz, 1H), 4.01 (dd, *J* = 8.9, 5.0 Hz, 1H), 3.93 (d, *J* = 4.2 Hz, 1H), 3.86 (d, *J* = 13.5 Hz, 1H), 3.77 (s, 3H), 3.59 (d, *J* = 13.5 Hz, 1H), 3.51 (s, 3H), 3.14 (dd, *J* = 15.7, 9.1 Hz, 1H), 3.03 (dd, *J* = 15.8, 4.8 Hz, 1H), 2.47–2.37 (m, 1H), 2.37–2.25 (m, 1H), 2.13–2.04 (m, 1H), 1.97 (td, *J* = 14.3, 7.0 Hz, 1H); ^**13**^ **C NMR** (126 MHz, CDCl_3_) δ 174.3, 173.4, 139.3, 136.2, 134.2, 129.1, 128.3, 127.1, 126.9, 121.8, 119.5, 118.2, 110.9, 107.5, 56.7, 54.6, 53.4, 51.9, 51.5, 29.8, 28.9, 21.2.

The spectroscopic data (^1^H and ^13^C NMR) and optical rotation for this compound **15** were in excellent agreement with that reported in the literature for its enantiomer.^30,37,44^

### Methyl (1*R*, 3*S*)-2-Benzyl-1-(3-methoxy-3-oxopropyl)-9-methyl-2,3,4,9-tetrahydro-1*H*-pyrido­[3,4-*b*]­indole-3-carboxylate (16)

The *N*_a_-H-*trans*-diaster **15** (172 g, 0.42 mol) and dry DMF (1100 mL) were added to a three-neck round-bottom flask (3000 mL) equipped with a reflux condenser. To the solution was added CH_3_I (66.1 g, 0.46 mol) with stirring, and then, the mixture was cooled to 0 °C using an ice bath. Then NaH [60% dispersion in mineral oil (11.1 g, 0.46 mol)] was added portionwise at 0 °C to minimize abrupt hydrogen gas evolution. The slurry which resulted was stirred at rt for 2 h until analysis by TLC (silica gel, EtOAc/hexane, 1:4) indicated the disappearance of starting material. The reaction solution was quenched by slow addition of CH_3_OH (100 mL) and then neutralized with a saturated aq solution of NH_4_CI, and this was followed by extraction with EtOAc (4 × 400 mL). The DMF was removed by washing the organic layer with water (5 × 100 mL). The combined organic layers were washed with brine and dried (K_2_CO_3_). The solvent was removed under reduced pressure, and the residue was purified by crystallization from EtOAc:hexane (50:50) to furnish *N*_a_-methyl *trans*-diester **16** (172.2 g, 96% yield), which was dried at rt under vacuum for 10 h: **[α]**_**D**_^**25**^ + 51.9 (c 1.02 CHCl_3_), Lit. value for its enantiomer: [α]_D_^25^ - 54.6 (c 1.0, CHCl_3_);^44^ ^**1**^ **H NMR** (500 MHz, CDCl_3_) δ 7.60 (d, *J* = 7.8 Hz, 1H), 7.35 (dt, *J* = 14.8, 7.3 Hz, 5H), 7.27 (ddd, *J* = 15.4, 7.6, 2.5 Hz, 2H), 7.17 (dd, *J* = 11.0, 3.8 Hz, 1H), 4.21–4.09 (m, 3H), 3.87 (s, 3H), 3.67 (s, 3H), 3.50 (s, 3H), 3.41 (d, *J* = 13.3 Hz, 1H), 3.13 (ddd, *J* = 20.9, 15.9, 8.1 Hz, 2H), 2.62 (ddd, *J* = 17.3, 9.4, 5.4 Hz, 1H), 2.42 (dt, *J* = 17.4, 5.5 Hz, 1H), 2.06–2.00 (m, 1H), 1.93–1.84 (m, 1H); ^**13**^ **C NMR** (126 MHz, CDCl_3_) δ 173.9, 173.3, 139.1, 137.5, 135.5, 129.4, 128.3, 127.1, 126.5, 121.4, 119.2, 118.2, 109.0, 106.3, 56.2, 53.5, 52.8, 52.1, 51.3, 29.8, 29.7, 27.9, 20.3. The spectroscopic data (^1^H and ^13^C NMR) and optical rotation for this compound **16** were in excellent agreement with that reported in the literature for its enantiomer.^30,37^

### Dieckmann Cyclization of *N*_a_-Methyl Trans Diaster (16) to Provide Methyl (6*R*,10*R*)-12-Benzyl-9-hydroxy-5-methyl-6,7,10,11-tetrahydro-5*H*-6,10-epiminocycloocta­[b]­indole-8-carboxylate (18)

In a 3-neck round-bottom flask (5 L), which contained 3300 mL of toluene [toluene had been predried by azeotropic removal of H_2_O by a DST (refluxed 8 h)], the *trans*-diaster **16** (202 g, 0.48 mol) was added. To the same reaction mixture was added NaH (59 g: 60% dispersion in mineral oil) at 0 °C carefully, which was followed by the slow addition of CH_3_OH (105 mL) dropwise under argon. The reaction mixture was allowed to warm to rt and stirred for 0.5 h. After this, the solution was heated to reflux for 5 h. While refluxing, the sides of the flask were covered with aluminum foil to maintain the reflux temperature without carbonizing any compound on the sides of the flask. After the disappearance of the starting material (confirmed by analysis by TLC (silica gel, EtOAc/hexane, 1:4)), the reaction solution was quenched with cold water (300 mL). The aq layer was extracted with CH_2_CI_2_ (4 × 1500 mL), and the combined organic layers were washed with brine (2 × 200 mL) and dried (K_2_CO_3_). The solvent was removed under reduced pressure, and the mineral oil was separated by decantation. The residue which resulted was dried under a vacuum at 40 °C (10 h) and then used directly in the next step (acidic hydrolysis) without further purification and characterization.

### Acid-Mediated Hydrolysis and Decarboxylation of Methyl (6*R*,10*R*)-12-Benzyl-9-hydroxy-5-methyl-6,7,10,11-tetrahydro-5*H*-6,10-epiminocycloocta­[b]­indole-8-carboxylate (18) to Provide (6*R*,10*R*)-12-Benzyl-5-methyl-5,6,7,8,10,11-hexahydro-9*H*-6,10-epiminocycloocta­[b]­indol-9-one (17)

To a 3-neck round-bottom flask (5000 mL) containing the β-keto ester **18** (196 g, 0.5 mol), glacial acetic acid (702 mL), aq hydrochloric acid (1000 mL, conc.), and water (280 mL) were added. The solution which resulted was stirred and held at reflux for 8 h. The solvent (mostly acid) was removed carefully under reduced pressure (in a one neck 3000 mL round-bottom flask is easier on this scale with a Keck clip). The residue was brought to pH 9 with the addition of a cold aqueous solution of NaOH (3 *N*) at 0 °C dropwise. The mixture which resulted was extracted with CH_2_Cl_2_ (4 × 1500 mL), and the combined organic extracts were washed with a saturated aq solution of NH_4_Cl (2 × 400 mL), brine (2 × 300 mL), and dried (K_2_CO_3_). The product was purified by crystallization from EtOAc/hexane (1:4, 500 mL). The mother liquor was concentrated under reduced pressure, and the residue was purified by column chromatography (silica gel with EtOAc/hexane, 1:4) to provide additional tetracyclic ketone **17**. The solid was dried under vacuum at rt for 8 h to yield the tetracyclic ketone **17** (145 g, 88%): **[α]**_**D**_^**25**^ + 236.6 (c 1.0, CHCl_3_), Lit. value for its enantiomer: [α]_D_^27^ – 240.2 (c 1.0, CHCl_3_);^30^ ^**1**^ **H NMR** (300 MHz, CDCl_3_) δ 7.58 (d, *J* = 7.7 Hz, 1H), 7.41–7.28 (m, 7H), 7.20 (t, *J* = 7.3 Hz, 1H), 4.13 (s, 1H), 3.87–3.76 (m, 3H), 3.66 (s, 3H), 3.32 (dd, *J* = 16.9, 6.8 Hz, 1H), 2.76 (d, *J* = 16.9 Hz, 1H), 2.60–2.46 (m, 2H), 2.28–1.95 (m, 2H); ^**13**^ **C NMR** (75 MHz, CDCl_3_) δ 210.0, 138.3, 137.3, 133.2, 128.7, 128.5, 127.4, 126.5, 121.6, 119.3, 118.3, 109.0, 105.8, 64.9, 56.3, 48.9, 34.4, 29.8, 29.4, 20.5; **HRMS** (ESI) *m*/*z* (M + H)^+^ calcd for C_22_H_22_N_2_O, 331.1805, found 331.1772. The spectroscopic data (^1^H and ^13^C NMR), and optical rotation for this compound **17** were in excellent agreement with that reported in the literature for its enantiomer.^30,37,42^

### Catalytic Debenzylation of (6*R*,10*R*)-12-Benzyl-5-methyl-5,6,7,8,10,11-hexahydro-9*H*-6,10-epiminocycloocta­[b]­indol-9-one (17) to Provide (6*R*,10*R*)-5-Methyl-5,6,7,8,10,11-hexahydro-9*H*-6,10-epiminocycloocta­[b]­indol-9-one (4)

To a round-bottom flask, which contained a solution of *N*_b_-benzyl tetracyclic ketone **17** (43.3 g, 0.13 mol) in anhydrous (200 proof) EtOH (390 mL), with stirring, was added the acetyl chloride (16.5 g, 0.21 mol) premixed with 150 mL of anhydrous EtOH dropwise at 0 °C. The solid starting indole was completely dissolved in ethanol after this addition. The solvent was removed under reduced pressure and at a moderate temperature (water bath temperature 40 °C), anhydrous diethyl ether (2 × 100 mL) was poured into the residue and then the diethyl ether was removed under reduced pressure. Finally, the HCl salt, which remained, was dissolved in anhydrous EtOH (2 × 60 mL), and the EtOH was evaporated under reduced pressure to remove excess HCl. The repetition of this step 5 times was crucial to make sure no excess HCl remained in the residue. This process was followed by degassing of the hydrochloride salt at rt under reduced pressure, and it was backfilled with argon (3 times). Then dry EtOH (400 mL) and dry Pd/C (10% by wt, 13 g) were added to the above hydrochloride salt. The mixture which resulted was degassed under reduced pressure at rt and backfilled with H_2_ gas three times (H_2_ gas was filled in a balloon). The mixture which resulted was stirred at rt under an atmosphere of H_2_ (1 atm) for 10 h or until the disappearance of starting material on analysis by TLC (EtOAc with a few drops of MeOH and 10% aq NH_4_OH). **The catalyst was filtered off through a short pad of Celite, and the solid, which was filtered off, was washed with EtOH (3 × 90 mL). The solid material was then discarded safely**. The solvent collected was removed under a reduced pressure. The residue was dissolved in CH_2_Cl_2_, and the free base was prepared by dropwise addition of 10% aq NH_4_OH. The layers were separated and the aq layer was washed with CH_2_Cl_2_ (2 × 500 mL), and the combined layer was washed with brine (2 × 300 mL) and dried with K_2_CO_3_. The solvent was removed under reduced pressure to yield *N*_a_-Me,*N*_b_-H tetracyclic ketone **4** (27 g, 86%) as a pure solid (no further purification was required), which was dried under vacuum before using it in the next step: ^**1**^ **H NMR** (300 MHz, CDCl_3_) δ 7.50 (d, *J* = 7.8 Hz, 1H), 7.38–7.21 (m, 2H), 7.15 (t, *J* = 7.3 Hz, 1H), 4.37 (s, 1H), 3.95 (d, *J* = 6.7 Hz, 1H), 3.63 (s, 3H), 3.14 (dd, J = 16.5, 6.8 Hz, 1H), 2.84 (d, *J* = 16.5 Hz, 1H), 2.49 (dt, *J* = 9.7, 4.2 Hz, 2H), 2.12 (dd, *J* = 14.9, 7.4 Hz, 2H); ^**13**^ **C NMR** (75 MHz, CDCl_3_) δ 210.7, 137.0, 135.2, 126.5, 121.6, 119.3, 118.1, 108.9, 106.4, 59.7, 44.9, 34.9, 31.4, 29.3, 25.8; **HRMS** (ESI) *m*/*z* (M + H)^+^ calcd for C_19_H_16_N_2_O, 293.1648, found 293.1614. The spectroscopic data (^1^H and ^13^C NMR) of this compound **4** were in excellent agreement with that reported in the literature for its enantiomer.^30,37^

### Preparation of (6*R*,10*R*)-5-Methyl-12-((*R*)-4-(triisopropylsilyl)­but-3-yn-2-yl)-5,6,7,8,10,11-hexahydro-9*H*-6,10-epiminocycloocta­[b]­indol-9-one (22)

To an oven-dried 2 L round-bottom flask, *N*_a_-CH_3_,*N*_b_-H tetracyclic ketone **4** (24.4 g, 0.1 mol) was added, and this was followed by the addition of dry acetonitrile (400 mL). To the solution was added (*S*)-4-(triisopropylsilyl)­but-3-yn-2-yl 4-methylbenzenesulfonate **21** (50.2 g, 0.116 mol) at rt, with stirring. At this point, anhydrous potassium carbonate (17.27 g, 0.132 mol) was added into the reaction mixture. The reaction mixture which resulted was stirred at 75 °C (outside oil bath temperature) for 12 h under argon. After 12 h, the analysis by TLC (silica gel, EtOAc/a few drops of 10% aqueous NH_4_OH) indicated the absence of starting material (tetracyclic ketone **4**). The reaction mixture was cooled to rt, and the solid (K_2_CO_3_) was removed by filtration of the solution through a bed of Celite using EtOAc as the solvent. The solvent was removed under reduced pressure, after which the crude product was purified by flash chromatography (silica gel, EtOAc/hexanes, 1:10) to provide the (*R*)-*N*_a_-CH_3_, *N*_b_-TIPS protected acetylenic tetracyclic ketone **22** as a light yellow solid (41.9 g, 91%), which was dried under vacuum at rt for 14 h. The desired stereochemistry of this product was confirmed after the removal of TIPS group in the next step.

Analysis of **22**; ^**1**^ **H NMR** (500 MHz, CDCl_3_) δ 7.45 (d, *J* = 7.8 Hz, 1H), 7.30 (d, *J* = 8.2 Hz, 1H), 7.21 (ddd, J = 8.2, 7.1, 1.1 Hz, 1H), 7.14–7.08 (m, 1H), 4.90 (m, *J* = 2.5 Hz, 1H), 3.96 (d, *J* = 6.5 Hz, 1H), 3.67 (s, 3H), 3.64 (q, *J* = 6.6 Hz, 1H), 3.13 (dd, *J* = 16.7, 6.6 Hz 1H) 2.69 (d, *J* = 16.3 Hz, 1H), 2.63–2.52 (m, 1H), 2.50–2.43 (m, 1H), 2.19–1.97 (m, 2H), 1.46 (d, *J* = 6.6 Hz, 3H), 1.03 (d, *J* = 5.6 Hz, 21H); ^**13**^ **C NMR** (126 MHz, CDCl_3_) δ 210.6, 137.4, 133.4, 126.6, 121.7, 119.4, 118.5, 108.9, 107.4, 107.0, 85.5, 63.2, 47.7, 47.5, 34.6, 29.4, 29.3, 22.2, 21.2, 18.7, 11.3. The ^1^H and ^13^C NMR data of compound **22** were in complete agreement with that reported in the literature for its enantiomer.^30,37^

### Preparation of (6*R*,10*R*)-12-((*R*)-but-3-yn-2-yl)-5-Methyl-5,6,7,8,10,11-hexahydro-9*H*-6,10-epiminocycloocta­[b]­indol-9-one (19)

To an oven-dried 3 L round-bottom flask equipped with a dropping funnel was added the TIPS protected ethynyl tetracyclic ketone **22** (73.1 g, 0.16 mol). This was followed by the addition of THF (675 mL) and TBAFxH_2_O (245 mL, 1.0 M solution in THF) at 0 °C dropwise via a dropping funnel. During the addition, the wine-red colored solution turned to a black color. After the reaction mixture was stirred at 0 °C for 0.5 h, the ice bath was removed, and the mixture was stirred at rt for an additional 4 h or until the analysis by TLC (silica gel, EtOAC/hexane, 1:4) confirmed the disappearance of the starting material **22**. The reaction was then quenched with H_2_O (400 mL), and the reaction mixture was diluted with EtOAc (1200 mL). The two layers were separated. The organic layer was washed with water (2 × 200 mL) and then washed with brine (2 × 250 mL) and dried (Na_2_SO_4_). The organic solvent (EtOAc) was removed under reduced pressure to 1/3 of its original volume, and the slurry was allowed to stay at rt overnight in a beaker for crystallization. The mother liquor, which remained after crystallization, was purified by passing through a short pad of silica gel to yield an additional amount of *N*_a_-Me, ethynyl tetracyclic ketone **19** as a light yellow colored solid (46.6 g, 98% yield), which was vacuum-dried at rt overnight. The optical rotation, proton NMR, carbon NMR, and X-ray crystallography indicated that the pure compound had the desired stereochemistry (*R*,*R*,*R*) **[α]**_**D**_^**25**^ + 191.3 (c 1.0, CHCl_3_), Lit. value for its enantiomer: [α]_D_^27^ – 191.0 (c 0.192, CHCl_3_);^42^ ^**1**^ **H NMR** (500 MHz, CDCl_3_) δ 7.46 (d, *J* = 7.8 Hz, 1H), 7.31 (d, *J* = 8.2 Hz, 1H), 7.22 (t, *J* = 7.6 Hz, 1H), 7.11 (t, *J* = 7.4 Hz, 1H), 4.75 (m, *J* = 2.5 Hz, 1H), 3.95 (d, *J* = 6.6 Hz, 1H), 3.70 (s, 3H), 3.61 (qd, *J* = 6.6, 2.1 Hz, 1H), 3.17–3.09 (m, 1H), 2.70 (d, *J* = 16.7 Hz, 1H), 2.61 (m, *J* = 11.4, 8.7, 5.5 Hz, 1H), 2.48 (m, *J* = 17.0, 6.2 Hz, 1H), 2.28 (d, *J* = 2.2 Hz, 1H), 2.18–2.03 (m, 2H), 1.45 (d, *J* = 6.7 Hz, 3H); ^**13**^ **C NMR** (126 MHz, CDCl_3_) δ 210.3, 137.2, 133.6, 126.3, 121.6, 119.3, 118.8, 108.9, 106.2, 84.7, 72.5, 61.3, 49.3, 47.5, 34.5, 29.4, 29.0, 21.5, 20.4; HRMS (ESI) *m*/*z* (M + H)^+^ calcd for C_19_H_20_N_2_O, 293.1164, found 293.1614. The spectroscopic data (^1^H and ^13^C NMR) and optical rotation (compared to the literature value of its enantiomer) of this compound **29** were in complete agreement with that reported in the literature for its enantiomer.^30,37^

### Preparation of (6*R*,10*R*)-12-((*R*)-3-Iodobut-3-en-2-yl)-5-methyl-5,6,7,8,10,11-hexahydro-9*H*-6,10-epiminocycloocta­[b]­indol-9-one (23) via Haloboration and Protodeboronation of the Ethynyl Tetracyclic Ketone (19)

An oven-dried round-bottom flask (1000 mL) equipped with an addition funnel was cooled under the continuous flow of argon. The *N*_a_-Me ethynyl tetracyclic ketone **19** (5.1 g, 17.4 mmol) was dissolved in anhydrous CH_2_CI_2_ (130 mL) and hexanes (17 mL) and then charged into the flask. Using an ice bath, the flask was cooled to 0 °C (outside temperature), and I–B­(Cy)_2_ (73.4 mL, 136.4 mmol, 0.5 M solution in hexanes) was added dropwise every 0.5 h in three portions. The mixture which resulted after the last addition was stirred at 0 °C for another 0.5 h, and then the ice bath was removed. The mixture was then allowed to warm to rt and stirred for 2.0 h. This was followed by the addition of another 0.5 equiv of I–B­(Cy)_2_ (18.4 mL, 9.2 mmol) dropwise into the mixture at rt. Then the reaction mixture was allowed to stir for an additional 2 h at rt. At this point, glacial acetic acid (4.8 mL, 201.8 mmol) was added into the solution at 0 °C (ice bath), and the mixture was allowed to stir at rt for an additional 1.2 h. To this solution, cold aq 3 M NaOH (97.9 mL, 293.8 mmol) and 30% H_2_O_2_ (6.3 mL, 55.8 mmol) were added with stirring and the temperature was lowered to 0 °C (ice bath); the mixture which formed was allowed to stir for an additional 1.0 h at rt.

The biphasic solution which resulted was transferred into a larger flask (3000 mL) and diluted with CH_2_CI_2_ (1100 mL) and water (170 mL). The two layers were separated. The residual solid, which remained in the original flask (attached to the bottom of the flask), was dissolved in acetone (250 mL), and the solvent was removed under reduced pressure to 1/3 of the original volume. The mixture which resulted was diluted with CH_2_Cl_2_ (150 mL), and the biphasic layers were separated again. The combined CH_2_CI_2_ layers were treated with solutions of 5% KF in methanol (390 mL) and 5% aqueous sodium bisulfite (390 mL) and stirred vigorously for 10 min. The aq layer was separated and extracted with CH_2_CI_2_ (2 × 250 mL), after which the combined organic layers were washed with brine (2 × 200 mL) and dried (Na_2_SO_4_). The organic solvents were removed under reduced pressure, and the crude material was passed through a short column, which contained a pad of silica gel (ethyl acetate/hexanes, 1:4) to furnish the vinyl iodide **23** as an off-white colored solid (5.8 g, 80%): *R*_*f*_ 0.4 (EtOAc/hexane, 1:4); **[α]**_**D**_^25^ + 166.36 (c 1.07, CHCl_3_); ^**1**^ **H NMR** (500 MHz, CDCl_3_) δ 7.48 (d, *J* = 7.8 Hz, 1H), 7.31 (d, *J* = 8.2 Hz, 1H), 7.25–7.20 (m, 1H), 7.13 (dd, *J* = 10.9, 3.9 Hz, 1H), 6.22 (s, 1H), 5.86 (s, 1H), 4.25 (s, 1H), 4.01 (d, *J* = 6.3 Hz, 1H), 3.64 (s, 3H), 3.12 (dd, *J* = 16.8, 6.6 Hz, 1H), 2.77 (d, *J* = 16.7 Hz, 1H), 2.55 (dd, *J* = 33.7, 19.7 Hz, 3H), 2.18–2.07 (m, 1H), 2.03–1.96 (m, 1H), 1.19 (d, *J* = 6.1 Hz, 3H); ^**13**^ **C NMR** (126 MHz, CDCl_3_) δ 210.4, 137.3, 133.2, 126.5, 125.9, 122.7, 121.7, 119.4, 118.3, 109.0, 106.7, 62.5, 60.9, 47.8, 34.5, 29.8, 29.4, 21.3, 19.9; **HRMS** (ESI) *m*/*z* (M + H)^+^ calcd for C_19_H_21_IN_2_O, 421.0771, found 421.0777. The spectroscopic data (^1^H and ^13^C NMR) of compound **33** were in excellent agreement with that reported in the literature for its enantiomer.^30^

### Cu-Mediated Cross-Coupling Reaction of the Vinyl Iodide (23) to Provide (6*R*,7*R*,8*R*,10*R*,11a*R*)-5,8-Dimethyl-9-methylene-5,6,9,10,11a,12-hexahydro-6,10-methanoindolo­[3,2-*b*]­quinolizin-11­(8H)-one (11)

The pentacyclic ketone **11** was synthesized by following the published procedure.^33^ The vinyl iodide **33** (3 g, 7.13 mmol), CuI (1.36, 7.13 mmol), *cis* 1,2-cyclohexanediol (0.83 g, 7.13 mmol), and Cs_2_CO_3_ (9.3 g, 28 mmol) were added into a sealed tube, and then dry DMF (40 mL) was added to it. The mixture which resulted was degassed at rt under reduced pressure and backfilled with argon 4 times. The reaction mixture was then put in a preheated oil bath at 135–140 °C and allowed to stir for 10 h. The progress of the reaction was monitored by TLC (neutral alumina, EtOAC/hexane, 6:4 plus a few drops of 10% aqueous NH_4_OH); this confirmed no starting material remained. The reaction mixture was allowed to reach rt and diluted with EtOAc (50 mL) and water (25 mL). The biphasic layers were separated, and the aq layer was extracted with EtOAc (3 × 40 mL). The combined organic layers were washed with water (2 × 40 mL) and brine (3 × 40 mL) and dried (Na_2_SO_4_). The solvent was removed under reduced pressure, and the residue was purified by flash chromatography using neutral alumina (EtOAc/hexane = 1:3) to furnish the pentacyclic ketone **11** as an off-white colored solid, which was crystallized from EtOAc (100%) for X-ray crystallography. **[α]**_**D**_^**25**^ + 146.7 (c 0.98, CHCl_3_), Lit. value for its enantiomer: [α]_D_^25^ – 150.0 (c 1.0, CHCl_3_);^34^ ^**1**^ **H NMR** (500 MHz, CDCl_3_) δ 7.52 (d, *J* = 7.8 Hz, 1H), 7.28 (d, *J* = 8.4 Hz, 1H), 7.21 (dd, *J* = 11.2, 3.9 Hz, 1H), 7.11 (t, *J* = 7.4 Hz, 1H), 5.15 (d, *J* = 2.6 Hz, 1H), 5.03 (d, *J* = 2.2 Hz, 1H), 4.49 (d, 1H), 3.93 (qd, *J* = 6.7, 4.4 Hz, 1H), 3.75 (d, *J* = 5.6 Hz, 1H), 3.62 (s, 3H), 3.37 (d, *J* = 15.5 Hz, 1H), 3.08 (dd, *J* = 3.3, 1.8 Hz, 1H), 2.95 (dd, *J* = 15.5, 6.1 Hz, 1H), 2.69–2.57 (m, 1H), 2.21–2.10 (m, 1H), 1.52 (d, *J* = 6.8 Hz, 3H); ^**13**^ **C NMR** (126 MHz, CDCl_3_) δ 217.6, 148.2, 137.5, 137.3, 126.6, 121.4, 119.1, 118.6, 111.5, 108.7, 105.1, 58.7, 57.9, 51.9, 51.0, 35.8, 29.3, 22.5, 17.5; **HRMS** (ESI) *m*/*z* (M + H)^+^ calcd for C_19_H_20_N_2_O, 293.1648, found 293.1641. The spectroscopic data (^1^H and ^13^C NMR) of this compound **11** were in complete agreement with that reported in the literature for its enantiomer.^33^ In addition, analysis by X-ray crystallography confirmed the absolute structure and stereochemistry, as shown above.

### Conversion of the Pentacyclic Ketone (11) into ((6*R*,7*R*,8*R*,10*S*,11*S*,11a*R*)-5,8-Dimethyl-9-methylene-5,6,8,9,10,11,11a,12-octahydro-6,10-methanoindolo­[3,2-*b*]­quinolizin-11-yl)­methanol (26)

In a 2-neck round-bottom flask (1000 mL), the mixture of methoxymethyltriphenylphosphonium chloride (44.8 g, 130.2 mmol), anhydrous potassium tert-butoxide (16 g, 141.73 mmol), and dry benzene (350 mL) was stirred for 1 h at rt. Benzene was dried by pre-distilling in the presence of CaH_2_ (dry benzene from Sigma-Aldrich also works). After 1 h of stirring, the yellow mixture then turned to a dark red color, and the temperature of the mixture was lowered to 0 °C. The pentacyclic ketone **11** (5.6 g, 19.15 mmol) was dissolved in a minimum amount of dry THF (50 mL) and added to the above solution dropwise. The reaction mixture which resulted was allowed to warm to rt and allowed to stir overnight. After stirring overnight, analysis by TLC (silica gel, 0.3 mL of MeOH + 4.7 mL of CH_2_CI_2_) confirmed the disappearance of starting material and the formation of a new component (less polar methyl enol ether intermediate **24**). At this point, the reaction mixture was diluted by adding EtOAc (200 mL) and quenched with water (100 mL). The biphasic layers were separated by extraction and the aq layer was extracted with EtOAc (2 × 200 mL). The combined organic layers were washed with brine (2 × 150 mL) and dried (Na_2_SO_4_). The evaporation of organic solvents under reduced pressure resulted in a brownish-red oily material, which was used in the next step (acidic hydrolysis) without further characterization or purification. Then aq 12 *N* cone HCl (140 mL) and THF (140 mL) were added to the above mixture of crude oil, and the mixture which resulted was stirred for 8 h at 55–60 °C (oil bath temperature). The analysis by TLC (silica gel, 0.3 mL of MeOH + 4.7 mL of CH_2_CI_2_) indicated the disappearance of the methyl enol ether **24** component and the appearance of an aldehyde component **25** [lower *R*_*f*_ value than starting material (methyl enol ether **24**)]. The reaction mixture was cooled to rt, and the volume of the solvent was reduced to 1/3 of the original volume by removing the solvents under reduced pressure. At this point, the phosphorus impurities were removed by adding hexane (70 mL), vigorously shaking, and then decanting the upper hexane layer. The process was repeated 6 times. The mixture which resulted was then brought to pH 8 by adding 14% aq NH_4_OH dropwise at 0 °C. The aq layer was extracted with EtOAc (4 × 100 mL). The organic layer was washed with brine (2 × 60 mL) and dried (Na_2_SO_4_). The similar *R*_*f*_ value by analysis of TLC comparison with an authentic sample of an enantiomeric aldehyde (the stereochemistry of which was confirmed by X-ray crystallography analysis earlier) indicated the α stereochemistry in aldehyde **25**.^30,44^ The organic solvents were removed under reduced pressure, which resulted in a crude aldehyde **25** (6 g) as a brown oil, which was further dried under high vacuum for 7 h. It was used directly in the next step without further purification or characterization. The crude aldehyde (6 g) was placed in a one-neck round-bottom flask (500 mL) and was dissolved in dry (200 proof) EtOH (95 mL). The temperature of the reaction mixture was lowered to 0 °C using an ice bath. To this solution, NaBH_4_ (0.88 g, 0.023 mol) was added in one portion at 0 °C, and the mixture which resulted was stirred for 6 h at the same temperature (0 °C). Then analysis by TLC (silica gel, 0.3 mL of MeOH + 4.7 mL of CH_2_CI_2_) confirmed the formation of the more polar component (*R*_*f*_ 0.3) and by absence of starting material (aldehyde **35**). At this point, the reaction mixture was diluted by the addition of CH_2_Cl_2_ (250 mL) and cold water (50 mL). The biphasic layers were separated, and the aqueous layer was extracted with CH_2_Cl_2_ (3 × 40 mL). The combined organic layer was washed with brine (2 × 40 mL) and dried (Na_2_SO_4_). The organic solvents were removed under reduced pressure, and the crude waxy solid which resulted was purified on column chromatography (silica gel, 5–7% CH_3_OH in CH_2_Cl_2_) to provide alcohol **26** as waxy solid (4.48 g, 82%): **[α]**_**D**_^25^ + 62.0 (c 0.5, CHCl_3_); ^**1**^ **H NMR** (500 MHz, CDCl_3_) δ 7.38 (d, *J* = 7.8 Hz, 1H), 7.29 (d, *J* = 5.7 Hz, 1H), 7.22–7.17 (t, 1H), 7.12–7.05 (t, 1H), 4.89 (d, *J* = 1.9 Hz, 2H), 4.25–4.20 (m, 1H), 3.63 (s, 4H), 3.52–3.42 (m, 2H), 3.03–2.96 (m, 1H), 2.93 (dd, J = 15.2, 5.2 Hz, 1H), 2.60 (d, *J* = 15.1 Hz, 1H), 2.34–2.29 (br, s, 2H), 2.08 (ddd, *J* = 12.1, 10.0, 2.0 Hz, 1H), 1.65–1.59 (q, 1H), 1.55–1.48 (m, 1H), 1.39 (d, *J* = 6.8 Hz, 3H); ^**13**^ **C NMR** (126 MHz, CDCl_3_) δ 152.6, 139.4, 137.4, 127.5, 120.8, 118.8, 118.2, 108.7, 107.8, 104.1, 64.9, 58.2, 51.0, 48.2, 44.6, 36.1, 33.2, 29.4, 27.2, 16.9; **HRMS** (ESI) *m*/*z* (M + H)^+^ calcd for C_21_H_24_N_2_O, 309.1961, found 309.1935. The spectroscopic data (^1^H and ^13^C NMR) of this compound **26** were in excellent agreement with that reported in the literature for its enantiomer.^36^

### Conversion of the Pentacyclic Alcohol (26) into (6*R*,7*R*,8*R*,10*S*,11*S*,11a*R*)-5,8-Dimethyl-9-methylene-11-(((triisopropylsilyl)­oxy) methyl)-5,6,8,9,10,11,11a,12-octahydro-6,10-methanoindolo­[3,2-*b*]­quinolizine (27)

To pentacyclic alcohol **26** (4.2 g, 0.013 mol) in dry CH_2_Cl_2_ (100 mL), 2,6-lutidine (4.4 g, 0.04 mol) was added at 0 °C with stirring. To this solution was added TIPSOTF (4.3 g, 0.04 mol) at 0 °C. The mixture was then allowed to stir for an additional 6 h at 0 °C. At this point, the analysis by TLC indicated the absence of starting material and the presence of a new less polar component (silica gel, 2–4% MeOH in CH_2_CI_2_), after which the reaction was quenched by adding cold water (10 mL). The reaction mixture was diluted with CH_2_CI_2_ (300 mL) and cold water (50 mL). The aq layer was extracted with CH_2_CI_2_ (2 × 50 mL), and the combined organic layer was washed with brine (100 mL) and dried (Na_2_SO_4_). After removal of the solvents under reduced pressure, the crude waxy solid was dried under reduced pressure (at rt for 10 h) to remove the excess 2,6-lutidine. The solid which resulted was then purified by flash chromatography (silica gel, CH_2_CI_2_/CH_3_OH, 20:1) to provide the silyl ether **27** as a white colored waxy solid: *R*_*f*_ 0.4 (silica gel, 5% MeOH in CH_2_CI_2_); **[α]**_**D**_^25^ −23.6 (c 1.4, CHCl_3_); ^**1**^ **H NMR** (500 MHz, CDCl_3_) δ 7.48 (d, *J* = 7.7 Hz, 1H), 7.28 (d, *J* = 8.2 Hz, 1H), 7.18 (ddd, *J* = 8.2, 7.1, 1.2 Hz, 1H), 7.08 (td, *J* = 7.5, 1.0 Hz, 1H), 4.94–4.87 (m, 2H), 4.27 (dd, *J* = 9.8, 2.1 Hz, 1H), 3.69–3.63 (m, 4H), 3.61–3.55 (m, 2H), 2.98 (dt, *J* = 15.4, 5.3 Hz, 2H), 2.71 (s, 1H), 2.46–2.42 (m, 1H), 2.15 (ddd, *J* = 12.2, 10.0, 2.1 Hz, 1H), 1.80 (dd, *J* = 14.6, 7.0 Hz, 1H), 1.67 (ddd, *J* = 12.3, 3.7, 2.8 Hz, 1H), 1.42 (d, *J* = 6.8 Hz, 3H), 1.04 (dd, *J* = 5.1, 3.0 Hz, 21H); ^**13**^ **C NMR** (126 MHz, CDCl_3_) δ 152.5 (C), 139.7 (C), 137.3 (C), 127.6 (C), 120.7 (CH), 118.7 (CH), 118.1 (CH), 108.6 (CH), 107.6 (CH_2_), 104.3 (C), 65.6 (CH_2_), 58.3 (CH), 51.2 (CH), 48.7 (CH), 44.6 (CH), 35.8 (CH), 33.4 (CH_2_), 29.3 (CH_3_), 27.4 (CH_2_), 18.1 (6 X CH_3_), 17.0 (CH_3_), 11.9 (3 X CH); **DEPT 135** ^**13**^ **C NMR**, (126 MHz, CDCl_3_) δ 120.7 (CH), 118.7 (CH), 118.2 (CH), 108.6 (CH), 107.6 (CH_2_), 65.6 (CH_2_), 58.3 (CH), 51.2 (CH), 48.7 (CH), 44.6 (CH), 35.9 (CH), 33.4 (CH_2_), 29.3 (CH_3_), 27.4 (CH_2_), 18.1 (6 X CH_3_), 17.0 (CH_3_), 11.98 (3 X CH); **HRMS** (ESI) *m*/*z* (M + H)^+^ for C_29_H_44_N_2_OSi, calcd 465.3295, found 465.3299. The spectroscopic data (^1^H and ^13^C NMR) of this compound **27** were in excellent agreement with that reported in the literature for its enantiomer.^36^

### Conversion of the Alkene (27) into ((6*R*,7*S*,8*R*,9*R*,10*R*,11*S*,11a*R*)-5,8-Dimethyl-11-(((triisopropylsilyl)­oxy)­methyl)-5,6,8,9,10,11,11a,12-octahydro-6,10-methanoindolo­[3,2-*b*]­quinolizin-9-yl)­methanol (28)

To the solution of alkenyl TIPS ether **27** (3.5 g, 7.53 mmol) in dry THF (50 mL) was added 2.0 M solution of BH_3_-DMS in THF (67.7 mmol, 34.3 mL) with stirring at rt, and the mixture was allowed to stir for 2.5 h at rt.

After the reaction mixture was allowed to stir for 2.5 h, ice cold water (125 mL) was used to quench the reaction at 0 °C (added slowly because the addition of water results in a large amount of effervescence of H_2_ gas at the beginning). To the reaction mixture was added NaBO_3_·4H_2_O (28 g, 180.7 mmol) in one portion at 0 °C. The mixture which resulted was allowed to warm to rt and stirred at rt for an additional 2 h. At this point, EtOAc (200 mL) and H_2_O (90 mL) were added to the reaction mixture. The biphasic layers were separated, and the organic layer was washed with water (2 × 90 mL) and brine (2 × 80 mL) and dried (Na_2_SO_4_). The solvent was then removed under reduced pressure to provide the *N*_b_-BH_3_ complex as a mixture of isomers at [C(20)] which was dried under a vacuum for 6 h prior to use in the next step. The majority of the mixture was desired primary alcohol **28**. This crude reaction mixture was used in the next step without any further purification or characterization. To the mixture of isomers, dry MeOH (70 mL) and Na_2_CO_3_ (4 g, 37.7 mmol) was added, and the mixture was then warmed to 60 °C (oil bath) for 6 h. It was stirred vigorously. At this point, the reaction mixture was allowed to cool to room temperature, and this was followed by the removal of the solids by filtration through a bed of Celite. The filtrate which resulted was concentrated under reduced pressure to provide a crude oily material, which was then redissolved in CH_2_CI_2_. The CH_2_CI_2_ layer was washed with H_2_O (50 mL), brine (4 × 40 mL) and dried (Na_2_SO_4_). The solvent was removed under reduced pressure to afford a crude waxy solid, which was purified by flash chromatography [silica gel, CH_2_Cl_2_/MeOH (v/v 10:1)] to provide primary alcohol **28**, in 61% yield (2.2 g).

*R*_*f*_ 0.3 (basic alumina, EtOAc/hexanes, 1:1 plus few drops of Et_3_N) **[α]**_**D**_^25^ - 63.6 (c 1.1, CHCl_3_); ^**1**^ **H NMR** (500 MHz, CDCl_3_) δ 7.48 (d, *J* = 7.8 Hz, 1H), 7.31 (d, *J* = 8.2 Hz, 1H), 7.24–7.17 (m, 1H), 7.13–7.07 (m, 1H), 4.17 (dt, *J* = 16.0, 8.0 Hz, 1H), 3.91 (dd, *J* = 10.5, 8.1 Hz, 1H), 3.81 (dd, *J* = 8.7, 8.0 Hz, 2H), 3.74 (t, *J* = 10.0 Hz, 1H), 3.66 (s, 3H), 3.39 (dq, *J* = 10.7, 7.3 Hz, 1H), 3.20 (dd, *J* = 8.2, 5.1 Hz, 1H), 3.04–2.95 (m, 1H), 2.69 (d, *J* = 14.9 Hz, 1H), 2.25 (s, 1H), 2.07–1.91 (m, 2H), 1.84 (q, *J* = 8.6 Hz, 1H), 1.62–1.52 (m, 1H), 1.33 (d, *J* = 7.3 Hz, 3H), 1.05 (t, *J* = 7.0 Hz, 21H); ^**13**^ **C NMR** (126 MHz, CDCl_3_) δ 139.4, 137.2, 127.6, 120.7, 118.7, 118.1, 108.7, 104.0, 67.0, 63.2, 54.2, 51.4, 48.8, 41.2, 40.1, 36.5, 29.3, 27.7, 24.9, 18.0, 13.3, 11.9; ^**13**^ **C NMR DEPT** 135 (126 MHz, CDCl_3_) δ 120.7 (CH), 118.7 (CH), 118.1 (CH), 108.7 (CH), 67.0 (CH_2_), 63.2 (CH_2_), 54.2 (CH), 51.4 (CH), 48.8 (CH), 41.2 (CH), 40.1 (CH), 36.55 (CH_2_), 29.32 (CH_3_), 27.7 (CH_2_), 24.9 (CH), 18.0 (CH3 × 6), 13.3 (CH_3_), 11.9 (CHX3); **HRMS** (ESI) *m*/*z* (M + H)^+^ calcd for C_29_H_46_N_2_O2Si, 483.3401, found 483.3410. The NMR spectroscopic data of the desired alcohol **26** were in excellent agreement with that reported in the literature for its enantiomer.^34,36^

### Corey Kim Oxidation of Alcohol (28) to Provide 6*R*,7*S*,8*R*,9*R*,10*S*,11*S*,11a*R*)-5,8-Dimethyl-11-(((triisopropylsilyl)­oxy) methyl)-5,6,8,9,10,11,11a,12-octahydro-6,10-methanoindolo­[3,2-*b*]­quinolizine-9-carbaldehyde (29)

To a solution of *N*-chlorosuccinimide (415 mg, 3.6 mmol) in dry CH_2_CI_2_ (20 mL), with stirring, was added dimethyl sulfide (0.5 mL, 1.45 mmol) at −5 to −15 °C dropwise under a continuous flow of argon. A white precipitate was observed soon after the addition of dimethyl sulfide, which was the Corey Kim reagent. At this point, the mixture which resulted was stirred for 0.7 h in the temperature range mentioned above. After this, the temperature of the reaction mixture was further lowered (−78 °C, EtOAc-dry ice bath), and the alcohol (500 mg, 0.2 mmol) in dry CH_2_C1_2_ (10 mL) was added into the reaction mixture under argon. Before the addition of the starting material, the solution of starting material (**28**) in CH_2_Cl_2_ was also cooled to −78 °C. The mixture which resulted was stirred for 3 h at −78 °C. At this point, dry triethylamine (420 mg, 4.1 mmol) was then added to the above mixture dropwise, and the stirring was allowed to continue for an additional 1.5 h at the same temperature (−78 °C). The completion of the reaction was confirmed by analysis on TLC (basic alumina, MeOH/CH_2_Cl_2_, 1:9). The reaction mixture was quenched at −78 °C by the addition of CH_2_CI_2_ (30 mL) and H_2_O (10 mL). The reaction mixture which resulted was allowed to stir and warmed to rt by removing the cooling bath.

The biphasic layers were separated, and the organic layer was washed with brine (2 × 10 mL) and dried (Na_2_SO_4_). The solvent was removed under reduced pressure to provide the mixture of crude aldehydes, the majority of which was the β aldehyde epimer (matching *R*_*f*_ and ^1^H NMR) similar to the enantiomer synthesized earlier.^34,36,43^ The addition of EtOAc on the sides of the flask resulted in precipitation of insoluble solids (sulfur impurities), which were removed by filtration through a filter paper. The solvent (EtOAc) was removed under reduced pressure. This process was repeated 4 to 5 times until there was no longer precipitation of sulfur byproducts.

Analysis of the mixture by ^1^H NMR indicated the presence of the majority of β aldehydes (peak at 10.01 ppm). The epimerization into the more stable α-aldehyde **29** was achieved by following the procedure of Chitra Edwankar et al.^33,42^ To the solution of the crude mixture (aldehyde epimers) in a solution of MeOH (30 mL) was added triethylamine (8.5 mL) with stirring, and the mixture was stirred for 14 h at rt to permit complete epimerization to the α-aldehyde **29** (peak at 9.85 ppm in ^1^H NMR). The methanol was then removed under reduced pressure to obtain a crude oil, which was purified by flash column chromatography (basic alumina, 2% MeOH in CH_2_Cl_2_) to afford the α-aldehyde **29** as a sole product and a present as colorless oil (348 mg, 70%): **[α]**_**D**_^25^ - 125.24 (c 1.03, CHCl3); ^**1**^ **H NMR** (500 MHz, CDCl_3_) δ 9.85 (s, 1H), 7.50 (d, *J* = 7.7 Hz, 1H), 7.30 (d, *J* = 8.2 Hz, 1H), 7.23–7.16 (m, 1H), 7.14–7.08 (m, 1H), 4.20 (d, *J* = 7.9 Hz, 1H), 3.87–3.77 (m, 2H), 3.63 (s, 3H), 3.46 (dq, *J* = 13.6, 6.9 Hz, 1H), 3.25–3.20 (m, 1H), 3.00 (dd, *J* = 15.3, 5.1 Hz, 1H), 2.74 (d, *J* = 14.3 Hz, 1H), 2.53 (d, *J* = 8.5 Hz, 1H), 2.46 (d, *J* = 2.0 Hz, 1H), 1.92 (ddd, *J* = 11.8, 9.8, 1.7 Hz, 1H), 1.81–1.74 (m, 1H), 1.45–1.39 (m, 1H), 1.36 (d, *J* = 6.8 Hz, 3H), 1.10 (d, *J* = 5.9 Hz, 21H); ^**13**^ **C NMR** (126 MHz, CDCl_3_) δ 203.6 (CH), 139.2 (C), 137.3 (C), 127.4 (C), 120.9 (CH), 118.8 (CH), 118.0 (CH), 108.7 (CH), 104.3 (C), 64.3 (CH2), 52.0 (CH), 51.4 (CH), 51.1 (CH), 47.7 (CH), 42.2 (CH), 29.6 (CH2), 29.3 (CH_3_), 27.0 (CH_2_), 26.7 (CH), 19.4 (CH_3_), 18.1 (CH_3_X6), 11.9 (CHX6); **DEPT 135** ^**13**^ **C NMR** (126 MHz, CDCl_3_) δ 120.8 (CH), 118.8 (CH), 118.0 (CH), 108.7 (CH), 64.3 (CH2), 52.0 (CH), 51.4 (CH), 51.1 (CH), 47.7 (CH), 42.2 (CH), 29.5 (CH_2_), 29.3 (CH_3_), 27.0 (CH_2_), 26.7 (CH), 19.4 (CH3), 18.1 (6XCH_3_), 11.9 (6 x CH); **HRMS** (ESI) *m*/*z* (M + H)^+^ calcd for C_29_H_44_N_2_O_2_Si, 481.3244, found 481.3251. The NMR spectroscopic data of the aldehyde **29** were in excellent agreement with that reported in the literature for its enantiomer.^36,41^

### Quaternization of 29 into (6*R*,7*S*,8*R*,9*R*,10*S*,11*S*,11a*R*)-9-Formyl-5,7,8-trimethyl-11-(((triisopropylsilyl)­oxy) methyl)-6,7,8,9,10,11,11a,12-octahydro-5*H*-6,10-methanoindolo­[3,2-*b*]­quinolizin-7-ium iodide (30)

Iodomethane (excess, 0.5 mL) was added to a solution of aldehyde **29** (69 mg, 0.14 mmol) in methanol (3 mL) at 0 °C with stirring, after which the mixture was allowed to stir in the dark (the flask was covered with Al foil) at 0 °C for 16 h. The analysis by TLC (basic alumina, 0.1 mL of MeOH in 2.8 mL of CH_2_Cl_2_) indicated the completion of the reaction with a more polar component. At this point, the solvent was removed under high vacuum for 7 h to provide the *N*_b_-methyl salt **30** (64 mg, 92%): *R*_*f*_ 0.5 (basic alumina, CH_2_Cl_2_/MeOH, 2.4:0.1; LCMS 495 (base peak of product desired, water and acetonitrile were used as the mobile phase); HRMS (ESI) *m*/*z* (M)^+^ calcd for C_30_H_46_N_2_O_2_Si, 495.3401, found 495.3435. The salt was used in the next step without further purification.

To the solution of the *N*_b_-methyl salt **30** (10 mg, 0.12 mmol) in dry THF (>99.9% pure purchased from Sigma-Aldrich, 2.5 mL) was added 1.0 M of NaHMDS in THF (44.4 mg, 0.36 mmol), with stirring, at 0 °C. The color (light yellow) of the solution turned to a light green. The reaction mixture which resulted was allowed to warm to rt and then was stirred for 16 h at rt. After the completion of the reaction as indicated by TLC analysis, THF was removed under vacuum at reduced temperature to yield compound **31** in 41% crude yield; HRMS (ESI) *m*/*z* (M + H)^+^ calcd for C_30_H_46_N_2_O_2_Si (**31**) 495.3401, found 495.3439. The product was used in the next step without further characterization and purification.

### 2-((6*R*,9*S*,10*R*)-5,12-Dimethyl-9-(((triisopropylsilyl)­oxy) methyl)-6,7,8,9,10,11-hexahydro-5*H*-6,10-epiminocycloocta­[b]­indol-8-yl)­but-2-enal (31) into (+)-Talcarpine (1) and (−)-*N*_4_-Methyl,*N*_4_-21-*seco*talcarpine (2)

To the mixture of crude unsaturated aldehyde **31** (6 mg crude) in THF (5 mL) 0.1 *N* aq HCl (5 mL) was added, and the mixture which resulted was heated to reflux and stirred for 6 h. Analysis by TLC (silica gel, 2–3% MeOH in CH_2_Cl_2_ and a few drops of 10% aq NH_4_OH) indicated the disappearance of starting material **31** and the formation of two new components: an upper strong colored component and a lower faint colored component (under UV). When the TLC plate was dipped into ceric ammonium nitrate solution in phosphoric acid (CAN) stain, the color (dark blue) indicated the presence of two indole components (**1** and **2**), the *R*_*f*_ values of which matched with those of the authentic samples (enantiomers of **1** and **2**).^34,41^ Analysis by LCMS indicated the base peak of 339 amu for the top (*R*_*f*_) component (base peak in H_2_O and 0.1% formic acid/acetonitrile mobile phase). The reaction mixture was cooled to rt, and the solvents were reduced to 1/3 of their original volume by removal under reduced pressure. The crude mixture which resulted was brought to pH 8 by adding 10% aq NH_4_OH dropwise at 0 °C. The biphasic layers were separated, and the aq layer was extracted back with CH_2_Cl_2_ (3 × 10 mL). The combined organic layers which resulted were washed with brine (15 mL) and dried (Na_2_SO_4_). The crude material was purified with flash column chromatography (silica gel, 2–3% MeOH in CH_2_Cl_2_ and aq NH_4_OH in a Pasteur pipet) to furnish the (+)-talcarpine **1** (crude yield: 68%): **[α]**_**D**_^**25**^ + 36.5­(c 0.08, CHCl_3_); reference value for (−)-talcarpine **1**: [α]_D_^25^ – 30 (c 0.1, CHCl_3_);^34^ ^**1**^ **H NMR** ^1^H NMR (500 MHz, CDCl_3_) δ 9.95 (s, 1H), 7.48 (d, *J* = 7.8 Hz, 1H), 7.28 (d, *J* = 8.4 Hz, 1H), 7.19 (t, *J* = 7.4 Hz, 1H), 7.10 (t, *J* = 7.0 Hz, 1H), 4.14 (t, *J* = 11.5 Hz, 1H), 3.98 (s, 2H), 3.89 (dd, *J* = 11.3, 4.8 Hz, 1H), 3.62 (s, 3H), 3.27 (dd, *J* = 16.7, 6.7 Hz, 1H), 2.91 (s, 1H), 2.47 (m, *J* = 14.2 Hz, 2H), 2.32 (s, 3H), 2.20 (d, *J* = 13.0 Hz, 1H), 2.05 (s, 1H), 1.79 (s, 1H), 1.45 (d, *J* = 13.2 Hz, 1H), 1.30 (d, *J* = 6.6 Hz, 3H); ^**13**^ **C NMR** (126 MHz, CDCl_3_) δ 204.7, 137.0, 132.6, 126.4, 121.0, 118.9, 118.2, 108.8, 106.6, 69.5, 68.8, 54.5, 54.4, 53.5, 41.7, 39.4, 28.9 (s), 28.9, 26.9, 22.5, 19.2; **HRMS** (ESI) *m*/*z* (M + H)^+^ calcd for C_21_H_26_N_2_ O_2_ 339.2067, measured 339.2075. The NMR spectroscopic data (^1^H and ^13^C NMR) and the optical rotation (within experimental error) of this material were in excellent agreement with that reported in the literature for its enantiomer.^34,41,45^

The lower faint indole component, which appeared on TLC also exhibited an LCMS of 339 amu. The analysis on HRMS: (ESI) *m*/*z* (M + H)^+^ calcd for C_21_H_26_N_2_O_2_, predicted 339.2067, measured 339.2074 and had the same *R*_*f*_ value, when compared to the *R*_*f*_ value of the natural (+)-*N*_4_-methyl,*N*_4_-21-*seco*talpinine **2**.^34,41^ Thus, the minor product obtained in this reaction is the desired unnatural (−)-*N*_4_-methyl,*N*_4_-21-*seco*talpinine **2** indole alkaloid (the indole **2** was not enough to characterize further).

## Supplementary Material







## Notes

X-ray crystallographic data (ZIP)
NMR data (ZIP)
LCMS and HRMS data (PDF)
