# Supplementary material for: First Total Synthesis of the Unnatural (+)-Talcarpine and (−)‑N 4‑Methyl,N 4‑21-secotalpinine
Source: ACS Omega. 2026 Apr 29;11(18):26942–56. doi: 10.1021/acsomega.5c13509 (PMC13176970; doi:10.1021/acsomega.5c13509)
Supplement: Supplementary file 1 [file ao5c13509_si_001.zip › X-ray data for review/Cook 185 (compound 19)/cook185_tables.rtf]

Table 1. Crystal data and structure refinement for KPP-II-43.
Identification code 	cook185
Empirical formula 	C19H20N2O
Formula weight 	292.37
Temperature 	296(2) K
Wavelength 	0.71073 Å
Crystal system 	Monoclinic
Space group 	P21
Unit cell dimensions	a = 8.7184(3) Å	a= 90°.
	b = 8.4717(3) Å	b= 92.9370(10)°.
	c = 10.3859(3) Å	g = 90°.
Volume	766.09(4) Å3
Z	2
Density (20°C)	1.267 Mg/m3
Absorption coefficient	0.079 mm-1
F(000)	312
Crystal size	0.452 x 0.355 x 0.272 mm3
Theta range for data collection	1.963 to 29.151°.
Index ranges	-10<=h<=11, -11<=k<=11, -14<=l<=14
Reflections collected	8923
Independent reflections	4066 [Rint = 0.0142]
Completeness to theta = 25.242°	99.2 % 
Refinement method	Full-matrix least-squares on F2
Data / restraints / parameters	4066 / 1 / 201
Goodness-of-fit on F2	0.916
Final R indices [I>2sigma(I)]	R1 = 0.0340, wR2 = 0.0954
R indices (all data)	R1 = 0.0368, wR2 = 0.0992
Absolute structure parameter	0.4(3)
Largest diff. peak and hole	0.212 and -0.162 e.Å-3

Table 2. Atomic coordinates (x 104) and equivalent isotropic displacement parameters (Å2x 103)
for KPP-II-43. U(eq) is defined as one third of the trace of the orthogonalized Uij tensor.
________________________________________________________________________________
	x	y	z	U(eq)
________________________________________________________________________________
C(1)	634(2)	6863(2)	11114(2)	45(1)
C(2)	-20(2)	6638(2)	10100(2)	36(1)
C(3)	-826(2)	6373(2)	8827(2)	34(1)
C(3A)	-2332(2)	7291(2)	8738(2)	43(1)
N(4)	98(1)	6754(1)	7712(1)	32(1)
C(5)	1029(2)	8200(2)	7870(2)	34(1)
C(6)	2561(2)	7972(2)	8644(2)	39(1)
O(6)	3189(2)	9094(2)	9167(2)	61(1)
C(7)	3334(2)	6369(2)	8639(2)	46(1)
C(8)	2288(2)	4963(2)	8314(2)	38(1)
C(9)	1044(2)	5427(2)	7284(1)	33(1)
C(10)	1755(2)	5974(2)	6074(2)	33(1)
N(11)	2494(2)	4992(2)	5236(1)	36(1)
C(11)	2513(2)	3280(2)	5231(2)	44(1)
C(12)	3106(2)	5934(2)	4301(1)	36(1)
C(13)	3982(2)	5519(2)	3263(2)	43(1)
C(14)	4420(2)	6713(3)	2454(2)	48(1)
C(15)	4007(2)	8286(3)	2664(2)	48(1)
C(16)	3180(2)	8712(2)	3716(2)	42(1)
C(17)	2728(2)	7526(2)	4559(2)	35(1)
C(18)	1890(2)	7520(2)	5714(2)	34(1)
C(19)	1345(2)	8851(2)	6523(2)	38(1)
________________________________________________________________________________
Table 3. Bond lengths [Å] and angles [°] for KPP-II-43.
_____________________________________________________
C(1)-C(2) 	1.187(3)	C(1)-H(1) 	0.9300
C(2)-C(3) 	1.482(2)	C(3)-N(4) 	1.4804(19)
C(3)-C(3A) 	1.524(2)	C(3)-H(3A) 	0.9800
C(3A)-H(3D) 	0.9600	C(3A)-H(3E) 	0.9600
C(3A)-H(3F) 	0.9600	N(4)-C(5) 	1.4739(18)
N(4)-C(9) 	1.4767(19)	C(5)-C(6) 	1.535(2)
C(5)-C(19) 	1.543(2)	C(5)-H(5A) 	0.9800
C(6)-O(6) 	1.211(2)	C(6)-C(7) 	1.516(3)
C(7)-C(8) 	1.527(3)	C(7)-H(7A) 	0.9700
C(7)-H(7B) 	0.9700	C(8)-C(9) 	1.535(2)
C(8)-H(8A) 	0.9700	C(8)-H(8B) 	0.9700
C(9)-C(10) 	1.503(2)	C(9)-H(9A) 	0.9800
C(10)-C(18) 	1.369(2)	C(10)-N(11) 	1.386(2)
N(11)-C(12) 	1.385(2)	N(11)-C(11) 	1.451(2)
C(11)-H(11A) 	0.9600	C(11)-H(11B) 	0.9600
C(11)-H(11C) 	0.9600	C(12)-C(13) 	1.398(2)
C(12)-C(17) 	1.417(2)	C(13)-C(14) 	1.381(3)
C(13)-H(13A) 	0.9300	C(14)-C(15) 	1.400(3)
C(14)-H(14A) 	0.9300	C(15)-C(16) 	1.387(3)
C(15)-H(15A) 	0.9300	C(16)-C(17) 	1.403(2)
C(16)-H(16A) 	0.9300	C(17)-C(18) 	1.435(2)
C(18)-C(19) 	1.498(2)	C(19)-H(19C) 	0.9700
C(19)-H(19A) 	0.9700

C(2)-C(1)-H(1)	180.0	C(1)-C(2)-C(3)	179.33(19)
N(4)-C(3)-C(2)	114.36(12)	N(4)-C(3)-C(3A)	110.00(12)
C(2)-C(3)-C(3A)	110.18(13)	N(4)-C(3)-H(3A)	107.3
C(2)-C(3)-H(3A)	107.3	C(3A)-C(3)-H(3A)	107.3
C(3)-C(3A)-H(3D)	109.5	C(3)-C(3A)-H(3E)	109.5
H(3D)-C(3A)-H(3E)	109.5	C(3)-C(3A)-H(3F)	109.5
H(3D)-C(3A)-H(3F)	109.5	H(3E)-C(3A)-H(3F)	109.5
C(5)-N(4)-C(9)	110.68(11)	C(5)-N(4)-C(3)	114.38(12)
C(9)-N(4)-C(3)	113.83(11)	N(4)-C(5)-C(6)	114.46(12)
N(4)-C(5)-C(19)	108.61(12)	C(6)-C(5)-C(19)	109.20(13)
N(4)-C(5)-H(5A)	108.1	C(6)-C(5)-H(5A)	108.1
C(19)-C(5)-H(5A)	108.1	O(6)-C(6)-C(7)	120.92(17)
O(6)-C(6)-C(5)	119.84(18)	C(7)-C(6)-C(5)	119.01(13)
C(6)-C(7)-C(8)	116.06(14)	C(6)-C(7)-H(7A)	108.3
C(8)-C(7)-H(7A)	108.3	C(6)-C(7)-H(7B)	108.3
C(8)-C(7)-H(7B)	108.3	H(7A)-C(7)-H(7B)	107.4
C(7)-C(8)-C(9)	110.29(13)	C(7)-C(8)-H(8A)	109.6
C(9)-C(8)-H(8A)	109.6	C(7)-C(8)-H(8B)	109.6
C(9)-C(8)-H(8B)	109.6	H(8A)-C(8)-H(8B)	108.1
N(4)-C(9)-C(10)	106.09(12)	N(4)-C(9)-C(8)	111.79(12)
Table 3. (continued).
_____________________________________________________

C(10)-C(9)-C(8)	110.78(13)	N(4)-C(9)-H(9A)	109.4
C(10)-C(9)-H(9A)	109.4	C(8)-C(9)-H(9A)	109.4
C(18)-C(10)-N(11)	110.66(14)	C(18)-C(10)-C(9)	124.69(13)
N(11)-C(10)-C(9)	124.39(13)	C(12)-N(11)-C(10)	107.63(13)
C(12)-N(11)-C(11)	124.67(15)	C(10)-N(11)-C(11)	127.44(15)
N(11)-C(11)-H(11A)	109.5	N(11)-C(11)-H(11B)	109.5
H(11A)-C(11)-H(11B)	109.5	N(11)-C(11)-H(11C)	109.5
H(11A)-C(11)-H(11C)	109.5	H(11B)-C(11)-H(11C)	109.5
N(11)-C(12)-C(13)	129.90(16)	N(11)-C(12)-C(17)	108.30(14)
C(13)-C(12)-C(17)	121.79(15)	C(14)-C(13)-C(12)	117.65(18)
C(14)-C(13)-H(13A)	121.2	C(12)-C(13)-H(13A)	121.2
C(13)-C(14)-C(15)	121.40(17)	C(13)-C(14)-H(14A)	119.3
C(15)-C(14)-H(14A)	119.3	C(16)-C(15)-C(14)	121.21(17)
C(16)-C(15)-H(15A)	119.4	C(14)-C(15)-H(15A)	119.4
C(15)-C(16)-C(17)	118.65(17)	C(15)-C(16)-H(16A)	120.7
C(17)-C(16)-H(16A)	120.7	C(16)-C(17)-C(12)	119.21(15)
C(16)-C(17)-C(18)	134.04(15)	C(12)-C(17)-C(18)	106.75(13)
C(10)-C(18)-C(17)	106.63(13)	C(10)-C(18)-C(19)	122.17(14)
C(17)-C(18)-C(19)	130.96(14)	C(18)-C(19)-C(5)	108.42(12)
C(18)-C(19)-H(19C)	110.0	C(5)-C(19)-H(19C)	110.0
C(18)-C(19)-H(19A)	110.0	C(5)-C(19)-H(19A)	110.0
H(19C)-C(19)-H(19A)	108.4
_____________________________________________________________

 

Table 4. Anisotropic displacement parameters (Å2x 103) for KPP-II-43. The anisotropic
displacement factor exponent takes the form: -2p2[h2a*2U11 + ... + 2 h k a* b* U12]
______________________________________________________________________________
	U11	U22	U33	U23	U13	U12
______________________________________________________________________________
C(1)	45(1) 	42(1)	46(1) 	1(1)	-5(1) 	-2(1)
C(2)	32(1) 	34(1)	42(1) 	5(1)	4(1) 	0(1)
C(3)	30(1) 	32(1)	40(1) 	2(1)	2(1) 	-2(1)
C(3A)	31(1) 	51(1)	47(1) 	-1(1)	1(1) 	4(1)
N(4)	31(1) 	25(1)	39(1) 	0(1)	3(1) 	-1(1)
C(5)	36(1) 	26(1)	40(1) 	-3(1)	5(1) 	-2(1)
C(6)	36(1) 	41(1)	41(1) 	-3(1)	3(1) 	-9(1)
O(6)	59(1) 	54(1)	70(1) 	-12(1)	-7(1) 	-20(1)
C(7)	34(1) 	52(1)	51(1) 	-3(1)	-6(1) 	2(1)
C(8)	39(1) 	35(1)	41(1) 	4(1)	3(1) 	8(1)
C(9)	36(1) 	24(1)	38(1) 	0(1)	2(1) 	-1(1)
C(10)	37(1) 	27(1)	35(1) 	-2(1)	1(1) 	1(1)
N(11)	44(1) 	27(1)	37(1) 	-3(1)	3(1) 	2(1)
C(11)	56(1) 	27(1)	49(1) 	-6(1)	-3(1) 	5(1)
C(12)	36(1) 	36(1)	34(1) 	-3(1)	-1(1) 	-2(1)
C(13)	39(1) 	50(1)	40(1) 	-11(1)	0(1) 	0(1)
C(14)	38(1) 	67(1)	37(1) 	-5(1)	3(1) 	-7(1)
C(15)	41(1) 	60(1)	41(1) 	7(1)	3(1) 	-12(1)
C(16)	43(1) 	40(1)	43(1) 	5(1)	1(1) 	-7(1)
C(17)	35(1) 	35(1)	35(1) 	-1(1)	-1(1) 	-2(1)
C(18)	39(1) 	27(1)	37(1) 	1(1)	4(1) 	0(1)
C(19)	45(1) 	24(1)	45(1) 	1(1)	7(1) 	1(1)
______________________________________________________________________________
Table 5. Hydrogen coordinates (x 104) and isotropic displacement parameters (Å2x 103) for KPP-II-43.
________________________________________________________________________________
	x 	y 	z 	U(eq)
________________________________________________________________________________

H(1)	1146	7040	11909	53
H(3A)	-1085	5248	8771	41
H(3D)	-2895	7025	7949	64
H(3E)	-2117	8402	8751	64
H(3F)	-2931	7024	9456	64
H(5A)	426	8986	8317	40
H(7A)	3832	6192	9483	55
H(7B)	4131	6398	8021	55
H(8A)	1808	4610	9085	46
H(8B)	2893	4097	7998	46
H(9A)	382	4514	7086	39
H(11A)	3555	2915	5329	66
H(11B)	2055	2901	4430	66
H(11C)	1942	2892	5931	66
H(13A)	4260	4476	3122	52
H(14A)	5003	6467	1756	57
H(15A)	4291	9059	2087	57
H(16A)	2931	9763	3859	51
H(19C)	414	9304	6126	45
H(19A)	2122	9671	6595	45
________________________________________________________________________________
Table 6. Torsion angles [°] for KPP-II-43.
________________________________________________________________
C(2)-C(3)-N(4)-C(5)	-40.66(18)	C(3A)-C(3)-N(4)-C(5)	83.94(16)
C(2)-C(3)-N(4)-C(9)	87.94(16)	C(3A)-C(3)-N(4)-C(9)	-147.46(14)
C(9)-N(4)-C(5)-C(6)	-48.11(17)	C(3)-N(4)-C(5)-C(6)	82.07(16)
C(9)-N(4)-C(5)-C(19)	74.20(15)	C(3)-N(4)-C(5)-C(19)	-155.63(12)
N(4)-C(5)-C(6)-O(6)	-157.51(16)	C(19)-C(5)-C(6)-O(6)	80.51(19)
N(4)-C(5)-C(6)-C(7)	28.0(2)	C(19)-C(5)-C(6)-C(7)	-94.00(17)
O(6)-C(6)-C(7)-C(8)	162.78(17)	C(5)-C(6)-C(7)-C(8)	-22.8(2)
C(6)-C(7)-C(8)-C(9)	36.5(2)	C(5)-N(4)-C(9)-C(10)	-55.49(16)
C(3)-N(4)-C(9)-C(10)	174.05(12)	C(5)-N(4)-C(9)-C(8)	65.37(16)
C(3)-N(4)-C(9)-C(8)	-65.09(16)	C(7)-C(8)-C(9)-N(4)	-58.67(17)
C(7)-C(8)-C(9)-C(10)	59.43(17)	N(4)-C(9)-C(10)-C(18)	19.1(2)
C(8)-C(9)-C(10)-C(18)	-102.44(19)	N(4)-C(9)-C(10)-N(11)	-167.46(14)
C(8)-C(9)-C(10)-N(11)	71.02(19)	C(18)-C(10)-N(11)-C(12)	-0.70(19)
C(9)-C(10)-N(11)-C(12)	-174.96(14)	C(18)-C(10)-N(11)-C(11)	-174.96(16)
C(9)-C(10)-N(11)-C(11)	10.8(3)	C(10)-N(11)-C(12)-C(13)	178.33(16)
C(11)-N(11)-C(12)-C(13)	-7.2(3)	C(10)-N(11)-C(12)-C(17)	-0.54(18)
C(11)-N(11)-C(12)-C(17)	173.91(16)	N(11)-C(12)-C(13)-C(14)	178.63(17)
C(17)-C(12)-C(13)-C(14)	-2.6(2)	C(12)-C(13)-C(14)-C(15)	0.1(3)
C(13)-C(14)-C(15)-C(16)	2.0(3)	C(14)-C(15)-C(16)-C(17)	-1.6(3)
C(15)-C(16)-C(17)-C(12)	-0.9(2)	C(15)-C(16)-C(17)-C(18)	179.82(18)
N(11)-C(12)-C(17)-C(16)	-177.92(14)	C(13)-C(12)-C(17)-C(16)	3.1(2)
N(11)-C(12)-C(17)-C(18)	1.50(18)	C(13)-C(12)-C(17)-C(18)	-177.47(15)
N(11)-C(10)-C(18)-C(17)	1.63(19)	C(9)-C(10)-C(18)-C(17)	175.86(14)
N(11)-C(10)-C(18)-C(19)	-173.45(15)	C(9)-C(10)-C(18)-C(19)	0.8(3)
C(16)-C(17)-C(18)-C(10)	177.41(18)	C(12)-C(17)-C(18)-C(10)	-1.90(18)
C(16)-C(17)-C(18)-C(19)	-8.1(3)	C(12)-C(17)-C(18)-C(19)	172.58(17)
C(10)-C(18)-C(19)-C(5)	13.5(2)	C(17)-C(18)-C(19)-C(5)	-160.21(16)
N(4)-C(5)-C(19)-C(18)	-48.15(17)	C(6)-C(5)-C(19)-C(18)	77.29(16)	
________________________________________________________________
