# Supplementary material for: First Total Synthesis of the Unnatural (+)-Talcarpine and (−)‑N 4‑Methyl,N 4‑21-secotalpinine
Source: ACS Omega. 2026 Apr 29;11(18):26942–56. doi: 10.1021/acsomega.5c13509 (PMC13176970; doi:10.1021/acsomega.5c13509)
Supplement: Supplementary file 1 [file ao5c13509_si_001.zip › X-ray data for review/cook 196 (compound 11)/cook196_tables.rtf]

Table 1. Crystal data and structure refinement for KPP-II-50.
Identification code 	cook196
Empirical formula 	C19H20N2O
Formula weight 	292.37
Temperature 	293(2) K
Wavelength 		1.54178 Å
Crystal system 	Monoclinic
Space group 	P21
Unit cell dimensions	a = 10.3448(2) Å	a= 90°.
	b = 7.8977(2) Å	b= 117.6662(4)°.
	c = 10.7674(2) Å	g = 90°.
Volume	779.12(3) Å3
Z	2
Density (calculated)	1.246 Mg/m3
Absorption coefficient	0.609 mm-1
F(000)	312
Crystal size	0.330 x 0.270 x 0.088 mm3
Theta range for data collection	4.637 to 74.631°.
Index ranges	-12<=h<=12, -9<=k<=9, -13<=l<=13
Reflections collected	17046
Independent reflections	3160 [Rint = 0.0234]
Completeness to theta = 67.679°	99.1 % 
Refinement method	Full-matrix least-squares on F2
Data / restraints / parameters	3160 / 1 / 201
Goodness-of-fit on F2	1.022
Final R indices [I>2sigma(I)]	R1 = 0.0300, wR2 = 0.0830
R indices (all data)	R1 = 0.0301, wR2 = 0.0831
Absolute structure parameter	0.07(4)	
Largest diff. peak and hole	0.156 and -0.117 e.Å-3

Table 2. Atomic coordinates (x 104) and equivalent isotropic displacement parameters (Å2x 103) for KPP-II-50. U(eq) is defined as one third of the trace of the orthogonalized Uij tensor.
________________________________________________________________________________
	x	y	z	U(eq)
________________________________________________________________________________
N(1)	8010(2)	4675(2)	6899(2)	46(1)
C(2)	6675(2)	4951(2)	6856(2)	38(1)
C(3)	5442(2)	3716(2)	6239(2)	36(1)
N(4)	4096(1)	4609(2)	6031(1)	36(1)
C(5)	4368(2)	5411(2)	7377(2)	39(1)
C(6)	5403(2)	6925(2)	7721(2)	45(1)
C(7)	6699(2)	6404(2)	7547(2)	41(1)
C(8)	8136(2)	7099(2)	8059(2)	47(1)
C(9)	8856(3)	8533(3)	8854(2)	63(1)
C(10)	10314(3)	8773(3)	9193(2)	79(1)
C(11)	11068(3)	7634(4)	8782(2)	76(1)
C(12)	10398(2)	6224(4)	8008(2)	63(1)
C(13)	8926(2)	5984(2)	7644(2)	48(1)
C(14)	5754(2)	2151(2)	7222(2)	42(1)
C(15)	4780(2)	2294(2)	7951(2)	42(1)
C(16)	4941(2)	4066(2)	8539(2)	41(1)
O(17)	5422(2)	4384(2)	9769(1)	62(1)
C(18)	1411(2)	4133(3)	4927(2)	55(1)
C(19)	2915(2)	3322(2)	5599(2)	41(1)
C(20)	3203(2)	2173(2)	6833(2)	45(1)
C(21)	2243(3)	1208(4)	6970(3)	77(1)
C(22)	8422(3)	3212(3)	6351(3)	70(1)
________________________________________________________________________________
Table 3. Bond lengths [Å] and angles [°] for KPP-II-50.
_____________________________________________________
N(1)-C(2) 	1.378(2)	N(1)-C(13) 	1.380(2)
N(1)-C(22) 	1.449(3)	C(2)-C(7) 	1.361(2)
C(2)-C(3) 	1.494(2)	C(3)-N(4) 	1.482(2)
C(3)-C(14) 	1.561(2)	C(3)-H(3) 	0.9800
N(4)-C(5) 	1.4842(19)	N(4)-C(19) 	1.488(2)
C(5)-C(6) 	1.531(2)	C(5)-C(16) 	1.535(2)
C(5)-H(5) 	0.9800	C(6)-C(7) 	1.496(2)
C(6)-H(6A) 	0.9700	C(6)-H(6B) 	0.9700
C(7)-C(8) 	1.433(2)	C(8)-C(9) 	1.406(3)
C(8)-C(13) 	1.407(3)	C(9)-C(10) 	1.389(4)
C(9)-H(9) 	0.9300	C(10)-C(11) 	1.391(4)
C(10)-H(10) 	0.9300	C(11)-C(12) 	1.371(4)
C(11)-H(11) 	0.9300	C(12)-C(13) 	1.398(3)
C(12)-H(12) 	0.9300	C(14)-C(15) 	1.541(2)
C(14)-H(14A) 	0.9700	C(14)-H(14B) 	0.9700
C(15)-C(16) 	1.513(2)	C(15)-C(20) 	1.515(2)
C(15)-H(15) 	0.9800	C(16)-O(17) 	1.205(2)
C(18)-C(19) 	1.519(2)	C(18)-H(18A) 	0.9600
C(18)-H(18B) 	0.9600	C(18)-H(18C) 	0.9600
C(19)-C(20) 	1.520(2)	C(19)-H(19) 	0.9800
C(20)-C(21) 	1.314(3)	C(21)-H(21A) 	0.9300
C(21)-H(21B) 	0.9300	C(22)-H(22A) 	0.9600
C(22)-H(22B) 	0.9600	C(22)-H(22C) 	0.9600

C(2)-N(1)-C(13)	107.93(15)	C(2)-N(1)-C(22)	126.33(15)
C(13)-N(1)-C(22)	125.61(16)	C(7)-C(2)-N(1)	110.57(14)
C(7)-C(2)-C(3)	126.05(14)	N(1)-C(2)-C(3)	123.16(14)
N(4)-C(3)-C(2)	107.74(12)	N(4)-C(3)-C(14)	111.29(12)
C(2)-C(3)-C(14)	110.50(13)	N(4)-C(3)-H(3)	109.1
C(2)-C(3)-H(3)	109.1	C(14)-C(3)-H(3)	109.1
C(3)-N(4)-C(5)	108.47(11)	C(3)-N(4)-C(19)	107.44(12)
C(5)-N(4)-C(19)	110.63(12)	N(4)-C(5)-C(6)	111.31(13)
N(4)-C(5)-C(16)	109.10(13)	C(6)-C(5)-C(16)	112.43(13)
N(4)-C(5)-H(5)	107.9	C(6)-C(5)-H(5)	107.9
C(16)-C(5)-H(5)	107.9	C(7)-C(6)-C(5)	108.77(13)
C(7)-C(6)-H(6A)	109.9	C(5)-C(6)-H(6A)	109.9
C(7)-C(6)-H(6B)	109.9	C(5)-C(6)-H(6B)	109.9
H(6A)-C(6)-H(6B)	108.3	C(2)-C(7)-C(8)	106.59(15)
C(2)-C(7)-C(6)	120.68(15)	C(8)-C(7)-C(6)	132.61(16)
C(9)-C(8)-C(13)	118.40(19)	C(9)-C(8)-C(7)	134.8(2)
C(13)-C(8)-C(7)	106.83(15)	C(10)-C(9)-C(8)	118.2(2)
C(10)-C(9)-H(9)	120.9	C(8)-C(9)-H(9)	120.9
C(9)-C(10)-C(11)	121.9(2)	C(9)-C(10)-H(10)	119.0
C(11)-C(10)-H(10)	119.0	C(12)-C(11)-C(10)	121.2(2)
Table 3. (continued).
_____________________________________________________

C(12)-C(11)-H(11)	119.4	C(10)-C(11)-H(11)	119.4
C(11)-C(12)-C(13)	117.3(3)	C(11)-C(12)-H(12)	121.4
C(13)-C(12)-H(12)	121.4	N(1)-C(13)-C(12)	129.0(2)
N(1)-C(13)-C(8)	108.08(15)	C(12)-C(13)-C(8)	122.92(19)
C(15)-C(14)-C(3)	108.00(12)	C(15)-C(14)-H(14A)	110.1
C(3)-C(14)-H(14A)	110.1	C(15)-C(14)-H(14B)	110.1
C(3)-C(14)-H(14B)	110.1	H(14A)-C(14)-H(14B)	108.4
C(16)-C(15)-C(20)	105.03(14)	C(16)-C(15)-C(14)	108.07(13)
C(20)-C(15)-C(14)	107.95(14)	C(16)-C(15)-H(15)	111.8
C(20)-C(15)-H(15)	111.8	C(14)-C(15)-H(15)	111.8
O(17)-C(16)-C(15)	124.21(16)	O(17)-C(16)-C(5)	123.86(16)
C(15)-C(16)-C(5)	111.90(13)	C(19)-C(18)-H(18A)	109.5
C(19)-C(18)-H(18B)	109.5	H(18A)-C(18)-H(18B)	109.5
C(19)-C(18)-H(18C)	109.5	H(18A)-C(18)-H(18C)	109.5
H(18B)-C(18)-H(18C)	109.5	N(4)-C(19)-C(18)	111.82(15)
N(4)-C(19)-C(20)	109.59(12)	C(18)-C(19)-C(20)	114.20(15)
N(4)-C(19)-H(19)	106.9	C(18)-C(19)-H(19)	106.9
C(20)-C(19)-H(19)	106.9	C(21)-C(20)-C(15)	122.61(18	
C(21)-C(20)-C(19)	126.37(18)	C(15)-C(20)-C(19)	111.01(13)
C(20)-C(21)-H(21A)	120.0	C(20)-C(21)-H(21B)	120.0
H(21A)-C(21)-H(21B)	120.0	N(1)-C(22)-H(22A)	109.5
N(1)-C(22)-H(22B)	109.5	H(22A)-C(22)-H(22B)	109.5
N(1)-C(22)-H(22C)	109.5	H(22A)-C(22)-H(22C)	109.5
H(22B)-C(22)-H(22C)	109.5
_____________________________________________________________

Table 4. Anisotropic displacement parameters (Å2x 103) for KPP-II-50. The anisotropic
displacement factor exponent takes the form: -2p2[h2a*2U11 + ... + 2 h k a* b* U12]
______________________________________________________________________________
	U11	U22	U33	U23	U13	U12
______________________________________________________________________________
N(1)	43(1) 	48(1)	54(1) 	-9(1)	29(1) 	-9(1)
C(2)	43(1) 	36(1)	41(1) 	-4(1)	23(1) 	-6(1)
C(3)	40(1) 	36(1)	36(1) 	-7(1)	21(1) 	-5(1)
N(4)	38(1) 	38(1)	32(1) 	0(1)	16(1) 	0(1)
C(5)	42(1) 	40(1)	37(1) 	-2(1)	21(1) 	4(1)
C(6)	57(1) 	36(1)	44(1) 	-6(1)	26(1) 	1(1)
C(7)	50(1) 	35(1)	38(1) 	-4(1)	21(1) 	-7(1)
C(8)	57(1) 	44(1)	36(1) 	0(1)	18(1) 	-15(1)
C(9)	83(1) 	52(1)	45(1) 	-9(1)	22(1) 	-26(1)
C(10)	85(2) 	80(2)	49(1) 	-9(1)	12(1) 	-48(1)
C(11)	60(1) 	103(2)	51(1) 	3(1)	14(1) 	-39(1)
C(12)	49(1) 	85(2)	53(1) 	6(1)	21(1) 	-17(1)
C(13)	47(1) 	54(1)	41(1) 	1(1)	19(1) 	-13(1)
C(14)	41(1) 	33(1)	52(1) 	-1(1)	21(1) 	0(1)
C(15)	42(1) 	41(1)	39(1) 	8(1)	16(1) 	-1(1)
C(16)	39(1) 	51(1)	34(1) 	1(1)	18(1) 	-1(1)
O(17)	78(1) 	70(1)	35(1) 	-3(1)	24(1) 	2(1)
C(18)	39(1) 	67(1)	49(1) 	6(1)	12(1) 	3(1)
C(19)	38(1) 	45(1)	36(1) 	0(1)	14(1) 	-4(1)
C(20)	41(1) 	47(1)	46(1) 	4(1)	19(1) 	-4(1)
C(21)	54(1) 	87(2)	78(1) 	27(1)	22(1) 	-20(1)
C(22)	60(1) 	66(1)	101(2) 	-24(1)	52(1) 	-7(1)
______________________________________________________________________________
Table 5. Hydrogen coordinates (x 104) and isotropic displacement parameters (Å2x 103) for KPP-II-50.
________________________________________________________________________________
	x 	y 	z 	U(eq)
________________________________________________________________________________

H(3)	5328	3335	5327	43
H(5)	3433	5827	7280	47
H(6A)	4906	7860	7097	54
H(6B)	5714	7293	8677	54
H(9)	8370	9300	9144	76
H(10)	10799	9722	9710	94
H(11)	12045	7833	9037	91
H(12)	10902	5458	7736	76
H(14A)	5536	1113	6680	51
H(14B)	6775	2131	7917	51
H(15)	5022	1429	8678	50
H(18A)	1275	4754	4109	82
H(18B)	1329	4890	5585	82
H(18C)	679	3267	4664	82
H(19)	2976	2615	4880	49
H(21A)	2522	564	7778	92
H(21B)	1285	1168	6260	92
H(22A)	8768	3577	5707	105
H(22B)	7589	2489	5872	105
H(22C)	9181	2600	7109	105
________________________________________________________________________________
Table 6. Torsion angles [°] for KPP-II-50.
________________________________________________________________
C(13)-N(1)-C(2)-C(7)	0.2(2)	C(22)-N(1)-C(2)-C(7)	176.1(2)
C(13)-N(1)-C(2)-C(3)	-174.71(14)	C(22)-N(1)-C(2)-C(3)	1.2(3)
C(7)-C(2)-C(3)-N(4)	20.7(2)	N(1)-C(2)-C(3)-N(4)	-165.14(14)
C(7)-C(2)-C(3)-C(14)	-101.03(18)	N(1)-C(2)-C(3)-C(14)	73.09(18)
C(2)-C(3)-N(4)-C(5)	-52.90(16)	C(14)-C(3)-N(4)-C(5)	68.39(16)
C(2)-C(3)-N(4)-C(19)	-172.52(11)	C(14)-C(3)-N(4)-C(19)	-51.24(15)
C(3)-N(4)-C(5)-C(6)	71.37(16)	C(19)-N(4)-C(5)-C(6)	-171.02(13)
C(3)-N(4)-C(5)-C(16)	-53.27(16)	C(19)-N(4)-C(5)-C(16)	64.34(16)
N(4)-C(5)-C(6)-C(7)	-48.69(18)	C(16)-C(5)-C(6)-C(7)	74.05(17)
N(1)-C(2)-C(7)-C(8)	0.18(19)	C(3)-C(2)-C(7)-C(8)	174.92(15)
N(1)-C(2)-C(7)-C(6)	-176.31(15)	C(3)-C(2)-C(7)-C(6)	-1.6(2)
C(5)-C(6)-C(7)-C(2)	14.6(2)	C(5)-C(6)-C(7)-C(8)	-160.79(17)
C(2)-C(7)-C(8)-C(9)	-178.8(2)	C(6)-C(7)-C(8)-C(9)	-2.9(3)
C(2)-C(7)-C(8)-C(13)	-0.49(18)	C(6)-C(7)-C(8)-C(13)	175.40(18)
C(13)-C(8)-C(9)-C(10)	-0.2(3)	C(7)-C(8)-C(9)-C(10)	178.0(2)
C(8)-C(9)-C(10)-C(11)	-0.6(3)	C(9)-C(10)-C(11)-C(12)	0.6(4)
C(10)-C(11)-C(12)-C(13)	0.3(3)	C(2)-N(1)-C(13)-C(12)	177.44(18)
C(22)-N(1)-C(13)-C(12)	1.5(3)	C(2)-N(1)-C(13)-C(8)	-0.5(2)
C(22)-N(1)-C(13)-C(8)	-176.5(2)	C(11)-C(12)-C(13)-N(1)	-178.88(19)
C(11)-C(12)-C(13)-C(8)	-1.2(3)	C(9)-C(8)-C(13)-N(1)	179.26(16)
C(7)-C(8)-C(13)-N(1)	0.63(19)	C(9)-C(8)-C(13)-C(12)	1.1(3)
C(7)-C(8)-C(13)-C(12)	-177.49(17)	N(4)-C(3)-C(14)-C(15)	-13.88(17)
C(2)-C(3)-C(14)-C(15)	105.77(15)	C(3)-C(14)-C(15)-C(16)	-48.74(17)
C(3)-C(14)-C(15)-C(20)	64.37(16)	C(20)-C(15)-C(16)-O(17)	127.24(18)
C(14)-C(15)-C(16)-O(17)	-117.71(18)	C(20)-C(15)-C(16)-C(5)	-50.81(16)
C(14)-C(15)-C(16)-C(5)	64.23(17)	N(4)-C(5)-C(16)-O(17)	170.62(16)
C(6)-C(5)-C(16)-O(17)	46.6(2)	N(4)-C(5)-C(16)-C(15)	-11.32(17)
C(6)-C(5)-C(16)-C(15)	-135.30(14)	C(3)-N(4)-C(19)-C(18)	-162.24(14)
C(5)-N(4)-C(19)-C(18)	79.53(17)	C(3)-N(4)-C(19)-C(20)	70.08(16)
C(5)-N(4)-C(19)-C(20)	-48.15(18)	C(16)-C(15)-C(20)-C(21)	-111.0(2)
C(14)-C(15)-C(20)-C(21)	133.9(2)	C(16)-C(15)-C(20)-C(19)	67.80(17)
C(14)-C(15)-C(20)-C(19)	-47.33(19)	N(4)-C(19)-C(20)-C(21)	160.8(2)
C(18)-C(19)-C(20)-C(21)	34.4(3)	N(4)-C(19)-C(20)-C(15)	-18.0(2)
C(18)-C(19)-C(20)-C(15)	-144.29(16)	________________________________________________________________
