# Supplementary figures and images for: First Total Synthesis of the Unnatural (+)-Talcarpine and (−)‑N 4‑Methyl,N 4‑21-secotalpinine
Source: ACS Omega. 2026 Apr 29;11(18):26942–56. doi: 10.1021/acsomega.5c13509 (PMC13176970; doi:10.1021/acsomega.5c13509)

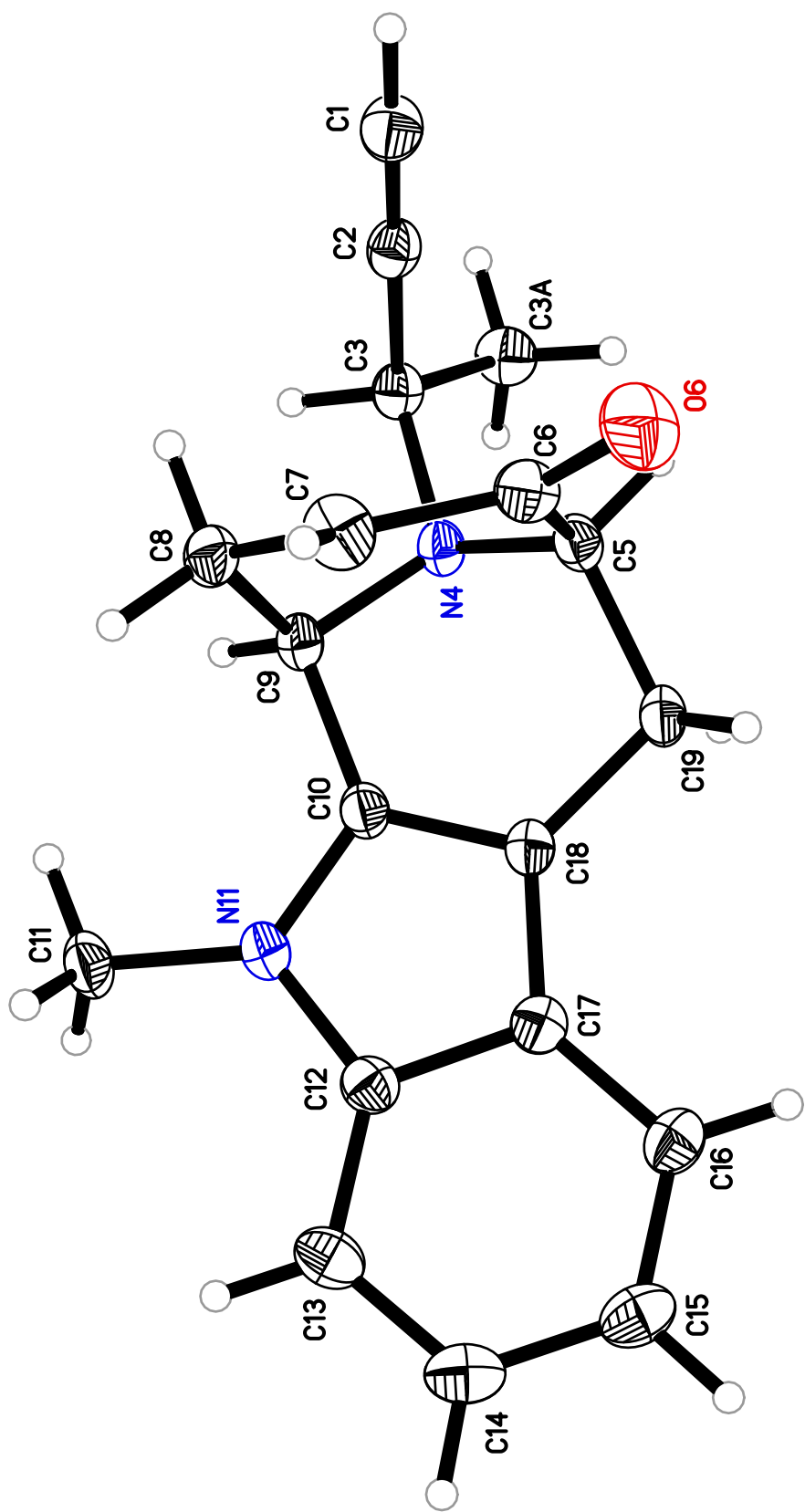

Supplement: Supplementary file 1 [file ao5c13509_si_001.zip › X-ray data for review/Cook 185 (compound 19)/cook185 (3).pdf]

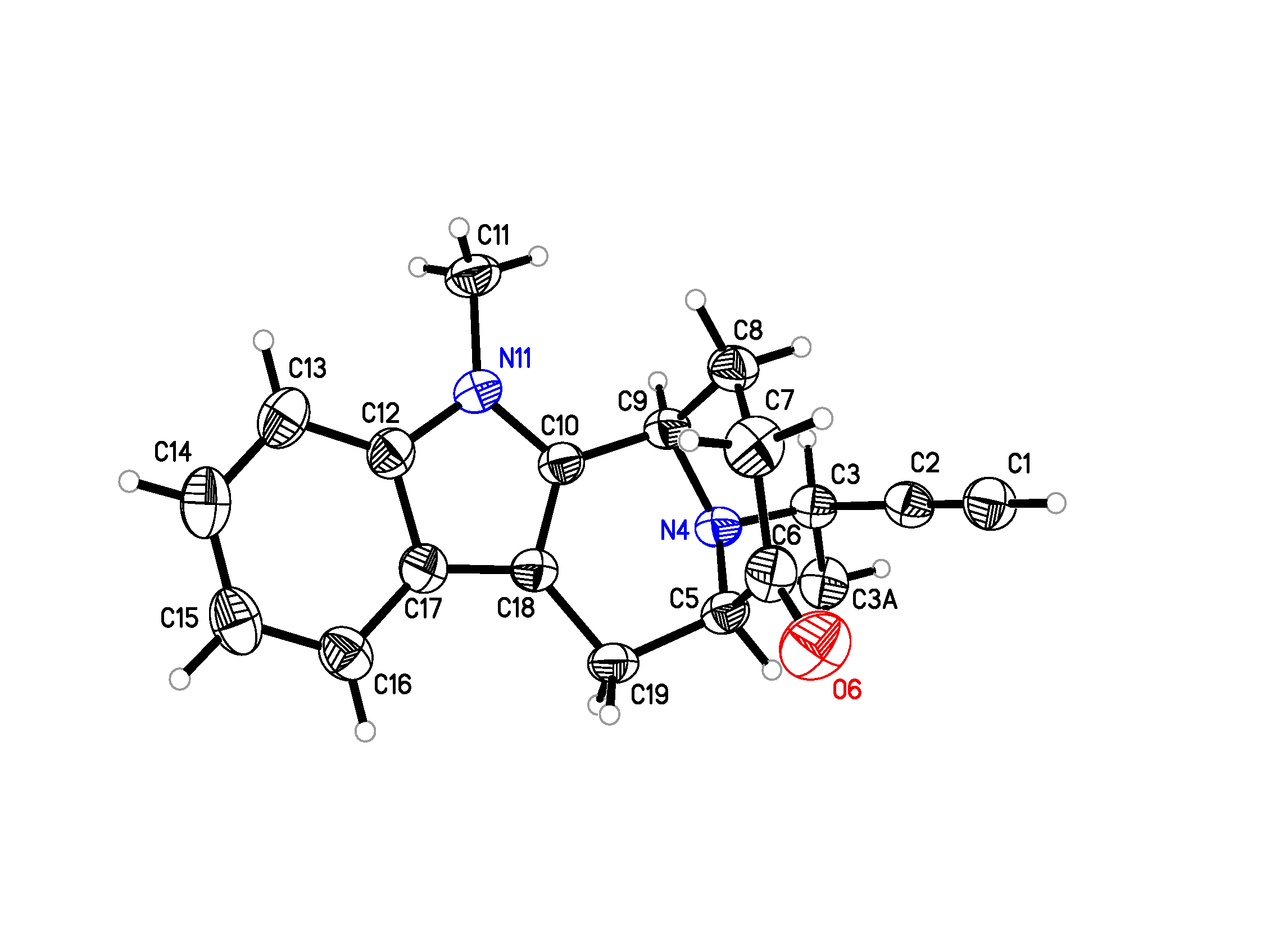

Supplement: Supplementary file 1 [file ao5c13509_si_001.zip › X-ray data for review/Cook 185 (compound 19)/cook185_plot1.tif]

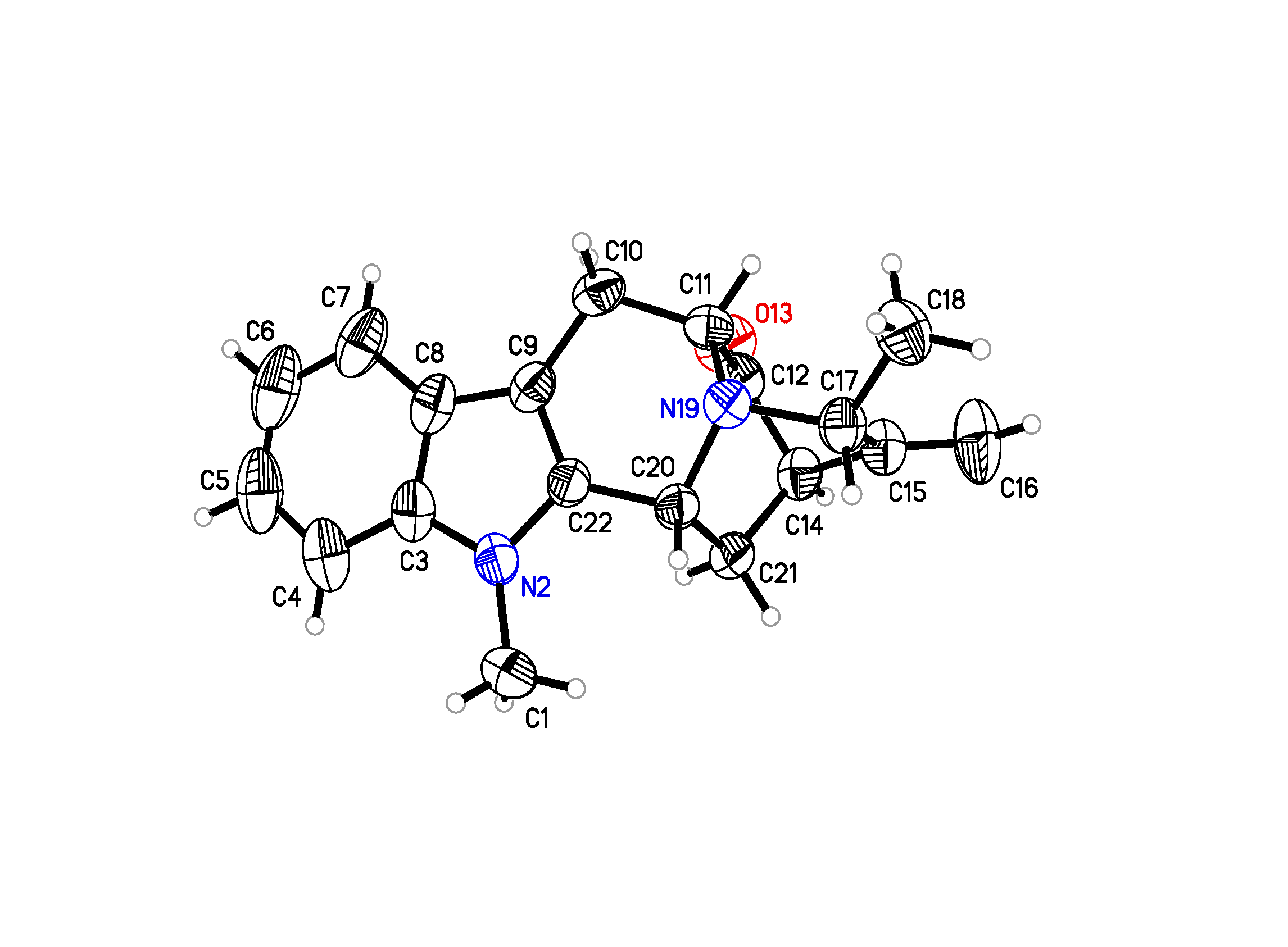

Supplement: Supplementary file 1 [file ao5c13509_si_001.zip › X-ray data for review/cook 196 (compound 11)/cook196_plot1.tif]

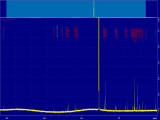

Supplement: Supplementary file 2 [file ao5c13509_si_002.zip › FID for publications/1/13C NMR/pdata/1/thumb.png]

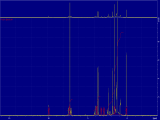

Supplement: Supplementary file 2 [file ao5c13509_si_002.zip › FID for publications/1/1H NMR/pdata/1/thumb.png]

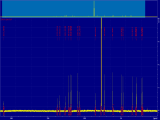

Supplement: Supplementary file 2 [file ao5c13509_si_002.zip › FID for publications/19/13C NMR/pdata/1/thumb.png]
