# Supplementary material for: First Total Synthesis of the Unnatural (+)-Talcarpine and (−)‑N 4‑Methyl,N 4‑21-secotalpinine
Source: ACS Omega. 2026 Apr 29;11(18):26942–56. doi: 10.1021/acsomega.5c13509 (PMC13176970; doi:10.1021/acsomega.5c13509)
Supplement: Supplementary file 2 [file ao5c13509_si_002.zip › FID for publications/1/HH COSY/pdata/1/email_KPP-IV-75 A_6_1.pdf]

HH COSY
cosy CDCl3 /nmr500 kppandey 24

UNIVERSITY of WISCONSIN
UWMILWAUKEE

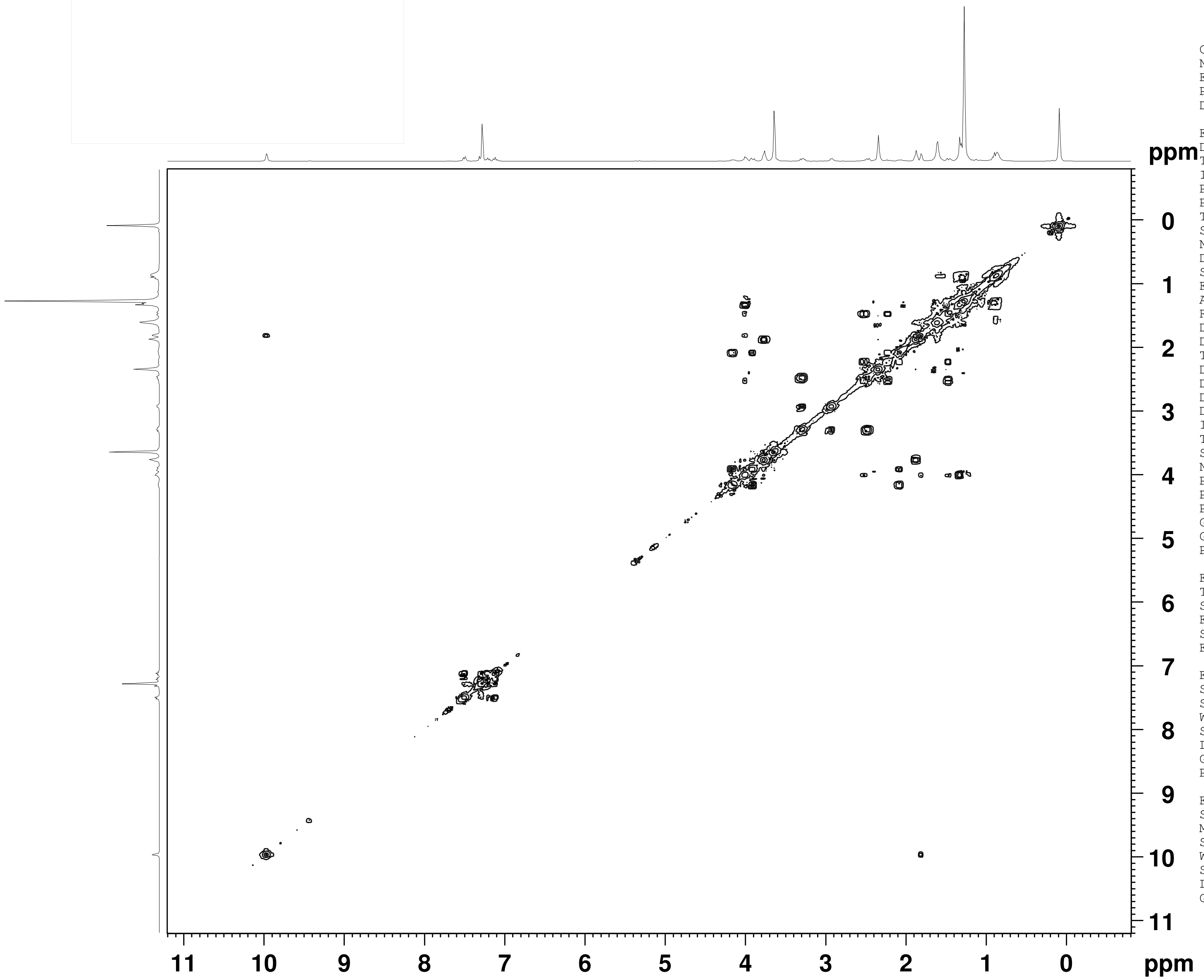


```
Current Data Parameters
NAME          KPP-IV-75 A
EXPNO                   6
PROCNO                  1
DATPATH   /nmr500/data/kppandey/nmr

F2 - Acquisition Parameters
Date_            20220820
Time                13.09 h
INSTRUM             spect
PROBHD     Z149001_0007 (
PULPROG          cosygpqf
TD                   2048
SOLVENT             CDCl3
NS                     30
DS                     16
SWH              6009.615 Hz
FIDRES           5.868765 Hz
AQ              0.1703936 sec
RG                 190.86
DW                 83.200 usec
DE                  25.00 usec
TE                  298.0 K
D0             0.00000300 sec
D1             1.50000000 sec
D13            0.00000400 sec
D16            0.00020000 sec
IN0            0.00016660 sec
TDav                    1
SFO1          500.1326007 MHz
NUC1                   1H
P0                  12.00 usec
P1                  12.00 usec
PLW1          14.14599991 W
GPNAM[1]       SMSQ10.100
GPZ1                10.00 %
P16               1000.00 usec

F1 - Acquisition parameters
TD                    128
SFO1             500.1326 MHz
FIDRES          93.787514 Hz
SW                 12.002 ppm
FnMODE                 QF

F2 - Processing parameters
SI                   1024
SF            500.1300000 MHz
WDW                  SINE
SSB                     0
LB                      0 Hz
GB                      0
PC                   1.40

F1 - Processing parameters
SI                   1024
MC2                    QF
SF            500.1300000 MHz
WDW                  SINE
SSB                     0
LB                      0 Hz
GB                      0
```
