# Supplementary material for: First Total Synthesis of the Unnatural (+)-Talcarpine and (−)‑N 4‑Methyl,N 4‑21-secotalpinine
Source: ACS Omega. 2026 Apr 29;11(18):26942–56. doi: 10.1021/acsomega.5c13509 (PMC13176970; doi:10.1021/acsomega.5c13509)
Supplement: Supplementary file 2 [file ao5c13509_si_002.zip › FID for publications/11/13C NMR/pdata/1/email_KPP-II-50-Dec 23-21_2_1.pdf]

Proton Spectrum -4-16ppm ns=16
c13 CDCl3 /nmr500 kppandey 10

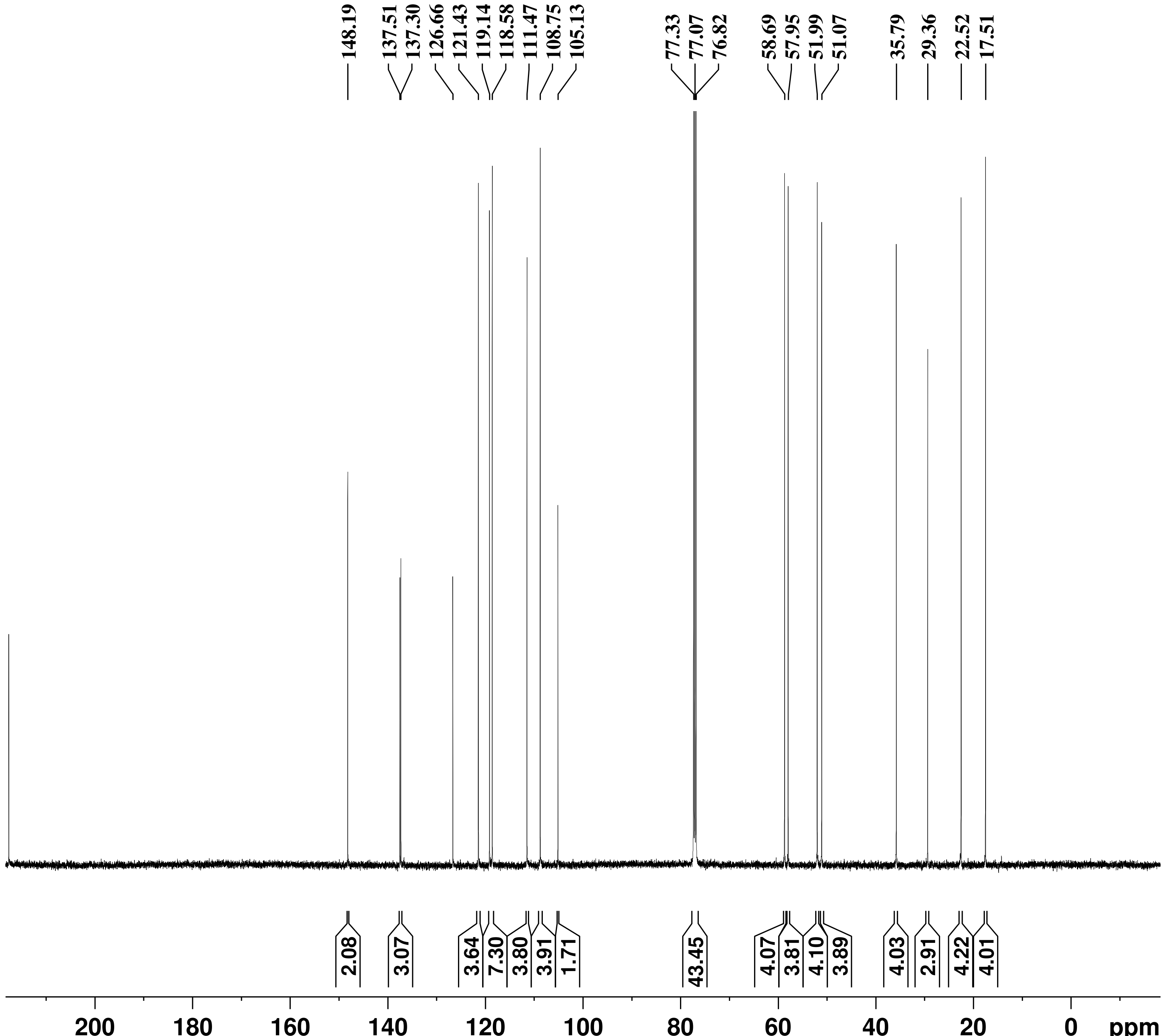

UNIVERSITY of WISCONSIN
UWMILWAUKEE

```
Current Data Parameters
NAME      KPP-II-50-Dec 23-21
EXPNO                      2
PROCNO                     1
DATPATH  /nmr500/data/kppandey/nmr

F2 - Acquisition Parameters
Date_             20211213
Time                 18.49 h
INSTRUM              spect
PROBHD    Z149001_0007 (
PULPROG            zgpg30
TD                  65536
SOLVENT             CDCl3
NS                    300
DS                      0
SWH            29761.904 Hz
FIDRES          0.908261 Hz
AQ             1.1010048 sec
RG                190.86
DW                16.800 usec
DE                 22.42 usec
TE                 298.0 K
D1            2.00000000 sec
D11           0.03000000 sec
TD0                     1
SFO1         125.7703643 MHz
NUC1                  13C
P0                   3.33 usec
P1                  10.00 usec
PLW1          64.21800232 W
SFO2         500.1320005 MHz
NUC2                   1H
CPDPRG[2          waltz16
PCPD2               80.00 usec
PLW2          14.14599991 W
PLW12          0.31829000 W
PLW13          0.16010000 W

F2 - Processing parameters
SI                  65536
SF           125.7577885 MHz
WDW                    EM
SSB                     0
LB                   1.00 Hz
GB                      0
PC                   1.40
```
