# Supplementary material for: First Total Synthesis of the Unnatural (+)-Talcarpine and (−)‑N 4‑Methyl,N 4‑21-secotalpinine
Source: ACS Omega. 2026 Apr 29;11(18):26942–56. doi: 10.1021/acsomega.5c13509 (PMC13176970; doi:10.1021/acsomega.5c13509)
Supplement: Supplementary file 2 [file ao5c13509_si_002.zip › FID for publications/11/1H NMR/pdata/1/email_KPP-II-50-Dec 23-21_1_1.pdf]

Proton Spectrum -4-16ppm ns=16
h1 CDCl3 /nmr500 kppandey 10

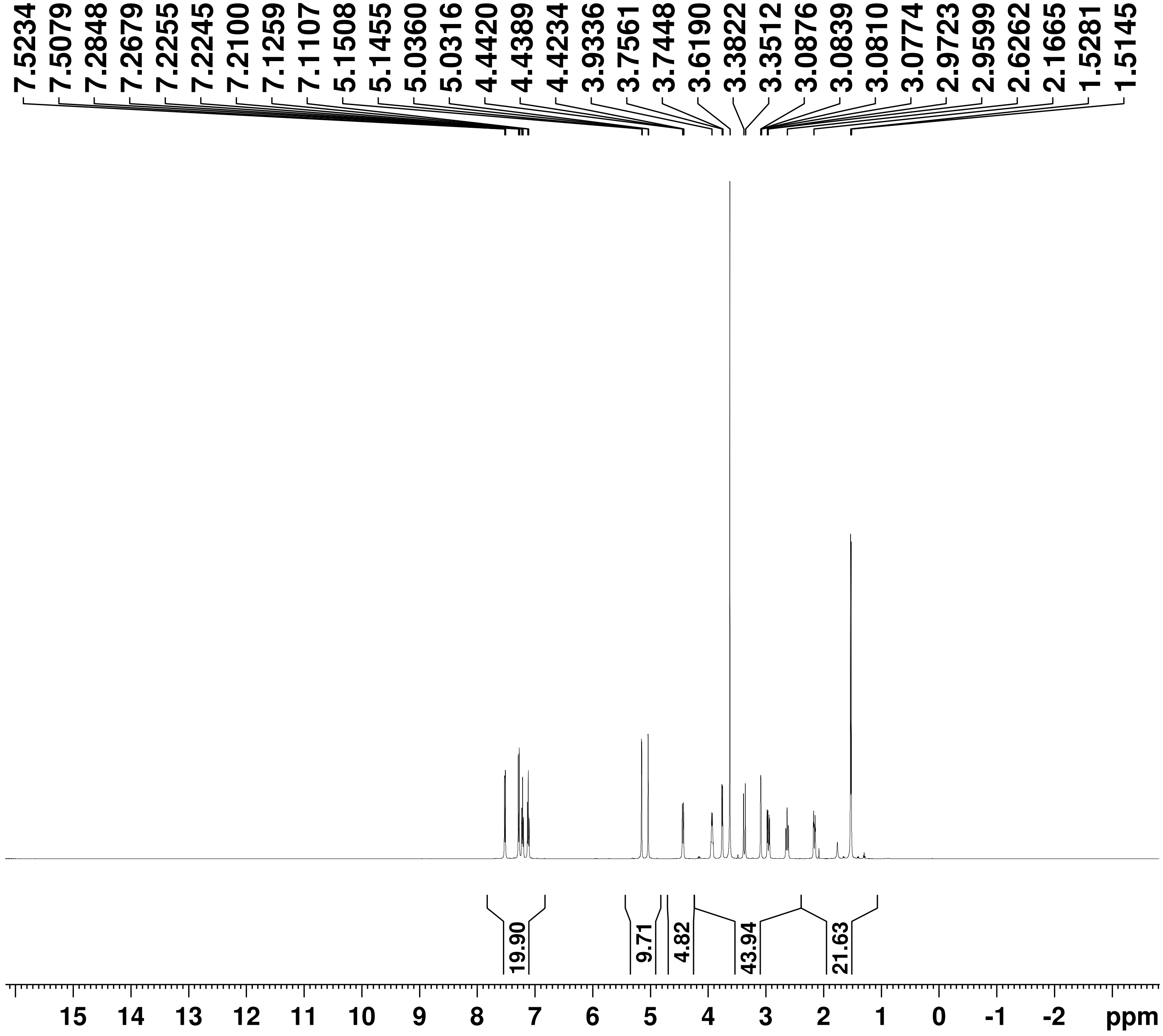

UNIVERSITY of WISCONSIN UW MILWAUKEE

```
Current Data Parameters
NAME      KPP-II-50-Dec 23-21
EXPNO                   1
PROCNO                  1
DATPATH   /nmr500/data/kppandey/nmr

F2 - Acquisition Parameters
Date_           20211213
Time               18.32 h
INSTRUM            spect
PROBHD   Z149001_0007 (
PULPROG             zg30
TD                 65536
SOLVENT            CDCl3
NS                    25
DS                     0
SWH            10000.000 Hz
FIDRES          0.305176 Hz
AQ             3.2767999 sec
RG                 47.99
DW                50.000 usec
DE                 25.00 usec
TE                 298.0 K
D1            1.00000000 sec
TD0                    1
SFO1         500.1330883 MHz
NUC1                  1H
P0                  4.00 usec
P1                 12.00 usec
PLW1         14.14599991 W

F2 - Processing parameters
SI                 65536
SF           500.1300000 MHz
WDW                   EM
SSB                    0
LB                  0.30 Hz
GB                     0
PC                  1.00
```
