# Supplementary material for: First Total Synthesis of the Unnatural (+)-Talcarpine and (−)‑N 4‑Methyl,N 4‑21-secotalpinine
Source: ACS Omega. 2026 Apr 29;11(18):26942–56. doi: 10.1021/acsomega.5c13509 (PMC13176970; doi:10.1021/acsomega.5c13509)
Supplement: Supplementary file 3 [file ao5c13509_si_003.pdf]

First Total Synthesis of the Unnatural (+)-Talcarpine, and (-)-N_4-methyl, N_4-21-*seco*talpinine.

Kamal Prasad Pandey*[1]; Md Toufiqur Rahman[2]; Anna Benko[1]; Gregory H. Imler[3]; Jefferey R. Deschamps[3]; James M. Cook[1]

1 Department of Chemistry and Biochemistry, University of Wisconsin-Milwaukee, Milwaukee, WI 53211, USA.
2 RTI International, Center for Therapeutics, Toxicology, and Devices, Research Triangle Park, Durham, NC 27713
3 Naval Research Laboratory, 4555 Overlook Avenue, Washington, DC 20375
* Correspondence: kppandey@uwm.edu

Table of contents

Supplementary Tables *8*

Table S1. Optical rotation data of the synthetic unnatural intermediates and synthetic natural intermediates. 8

Table S2. Comparison of ^{1}H NMR ($CDCl_3$) spectroscopic data of the natural (-)-talcarpine and unnatural (synthetic) (+)-talcarpine (1). 9

NMR analysis of intermediates and final products. *10*

1.1. ^{1}H NMR of (6*R*,10R)-5-methyl-5,6,7,8,10,11-hexahydro-9H-6,10-epiminocycloocta[b]indol-9-one (4). 11

1.2. ^{13}C NMR of (6*R*,10*R*)-5-methyl-5,6,7,8,10,11-hexahydro-9*H*-6,10-epiminocycloocta[b]indol-9-one (4). 12

1.3. ^{1}H NMR of (6*R*,10*R*)-5-methyl-12-((*R*)-4-(triisopropylsilyl)but-3-yn-2-yl)-5,6,7,8,10,11-hexahydro-9*H*-6,10-epiminocycloocta[b]indol-9-one (22). 13

1.4. ^{13}C NMR of (6*R*,10*R*)-5-methyl-12-((*R*)-4-(triisopropylsilyl)but-3-yn-2-yl)-5,6,7,8,10,11-hexahydro-9*H*-6,10-epiminocycloocta[b]indol-9-one (22). 14

1.5. ^{1}H NMR of (6*R*,10*R*)-12-((*R*)-but-3-yn-2-yl)-5-methyl-5,6,7,8,10,11-hexahydro-9*H*-6,10-epiminocycloocta[b]indol-9-one (19). 15

1.6. ^{13}C NMR of (6*R*,10*R*)-12-((*R*)-but-3-yn-2-yl)-5-methyl-5,6,7,8,10,11-hexahydro-9*H*-6,10-epiminocycloocta[b]indol-9-one (19). 16

1.7. ^{1}H NMR of (6R,10R)-12-((R)-3-iodobut-3-en-2-yl)-5-methyl-5,6,7,8,10,11-hexahydro-9H-6,10-epiminocycloocta[b]indol-9-one (23) 17

1.8. ^{13}C NMR of 6*R*,10*R*)-12-((*R*)-3-iodobut-3-en-2-yl)-5-methyl-5,6,7,8,10,11-hexahydro-9*H*-6,10-epiminocycloocta[b]indol-9-one (23) 18

1.9. ^{1}H NMR of (6*R*,7*R*,8*R*,10*R*,11a*R*)-5,8-dimethyl-9-methylene-5,6,9,10,11a,12-hexahydro-6,10-methanoindolo[3,2-b]quinolizin-11(8H)-one (11). 19

1.10. ^{13}C NMR of provide (6*R*,7*R*,8*R*,10*R*,11a*R*)-5,8-dimethyl-9-methylene-5,6,9,10,11a,12-hexahydro-6,10-methanoindolo[3,2-b]quinolizin-11(8H)-one (11). 20

1.11. ^{1}H NMR of ((6*R*,7*R*,8*R*,10*S*,11*S*,11a*R*)-5,8-dimethyl-9-methylene-5,6,8,9,10,11,11a,12-octahydro-6,10-methanoindolo[3,2-b]quinolizin-11-yl)methanol (26). 21

1.12. ^{13}C NMR of ((6*R*,7*R*,8*R*,10*S*,11*S*,11a*R*)-5,8-dimethyl-9-methylene-5,6,8,9,10,11,11a,12-octahydro-6,10-methanoindolo[3,2-b]quinolizin-11-yl)methanol (26). 22

1.13. DEPT-135 ^{13}C NMR of ((6*R*,7*R*,8*R*,10*S*,11*S*,11a*R*)-5,8-dimethyl-9-methylene-5,6,8,9,10,11,11a,12-octahydro-6,10-methanoindolo[3,2-b]quinolizin-11-yl)methanol (26).. 23

1.14. ^{1}H NMR of (6*R*,7*R*,8*R*,10*S*,11*S*,11a*R*)-5,8-dimethyl-9-methylene-11-(((triisopropylsilyl)oxy) methyl)-5,6,8,9,10,11,11a,12-octahydro-6,10-methanoindolo[3,2-b]quinolizine (27)........ 24

1.15. ^{13}C NMR of (6*R*,7*R*,8*R*,10*S*,11*S*,11a*R*)-5,8-dimethyl-9-methylene-11-(((triisopropylsilyl)oxy) methyl)-5,6,8,9,10,11,11a,12-octahydro-6,10-methanoindolo[3,2-b]quinolizine (27)........ 25

1.16. DEPT-135 ^{13}C NMR of (6*R*,7*R*,8*R*,10*S*,11*S*,11a*R*)-5,8-dimethyl-9-methylene-11-(((triisopropylsilyl)oxy) methyl)-5,6,8,9,10,11,11a,12-octahydro-6,10-methanoindolo[3,2-b]quinolizine (27)........ 26

1.17. ^{1}H NMR of ((6*R*,7*S*,8*R*,9*R*,10*R*,11*S*,11a*R*)-5,8-dimethyl-11-(((triisopropylsilyl)oxy)methyl)-5,6,8,9,10,11,11a,12-octahydro-6,10-methanoindolo[3,2-b]quinolizin-9-yl)methanol (28). 27

1.18. ^{13}C NMR of ((6R,7S,8R,9R,10R,11S,11aR)-5,8-dimethyl-11-(((triisopropylsilyl)oxy)methyl)-5,6,8,9,10,11,11a,12-octahydro-6,10-methanoindolo[3,2-b]quinolizin-9-yl)methanol (28). 28

1.19. DEPT-135 ^{13}C NMR of ((6R,7S,8R,9R,10R,11S,11aR)-5,8-dimethyl-11-(((triisopropylsilyl)oxy)methyl)-5,6,8,9,10,11,11a,12-octahydro-6,10-methanoindolo[3,2-b]quinolizin-9-yl)methanol (28). 29

1.20. ^{1}H NMR of 6R,7S,8R,9R,10S,11S,11aR)-5,8-dimethyl-11-(((triisopropylsilyl)oxy) methyl)-5,6,8,9,10,11,11a,12-octahydro-6,10-methanoindolo[3,2-b]quinolizine-9-carbaldehyde (29)........ 30

1.21. ^{13}C NMR of 6R,7S,8R,9R,10S,11S,11aR)-5,8-dimethyl-11-(((triisopropylsilyl)oxy) methyl)-5,6,8,9,10,11,11a,12-octahydro-6,10-methanoindolo[3,2-b]quinolizine-9-carbaldehyde (29)........ 31

1.22. DEPT-135 ^{13}C NMR of 6R,7S,8R,9R,10S,11S,11aR)-5,8-dimethyl-11-(((triisopropylsilyl)oxy) methyl)-5,6,8,9,10,11,11a,12-octahydro-6,10-methanoindolo[3,2-b]quinolizine-9-carbaldehyde (29)........ 32

1.24. ^{1}H NMR spectra of (+)-talcarpine (1). 33

1.25. ^{13}C NMR spectra of (+)-talcarpine (1). 34

1.26. ^{1}H-^{1}H COSY spectra of (+)-talcarpine (1) 35

HRMS and LCMS spectra (data) 36

2.1. HRMS analysis of (6R,10R)-12-((R)-but-3-yn-2-yl)-5-methyl-5,6,7,8,10,11-hexahydro-9H-6,10-epiminocycloocta[b]indol-9-one (4)........ 36

2.2. HRMS analysis of (6*R*,10*R*)-12-benzyl-5-methyl-5,6,7,8,10,11-hexahydro-9*H*-6,10-epiminocycloocta[b]indol-9-one (15)........ 37

2.3. HRMS analysis of ((6*R*,7*R*,8*R*,10*S*,11*S*,11a*R*)-5,8-dimethyl-9-methylene-5,6,8,9,10,11,11a,12-octahydro-6,10-methanoindolo[3,2-b]quinolizin-11-yl)methanol (26).. 38

2.4. HRMS analysis of (6*R*,7*R*,8*R*,10*S*,11*S*,11a*R*)-5,8-dimethyl-9-methylene-11-(((triisopropylsilyl)oxy) methyl)-5,6,8,9,10,11,11a,12-octahydro-6,10-methanoindolo[3,2-b]quinolizine (27)........ 39

2.5. HRMS analysis of ((6*R*,7*S*,8*R*,9*R*,10*R*,11*S*,11a*R*)-5,8-dimethyl-11-(((triisopropylsilyl)oxy)methyl)-5,6,8,9,10,11,11a,12-octahydro-6,10-methanoindolo[3,2-b]quinolizin-9-yl)methanol (28)........ 40

2.6. 6*R*,7*S*,8*R*,9*R*,10*S*,11*S*,11a*R*)-5,8-dimethyl-11-(((triisopropylsilyl)oxy) methyl)-5,6,8,9,10,11,11a,12-octahydro-6,10-methanoindolo[3,2-b]quinolizine-9-carbaldehyde (29). 41

2.7. LCMS analysis of ((6*R*,7*R*,8*R*,10*S*,11*S*,11a*R*)-5,8-dimethyl-9-methylene-5,6,8,9,10,11,11a,12-octahydro-6,10-methanoindolo[3,2-b]quinolizin-11-yl)methanol (30).. 42

2.8. HRMS analysis of (6*R*,7*S*,8*R*,9*R*,10*S*,11*S*,11a*R*)-9-formyl-5,7,8-trimethyl-11-(((triisopropylsilyl)oxy) methyl)-6,7,8,9,10,11,11a,12-octahydro-5*H*-6,10-methanoindolo[3,2-b]quinolizin-7-ium (30)........ 43

2.9. HRMS analysis of 31........ 44

2.10. LCMS anaysis of (+)-talcarpine (1)........ 45

2.11. HRMS analysis of (+)-talcarpine (1)........ 46

2.12. LCMS anaysis of (-) N_4 -methyl, N_4 -21 -secotalpinine (2)........ 47

2.13. HRMS analysis of (-) N_4 -methyl, N_4 -21 -secotalpinine 2........ 48

X ray crystallographic data of key intermediates......... *49*

3.1. Single-crystal X-ray Diffraction Analysis of pentacyclic ketone 11........ 49

3.2. X-ray Crystal Data for Compound 19........ 56

References........ *63*

Supplementary Tables

Table S1. Optical rotation data of the synthetic unnatural intermediates and synthetic natural intermediates.

Compound number	Optical rotation values of synthetic unnatural enantiomers	Literature values for the optical rotation values of synthetic natural enantiomers
**1**	$[\alpha]_D^{25}$ + 36.5(c 0.08, $CHCl_3$)	$[\alpha]_D^{25}$ – 30 (c 0.1, $CHCl_3$) [1]
**11**	$[\alpha]_D^{25}$ + 146.7 (c 0.98, $CHCl_3$),	$[\alpha]_D^{25}$ – 150.0 (c 1.0, $CHCl_3$) [1, 2]
**12**	$[\alpha]_D^{25}$ + 35.4 (c 1.18, MeOH)	$[\alpha]_D^{27}$ - 37.2 (c 1.0, MeOH) [3]
**13**	$[\alpha]_D^{25}$ - 9.9 (c 1.01, MeOH)	$[\alpha]_D^{27}$ + 9.07 (c 1.0, MeOH) [3,4]
**15**	$[\alpha]_D^{25}$ + 36.2 (c 1.02, $CHCl_3$)	$[\alpha]_D^{27}$ - 35.7 (c 1.4, $CHCl_3$) [3]
**16**	$[\alpha]_D^{25}$ + 51.9 (c 1.02 $CHCl_3$)	$[\alpha]_D^{25}$ - 54.6 (c 1.0, $CHCl_3$) [5]
**17**	$[\alpha]_D^{25}$ + 236.6 (c 1.0, $CHCl_3$)	$[\alpha]_D^{27}$ – 240.2 (c 1.0, $CHCl_3$) [4]
**19**	$[\alpha]_D^{25}$ +191.3 (c 1.0, $CHCl_3$)	$[\alpha]_D^{27}$ – 191.0 (c 0.192, $CHCl_3$) [4]

Table S2. Comparison of ^{1}H NMR ($CDCl_3$) spectroscopic data of the natural (-)-talcarpine and unnatural (synthetic) (+)-talcarpine (1).

Unnatural (synthetic) (+)-talcarpine **1** (500 MHz, $CDCl_3$)	Isolated natural enantiomer, (-)-talcarpine by Wong *et* al. 1996* (270 MHz, $CDCl_3$) [6]	Isolated natural enantiomer (-)-talcarpine by Kam *et* al. 2004 (400 Hz, $CDCl_3$) [7]
9.95 (s, 1H)	9.94 (d, J = 3 Hz, 1H)	9.95 (1H, d, J¼3 Hz)
7.48 (d, *J* = 7.8 Hz, 1H)	7.48 (br d, J = 7 Hz, 1H)	7.49 (1H, br d, J¼8 Hz)
7.28 (d, *J* = 8.4 Hz, 1H)	7.28 (br d, J = 7 Hz, 1H)	7.29 (1H, br d, J¼8 Hz)
7.19 (t, *J* = 7.4 Hz, 1H)	7.19 (br t, J = 7 Hz, 1H)	7.19 (1H, td, J¼8, 1 Hz)
7.10 (t, *J* = 7.0 Hz, 1H)	7.09 (br t, J = 7 Hz, 1H)	7.10 (1H, td, J¼8, 1 Hz)
4.14 (t, *J* = 11.5 Hz, 1H)	4.13 (t, J = 11 Hz, 1H)	4.14 (1H, t, J¼12 Hz)
3.98 (s, 2H)	3.92-4.05 (m, J = 7 Hz, 2H)	3.98 (2H, m)
3.89 (dd, *J* = 11.3, 4.8 Hz, 1H)	3.89 (dd, J = 11 Hz, 5Hz, 1H)	3.89 (1H, dd, J¼12, 5 Hz)
3.62 (s, 3H)	3.62 (s, 3H)	3.62 (3H, s)
3.27 (dd, *J* = 16.7, 6.7 Hz, 1H)	3.24 (dd, J = 16 Hz, 7 Hz, 1H)	3.27 (1H, dd, J¼16, 7 Hz)
2.91 (s, 1H)	2.89 (d, J = 7 Hz, 1H)	2.90 (1H, d, J¼7 Hz)
2.47 (m, *J* = 14.2 Hz, 2H) (peaks overlapped)	2.41-2.55 (m, 1H)	2.45 (1H, d, J¼16 Hz), and 2.50 (1H, td, J¼12, 4 Hz)
2.32 (s, 3H)	2.32 (s, 3H)	2.32 (3H, s)
2.20 (m, *J* = 13.0 Hz, 1H)	2.15-2.24 (m, 1H)	2.20 (1H, m, H-15)
2.05 (s, 1H)	2.00-2.09 (m, 1H)	2.06 (1H, dt, J¼11, 5 Hz)
1.79 (s, 1H)	1.78 (br s, 1H)	1.79 (1H, br s)
1.45 (d, *J* = 13.2 Hz, 1H)	1.40-1.48 (m, 1H)	1.45 (1H, ddd, J¼12, 4, 3 Hz)
1.30 (d, *J* = 6.6 Hz, 3H)	1.30 (d, J =6.8 Hz, 1H)	d 1.30 (3H, d, J¼7 Hz)

*^{1}H NMR of unnatural (+)-talcarpine was in excellent agreement with the natural enantiomer isolated by Kam et al. 2004 [7]. ^{1}H NMR of natural (+)-talcarpine reported by Wong et al. found to have missing number of protons.

Table S3. Comparison of ^{13}C NMR ($CDCl_3$) spectroscopic data of the natural (-)-talcarpine and unnatural (synthetic) (+)-talcarpine (1).

Unnatural (synthetic) enantiomer, (+)-talcarpine **1**, (126 MHz, $CDCl_3$)	Isolated natural enantiomer, (-)-talcarpine (67.8 MHz, $CDCl_3$) by Wong *et* al. 1996 [6]	Isolated natural enantiomer, (-)-talcarpine (100 MHz, $CDCl_3$) by Kam *et* al. 2004 [6]
δ 204.76	204.7	204.7
137.01	136.9	137.2
132.57	132.6	132.8
126.37	126.3	126.2
121.04	120.9	121.0
118.95	118.9	118.9
118.18	118.1	118.1
108.79	108.7	108.9
106.64	106.6	106.6
69.48	69.4	69.4
68.86	68.8	69.4
54.54	54.5	54.6
54.43	54.4	54.5
53.56	53.5	53.5
41.72	41.7	41.8
39.41	39.4	39.4
28.9	30.0	30.1
28.98	28.9	29.1
26.95	27.0	27.0
22.51	22.4	22.5
19.23	19.2	19.2

^{13}C NMR of unnatural (+)-talcarpine was in excellent agreement with the natural enantiomer isolated by Wong *et* al. 1996 [6] and Kam *et* al. 2004 [7].

NMR analysis of intermediates and final products.

1.1. ^{1}H NMR of (6*R*,10R)-5-methyl-5,6,7,8,10,11-hexahydro-9H-6,10-epiminocycloocta[b]indol-9-one (4).

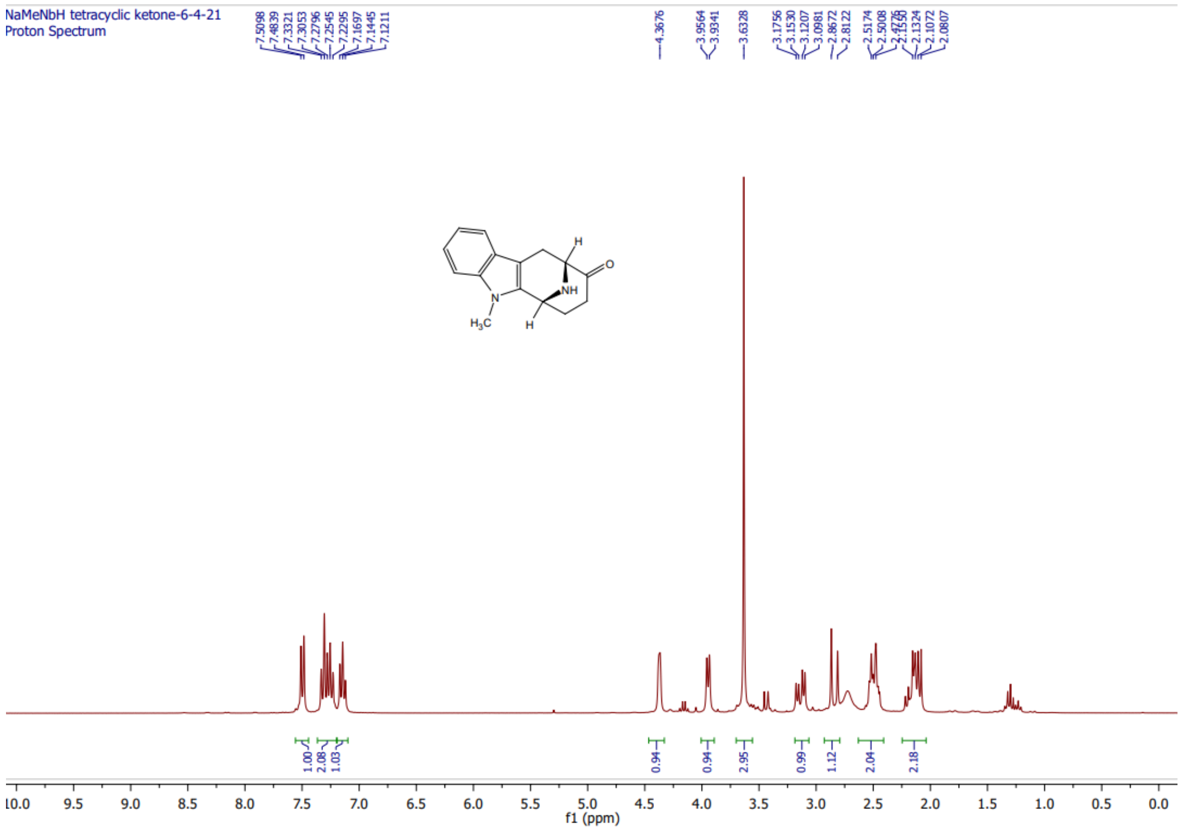

1.2. ^{13}C NMR of (6*R*,10*R*)-5-methyl-5,6,7,8,10,11-hexahydro-9*H*-6,10-epiminocycloocta[b]indol-9-one (4).

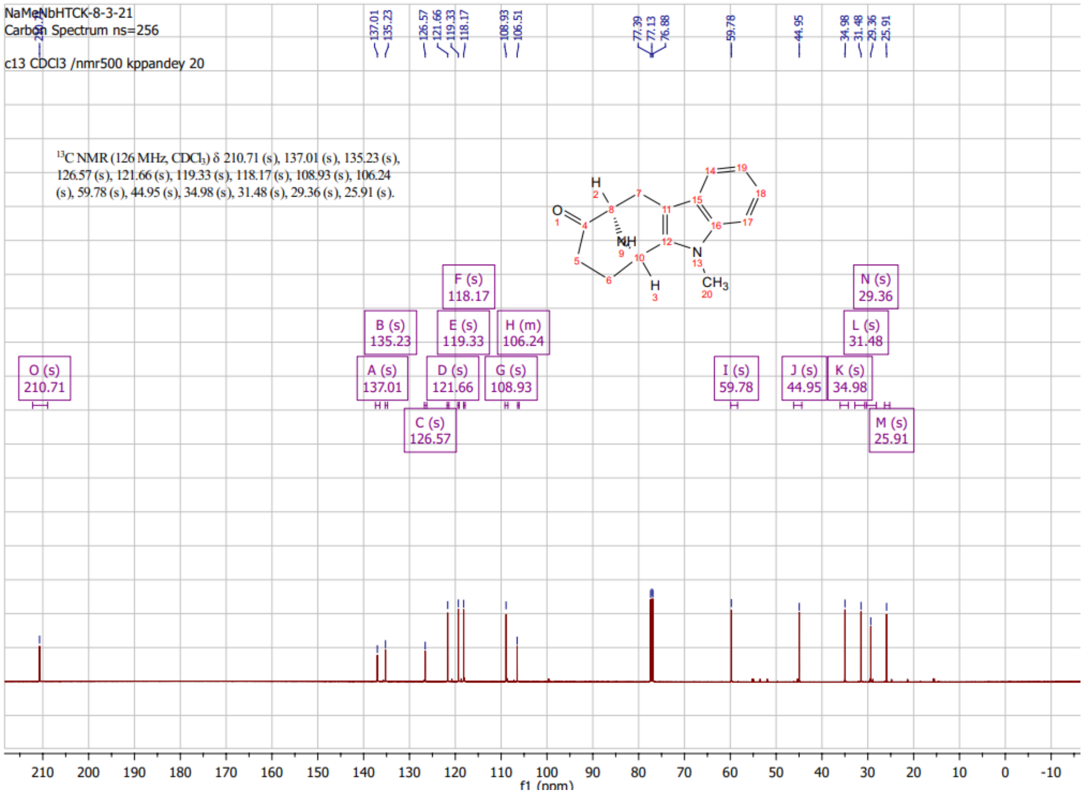

1.3. ^{1}H NMR of (6*R*,10*R*)-5-methyl-12-((*R*)-4-(triisopropylsilyl)but-3-yn-2-yl)-5,6,7,8,10,11-hexahydro-9*H*-6,10-epiminocycloocta[b]indol-9-one (22).

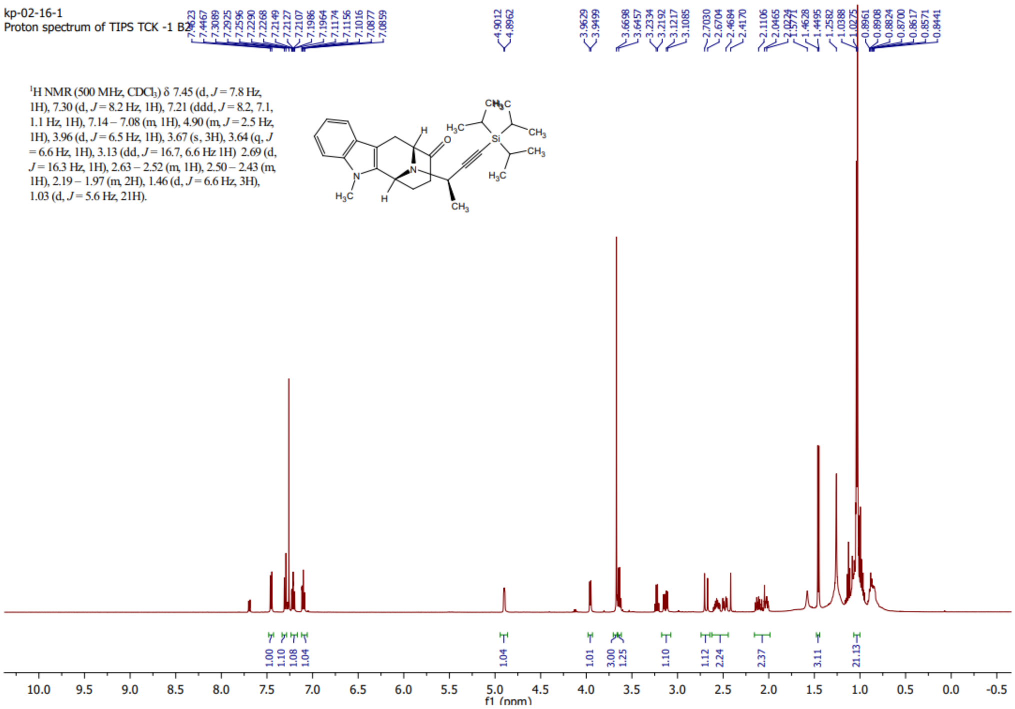

1.4. ^{13}C NMR of (6*R*,10*R*)-5-methyl-12-((*R*)-4-(triisopropylsilyl)but-3-yn-2-yl)-5,6,7,8,10,11-hexahydro-9*H*-6,10-epiminocycloocta[b]indol-9-one (22).

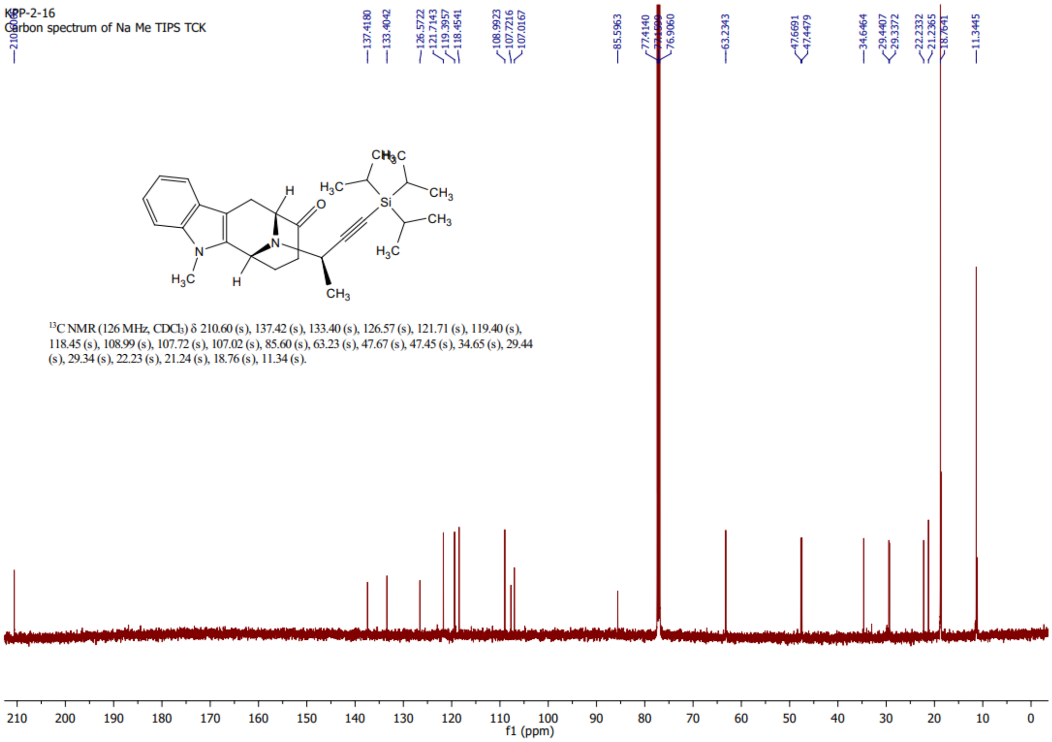

1.5. ^{1}H NMR of (6*R*,10*R*)-12-((*R*)-but-3-yn-2-yl)-5-methyl-5,6,7,8,10,11-hexahydro-9*H*-6,10-epiminocycloocta[b]indol-9-one (19).

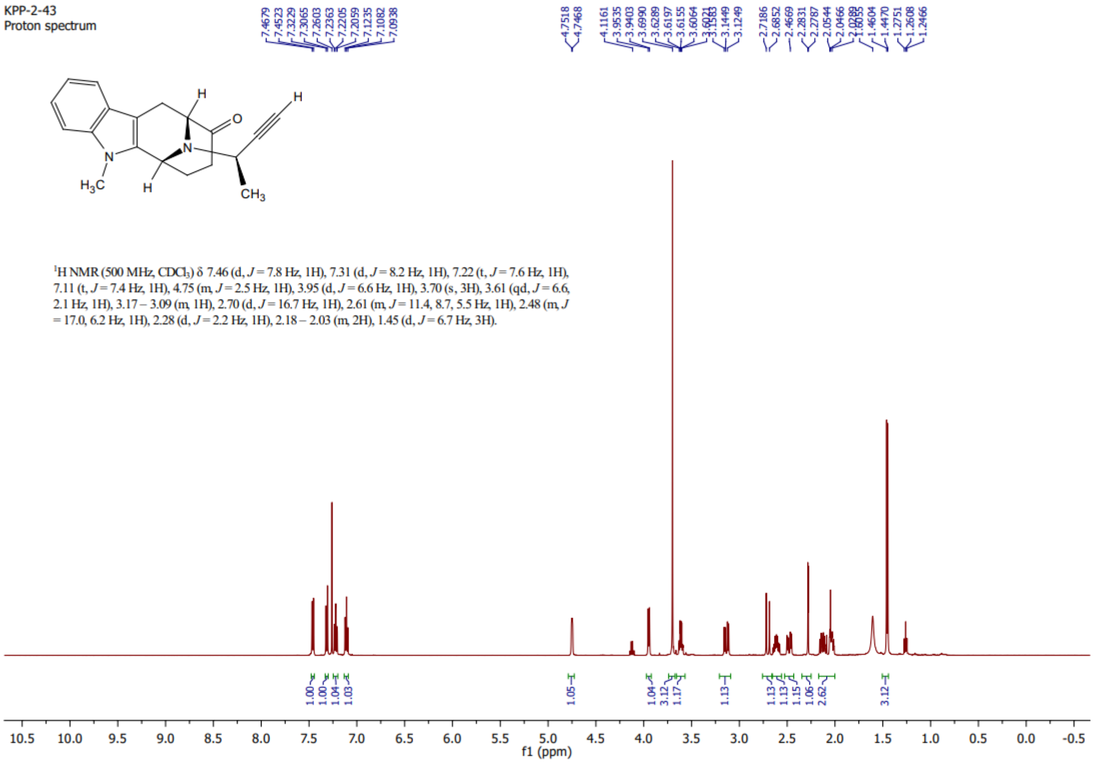

1.6. ^{13}C NMR of (6*R*,10*R*)-12-((*R*)-but-3-yn-2-yl)-5-methyl-5,6,7,8,10,11-hexahydro-9*H*-6,10-epiminocycloocta[b]indol-9-one (19).

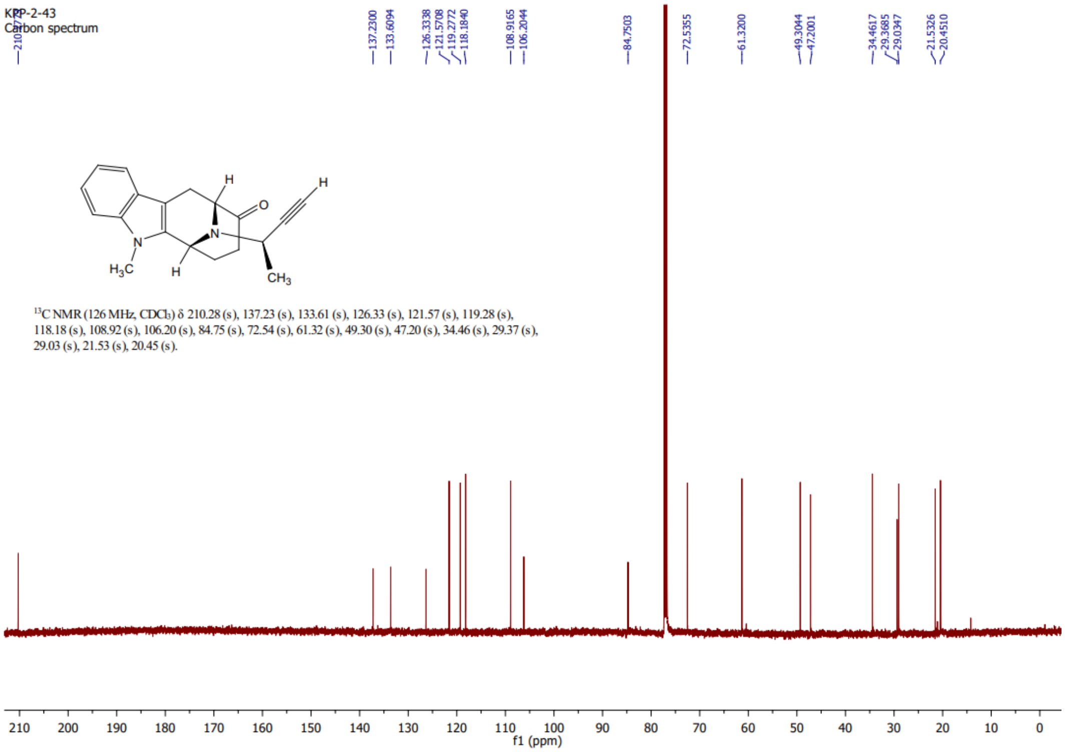

1.7. [13]H NMR of (6R,10R)-12-((R)-3-iodobut-3-en-2-yl)-5-methyl-5,6,7,8,10,11-hexahydro-9H-6,10-epiminocycloocta[b]indol-9-one (23).

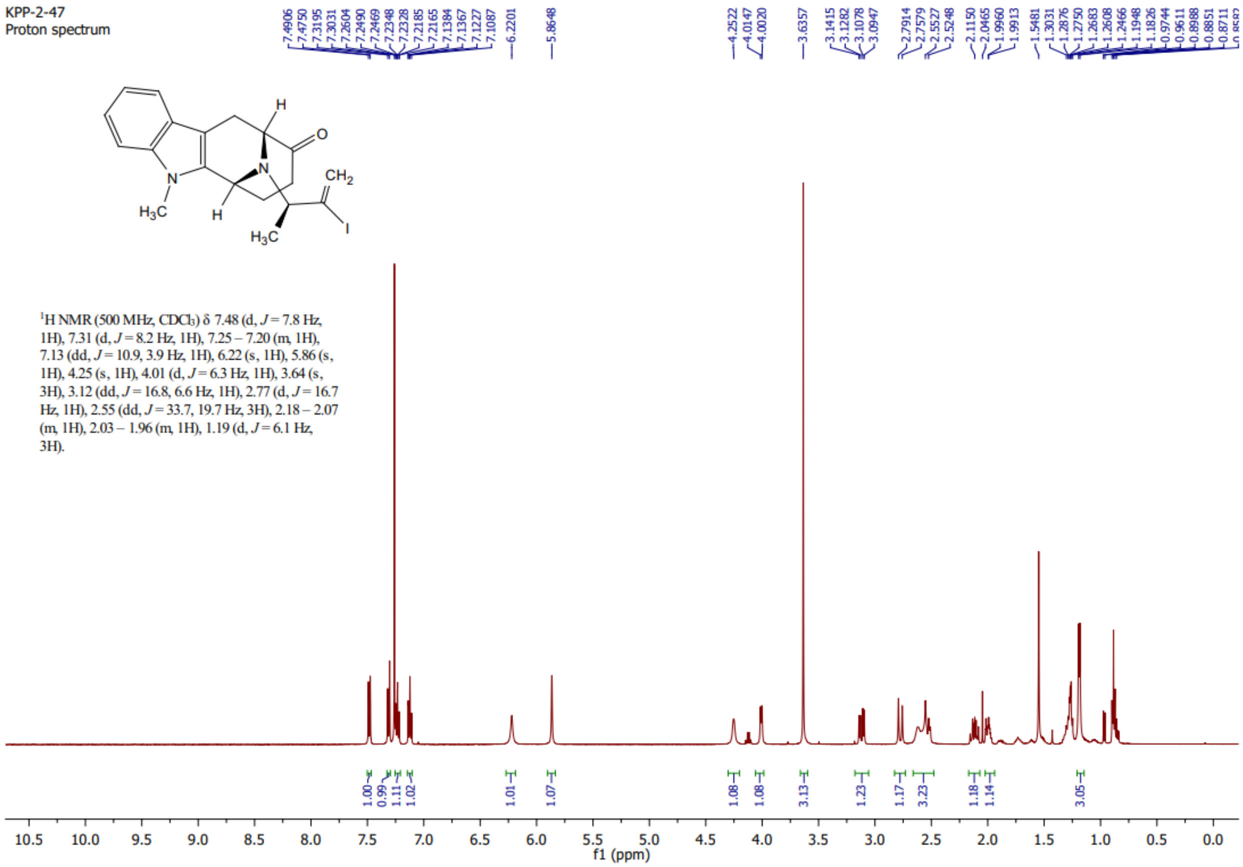

1.8. ^{13}C NMR of 6*R*,10*R*)-12-((*R*)-3-iodobut-3-en-2-yl)-5-methyl-5,6,7,8,10,11-hexahydro-9*H*-6,10-epiminocycloocta[b]indol-9-one (23).

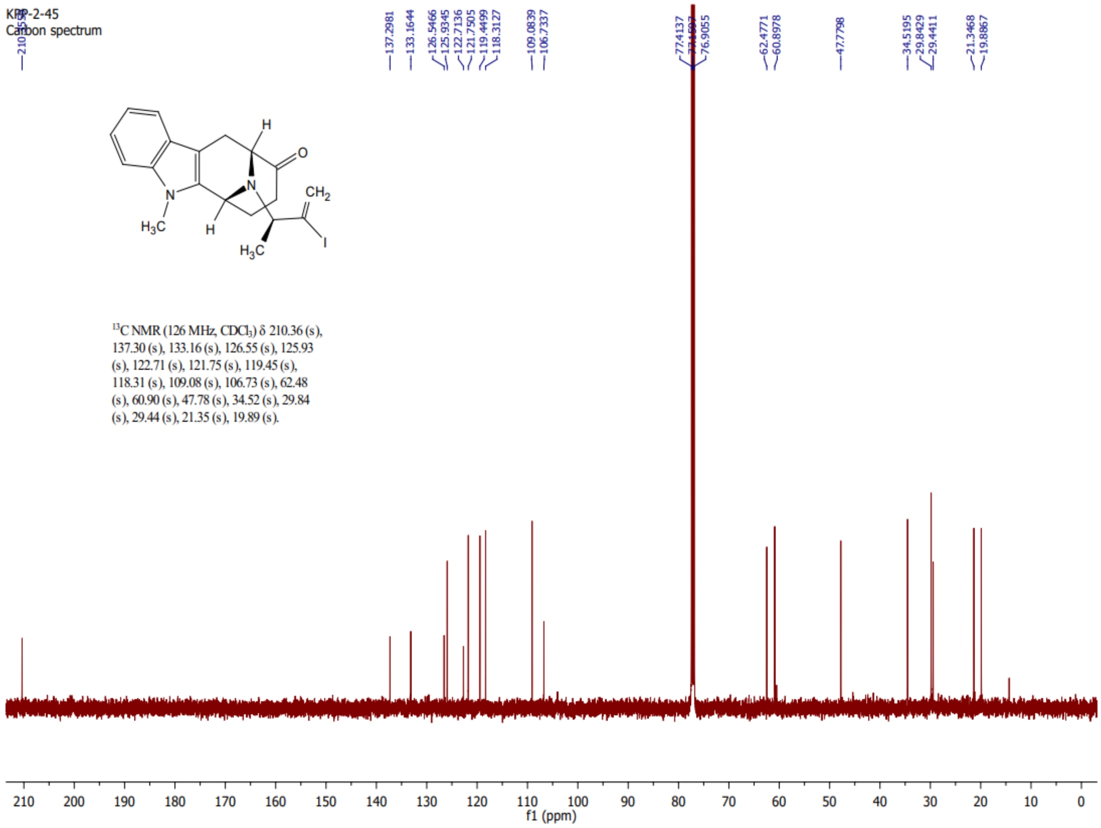

1.9. ^{1}H NMR of (6*R*,7*R*,8*R*,10*R*,11a*R*)-5,8-dimethyl-9-methylene-5,6,9,10,11a,12-hexahydro-6,10-methanoindolo[3,2-b]quinolizin-11(8H)-one (11).

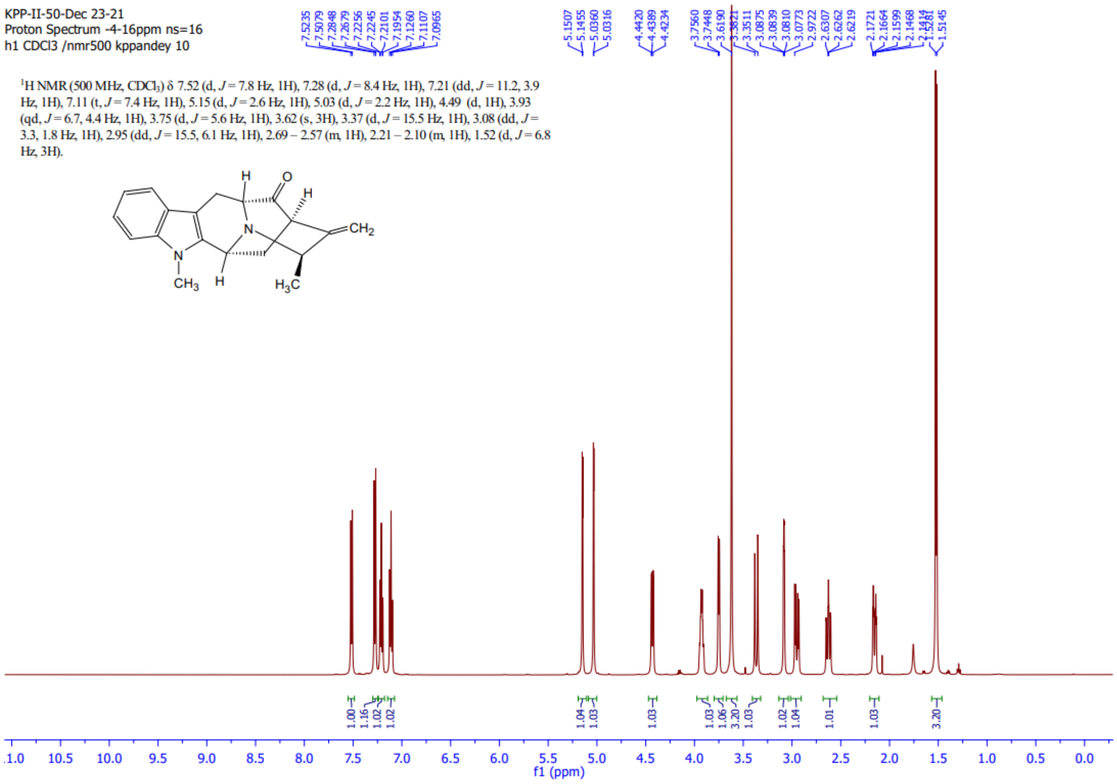

1.10. ^{13}C NMR of provide (6*R*,7*R*,8*R*,10*R*,11a*R*)-5,8-dimethyl-9-methylene-5,6,9,10,11a,12-hexahydro-6,10-methanoindolo[3,2-b]quinolizin-11(8H)-one (11).

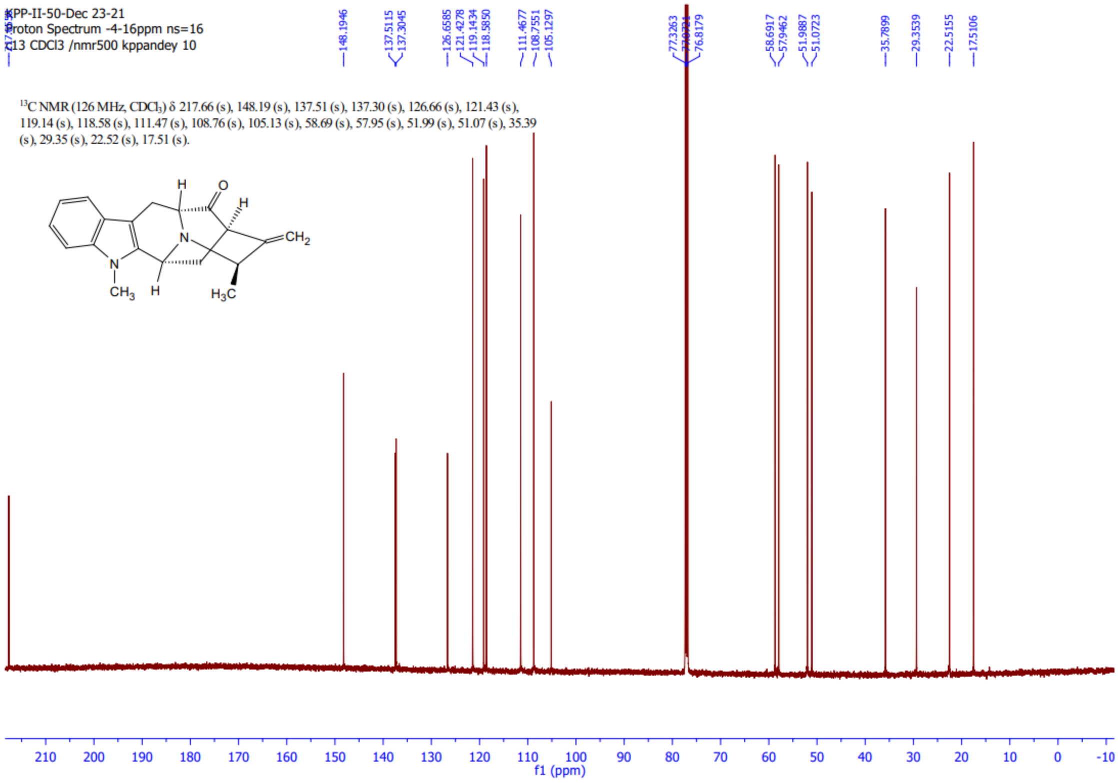

1.11. ^{1}H NMR of ((6*R*,7*R*,8*R*,10*S*,11*S*,11a*R*)-5,8-dimethyl-9-methylene-5,6,8,9,10,11,11a,12-octahydro-6,10-methanoindolo[3,2-b]quinolizin-11-yl)methanol (26).

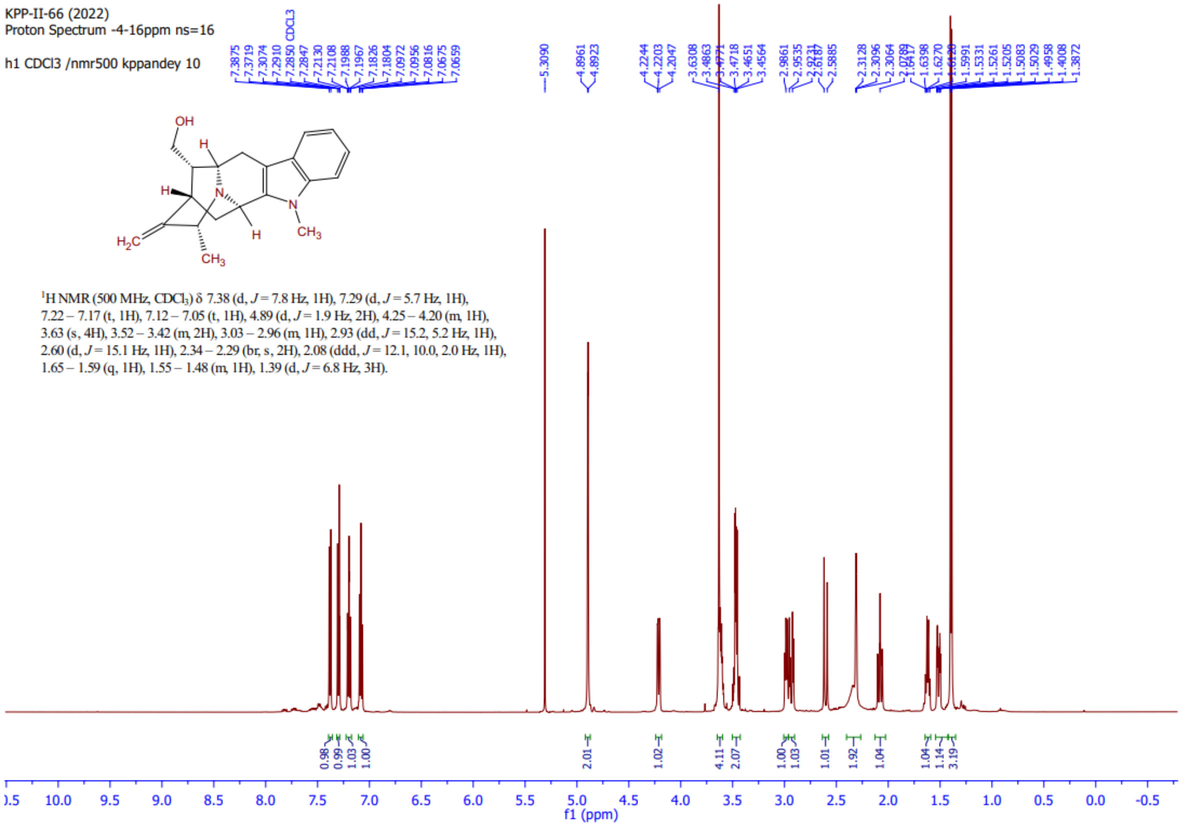

1.12. ^{13}C NMR of ((6*R*,7*R*,8*R*,10*S*,11*S*,11a*R*)-5,8-dimethyl-9-methylene-5,6,8,9,10,11,11a,12-octahydro-6,10-methanoindolo[3,2-b]quinolizin-11-yl)methanol (26).

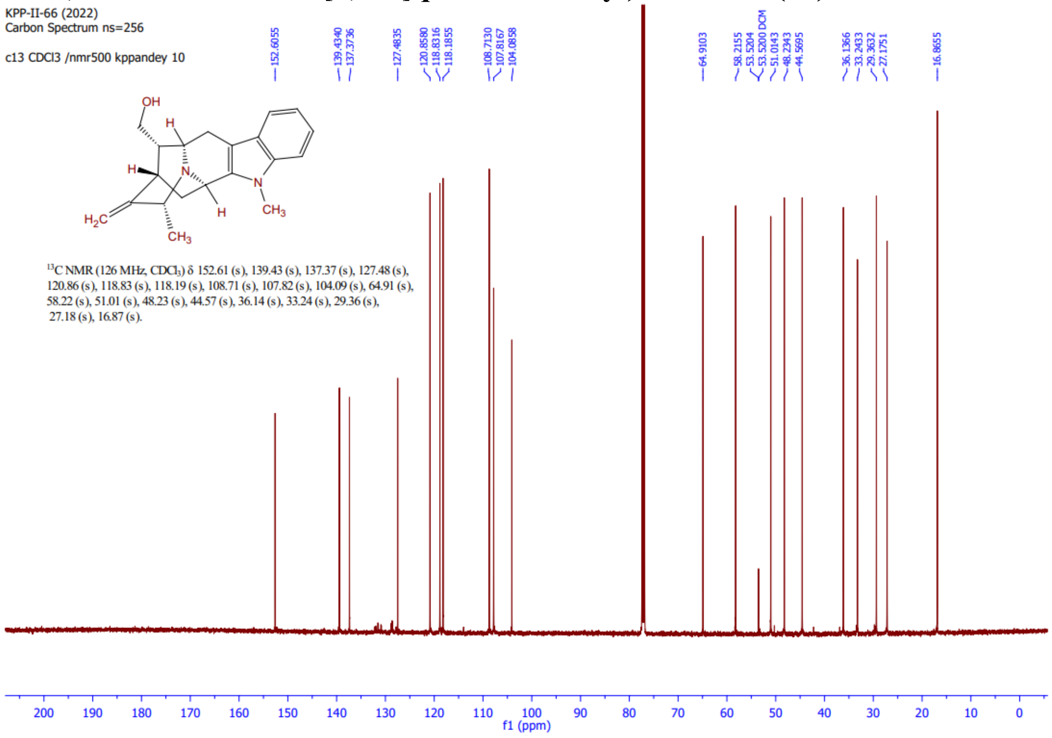

1.13. DEPT-135 ^{13}C NMR of ((6*R*,7*R*,8*R*,10*S*,11*S*,11a*R*)-5,8-dimethyl-9-methylene-5,6,8,9,10,11,11a,12-octahydro-6,10-methanoindolo[3,2-b]quinolizin-11-yl)methanol (26).

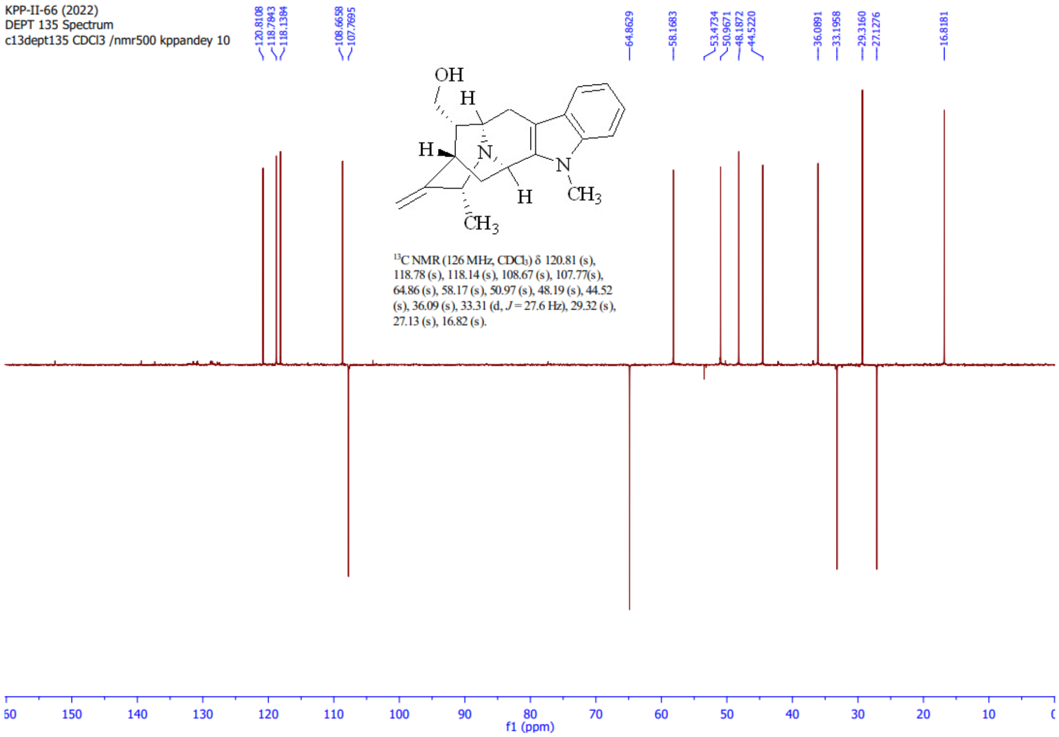

1.14. ^{1}H NMR of (6*R*,7*R*,8*R*,10*S*,11*S*,11a*R*)-5,8-dimethyl-9-methylene-11-(((triisopropylsilyl)oxy) methyl)-5,6,8,9,10,11,11a,12-octahydro-6,10-methanoindolo[3,2-b]quinolizine (27).

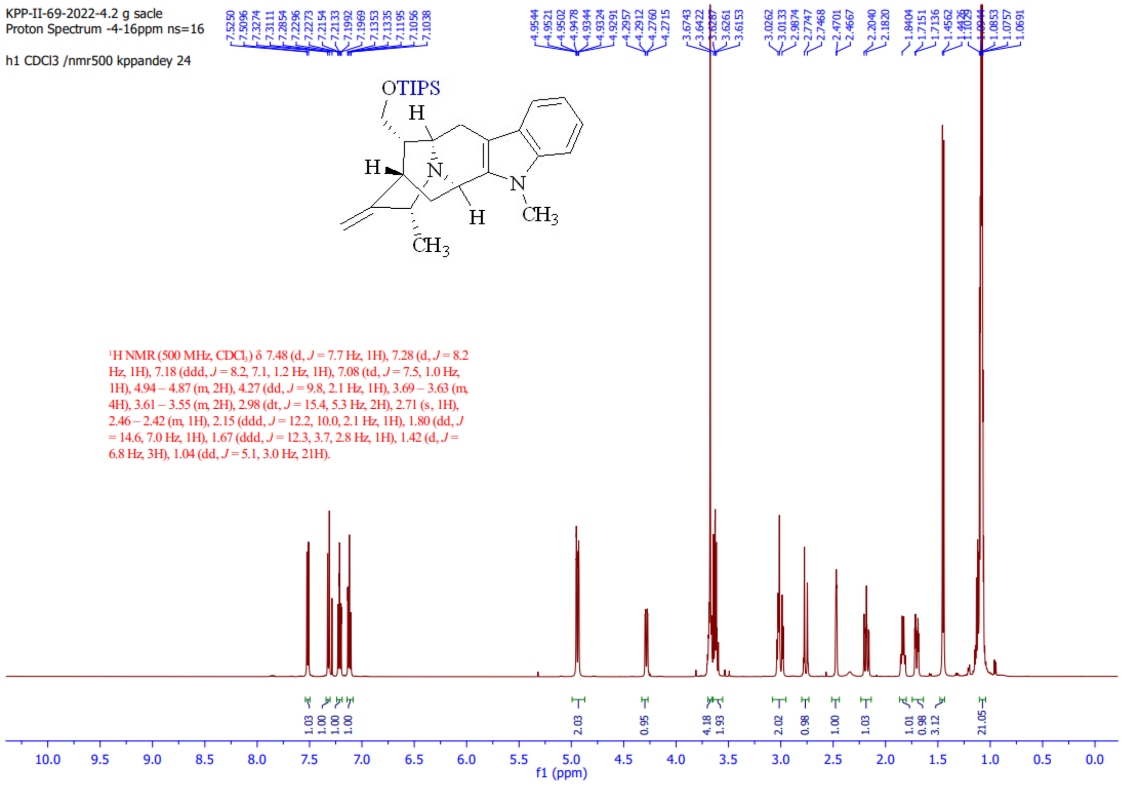

1.15. ^{13}C NMR of (6*R*,7*R*,8*R*,10*S*,11*S*,11a*R*)-5,8-dimethyl-9-methylene-11-(((triisopropylsilyl)oxy) methyl)-5,6,8,9,10,11,11a,12-octahydro-6,10-methanoindolo[3,2-b]quinolizine (27).

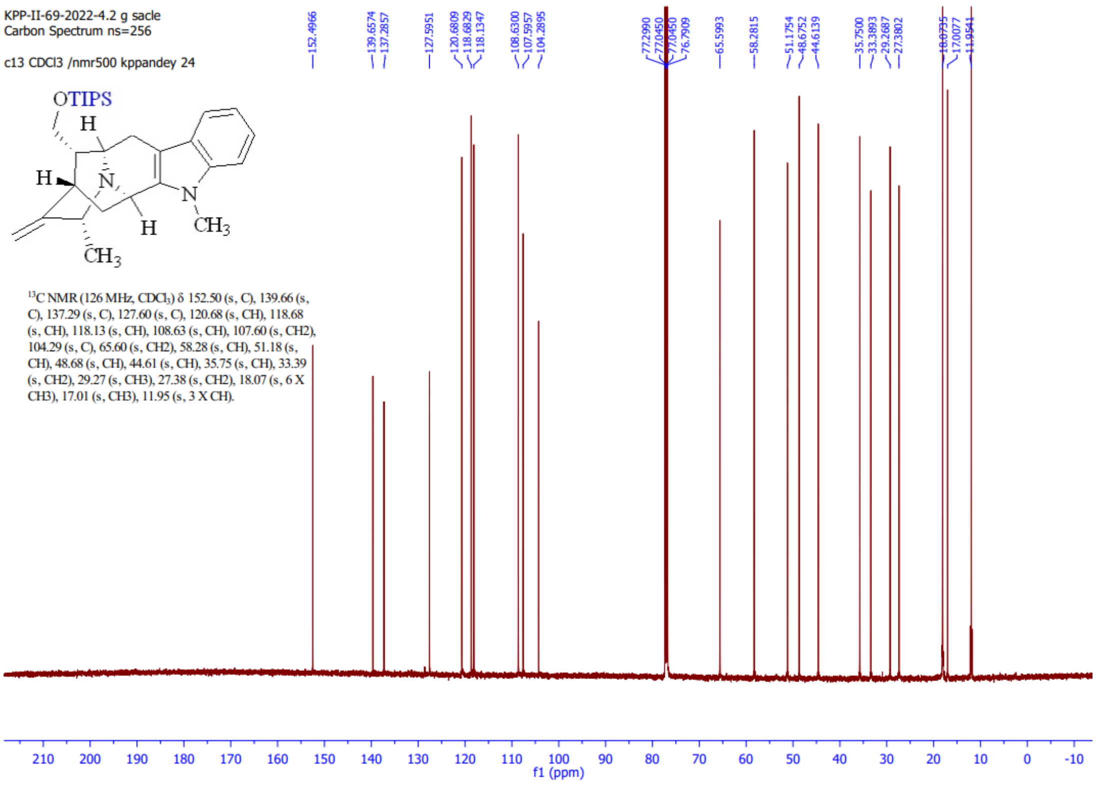

1.16. DEPT-135 ^{13}C NMR of (6*R*,7*R*,8*R*,10*S*,11*S*,11a*R*)-5,8-dimethyl-9-methylene-11-(((triisopropylsilyl)oxy) methyl)-5,6,8,9,10,11,11a,12-octahydro-6,10-methanoindolo[3,2-b]quinolizine (27).

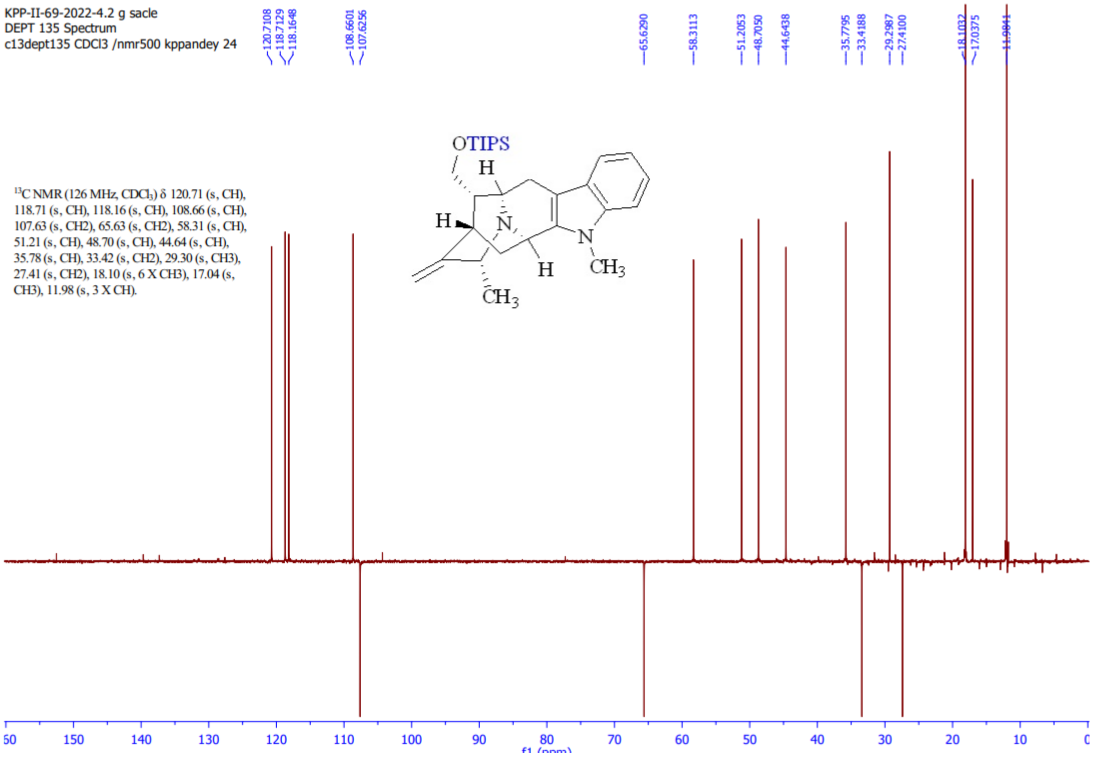

1.17. ^{1}H NMR of ((6*R*,7*S*,8*R*,9*R*,10*R*,11*S*,11a*R*)-5,8-dimethyl-11-(((triisopropylsilyl)oxy)methyl)-5,6,8,9,10,11,11a,12-octahydro-6,10-methanoindolo[3,2-b]quinolizin-9-yl)methanol (28).

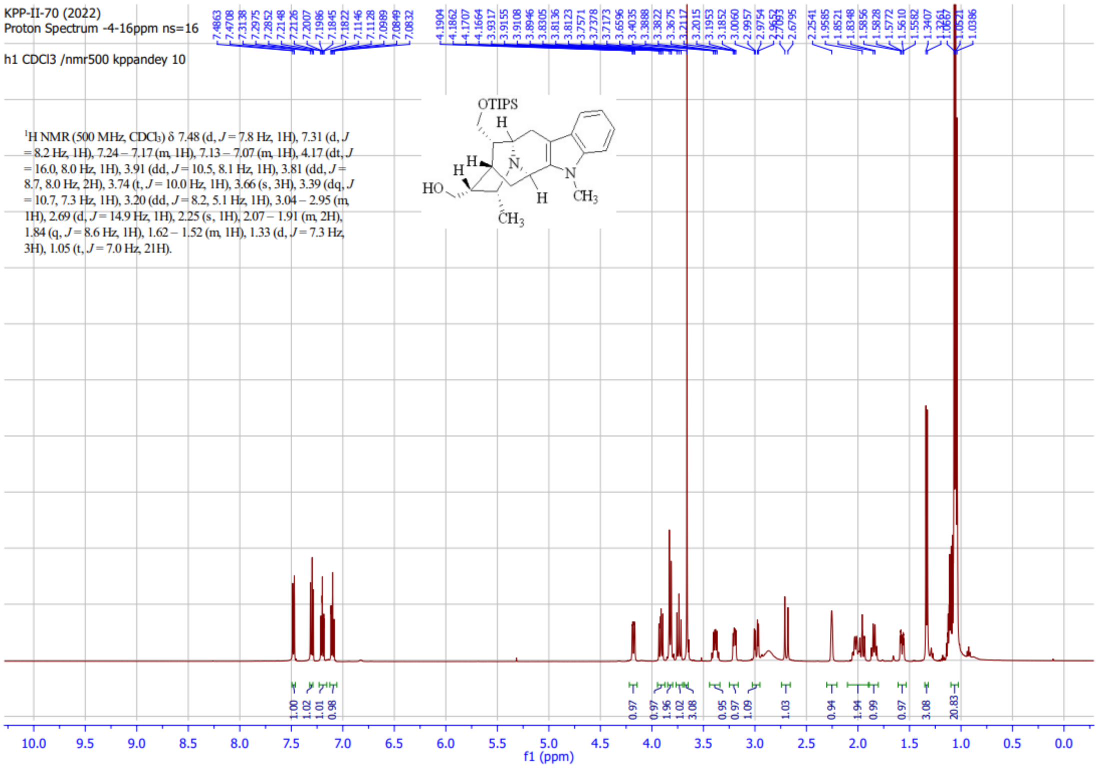

1.18. ^{13}C NMR of ((6R,7S,8R,9R,10R,11S,11aR)-5,8-dimethyl-11-(((triisopropylsilyl)oxy)methyl)-5,6,8,9,10,11,11a,12-octahydro-6,10-methanoindolo[3,2-b]quinolizin-9-yl)methanol (28).

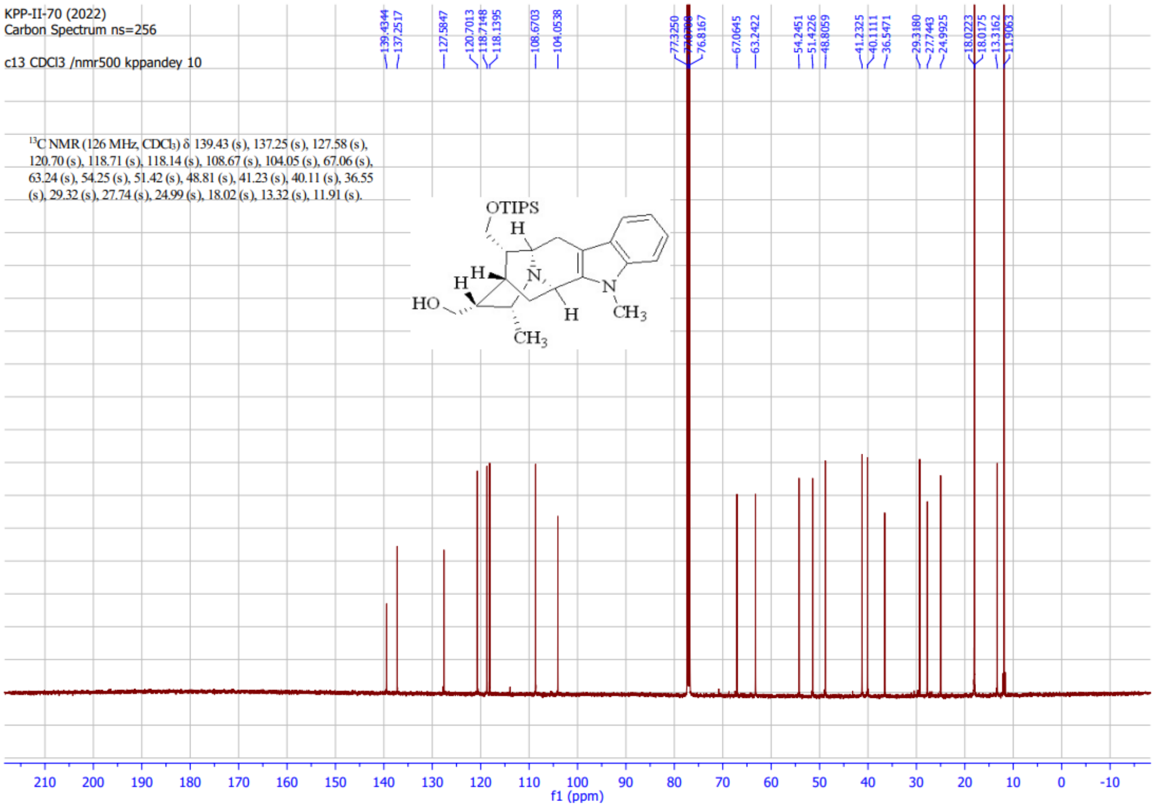

1.19. DEPT-135 ^{13}C NMR of ((6R,7S,8R,9R,10R,11S,11aR)-5,8-dimethyl-11-(((triisopropylsilyl)oxy)methyl)-5,6,8,9,10,11,11a,12-octahydro-6,10-methanoindolo[3,2-b]quinolizin-9-yl)methanol (28).

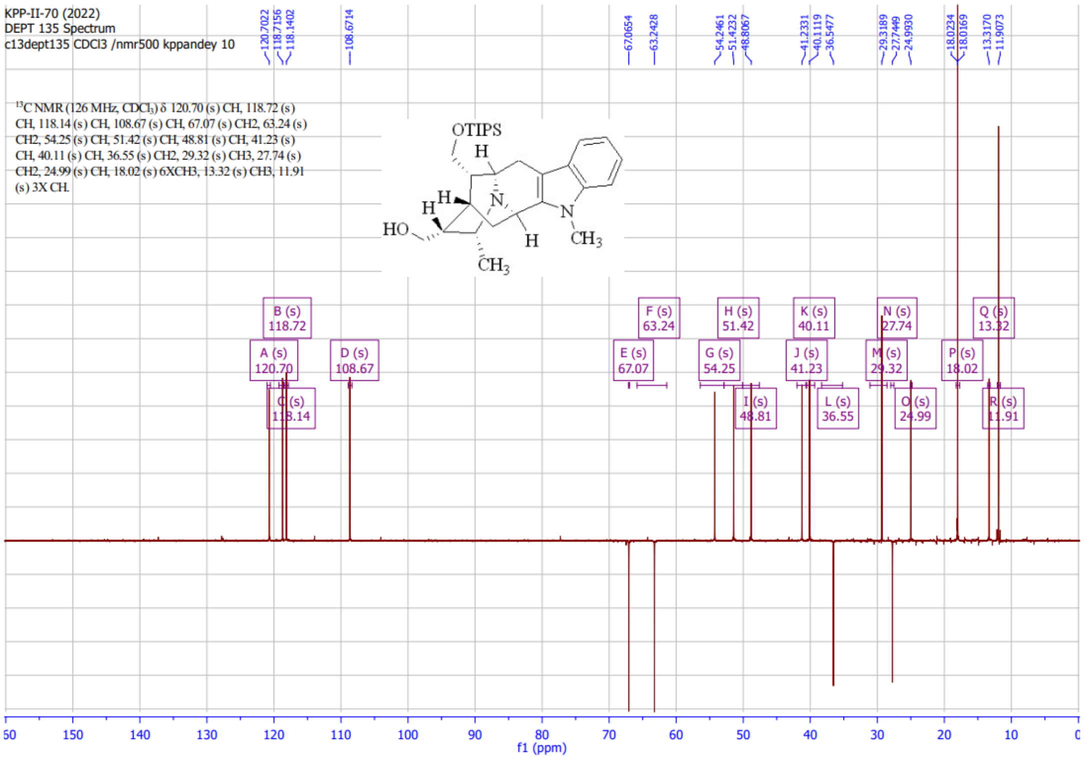

1.20. ^{1}H NMR of 6R,7S,8R,9R,10S,11S,11aR)-5,8-dimethyl-11-(((triisopropylsilyl)oxy) methyl)-5,6,8,9,10,11,11a,12-octahydro-6,10-methanoindolo[3,2-b]quinolizine-9-carbaldehyde (29).

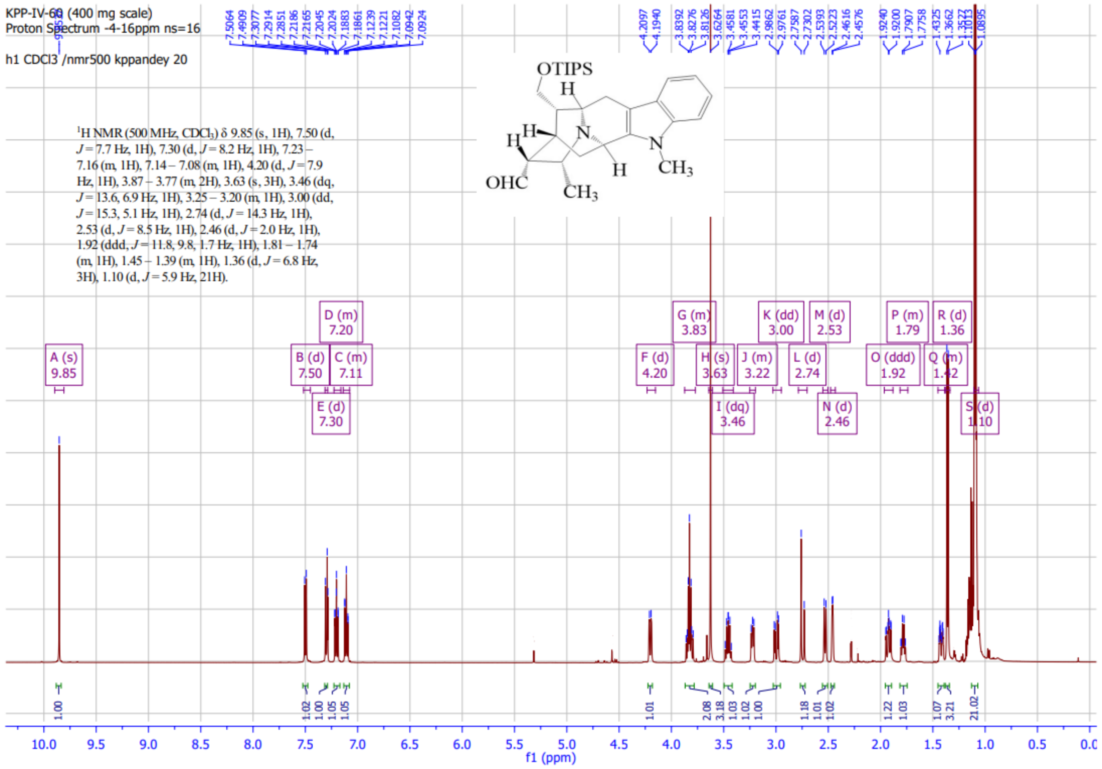

1.21. ^{13}C NMR of 6R,7S,8R,9R,10S,11S,11aR)-5,8-dimethyl-11-(((triisopropylsilyl)oxy)methyl)-5,6,8,9,10,11,11a,12-octahydro-6,10-methanoindolo[3,2-b]quinolizine-9-carbaldehyde (29).

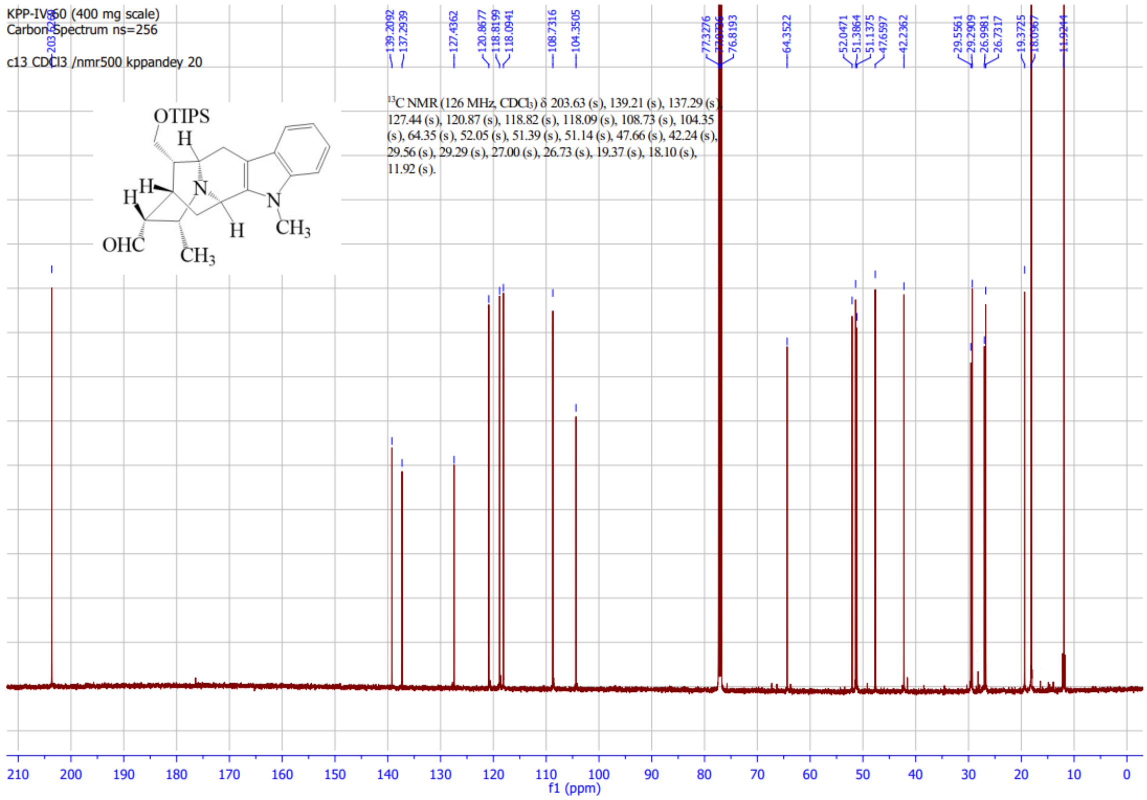

1.22. DEPT-135 ^{13}C NMR of 6R,7S,8R,9R,10S,11S,11aR)-5,8-dimethyl-11-(((triisopropylsilyl)oxy) methyl)-5,6,8,9,10,11,11a,12-octahydro-6,10-methanoindolo[3,2-b]quinolizine-9-carbaldehyde (29).

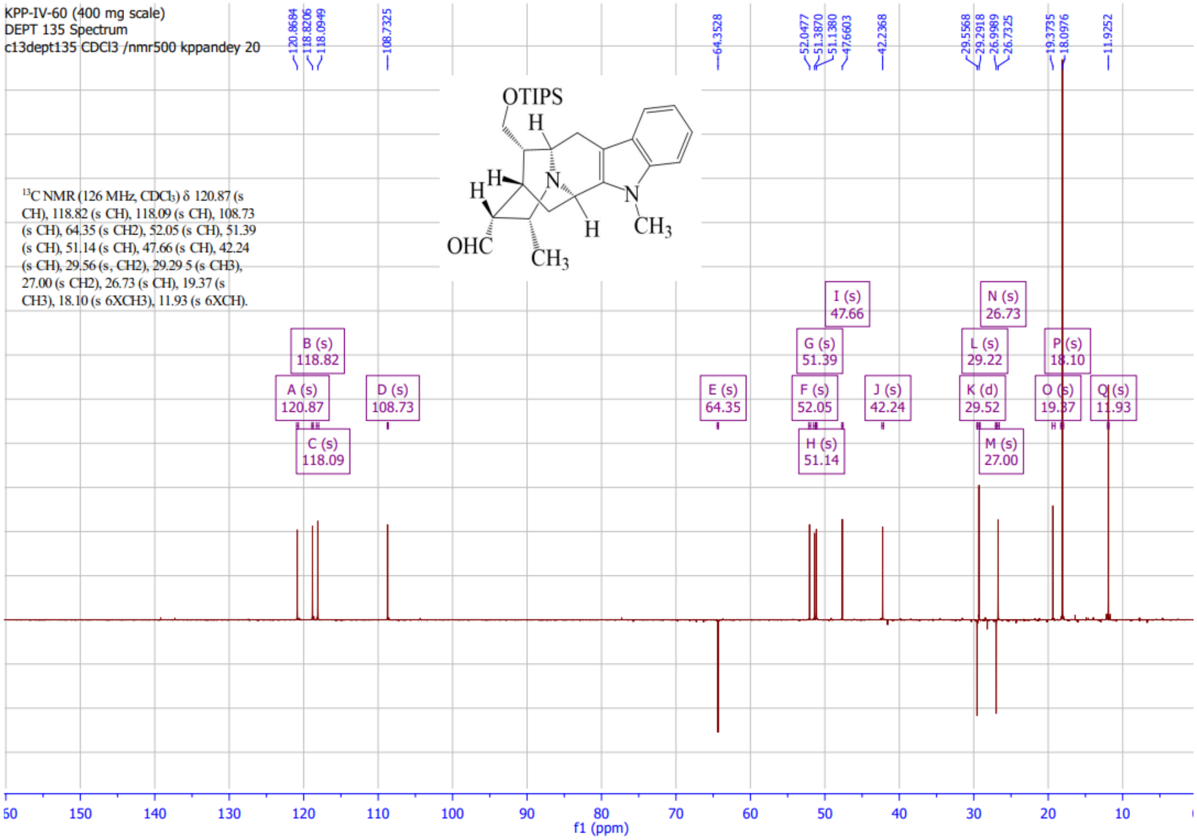

1.24. ^{1}H NMR spectra of (+) -talcarpine (1).

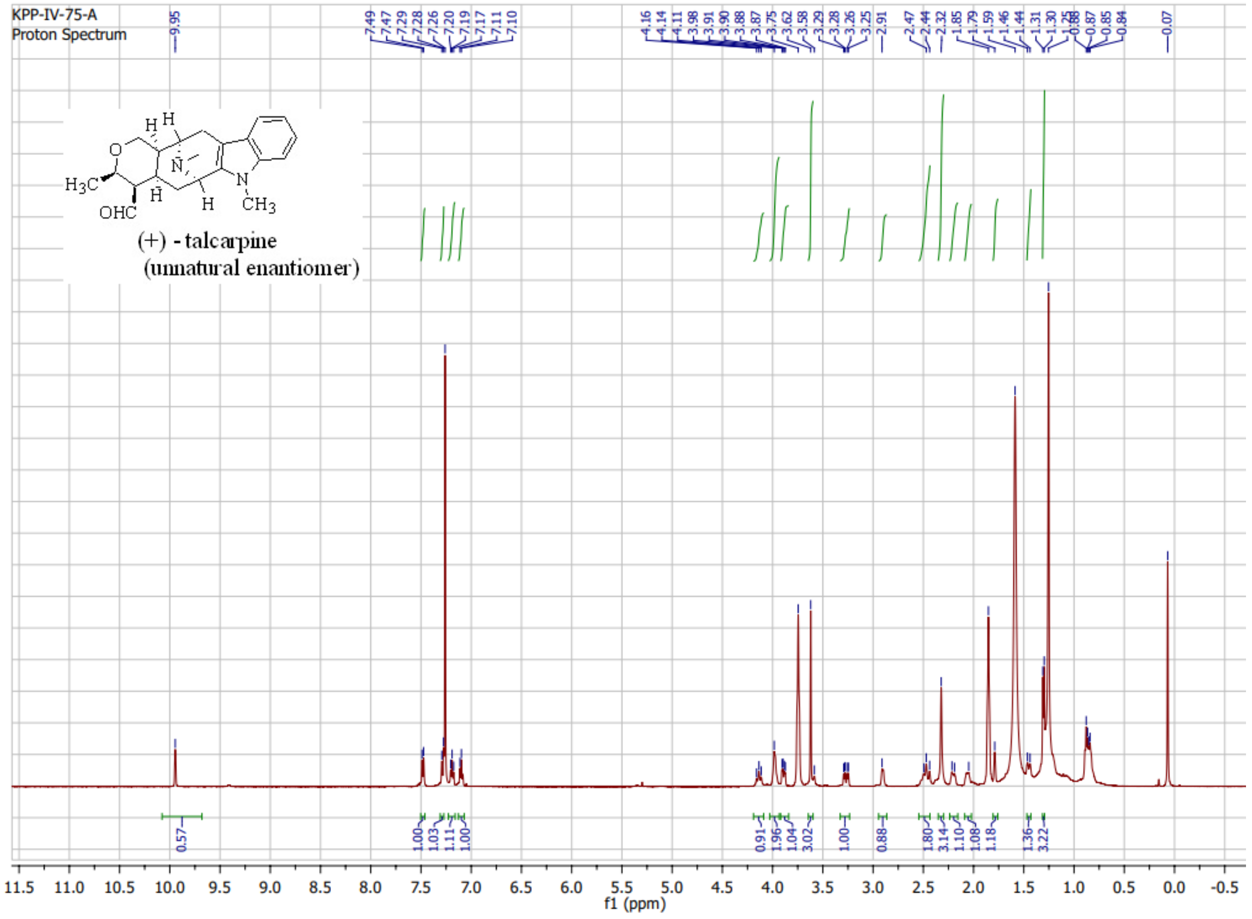

1.25. ^{13}C NMR spectra of (+) -talcarpine (1).

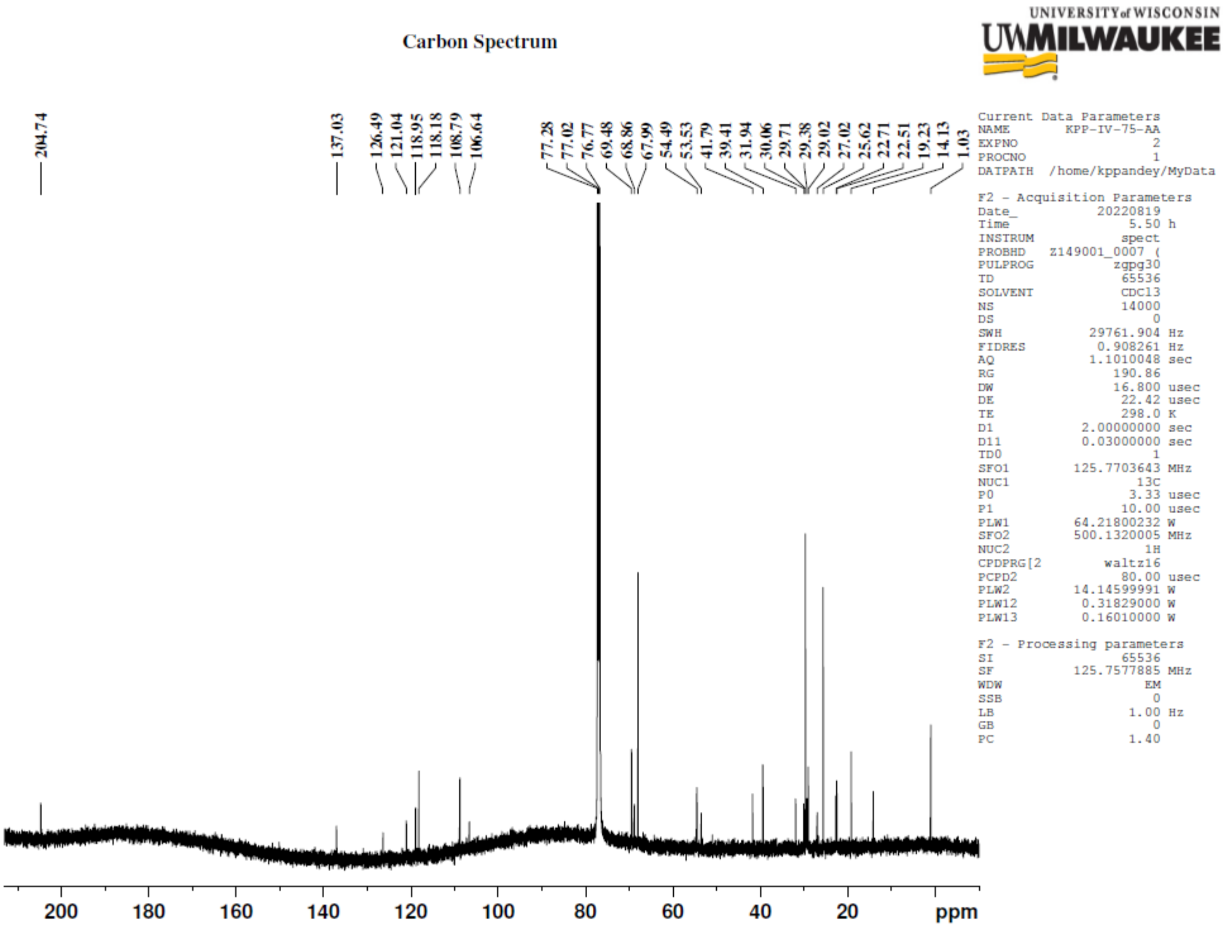

1.26. ^{1}H-^{1}H COSY spectra of (+) -talcarpine (1)

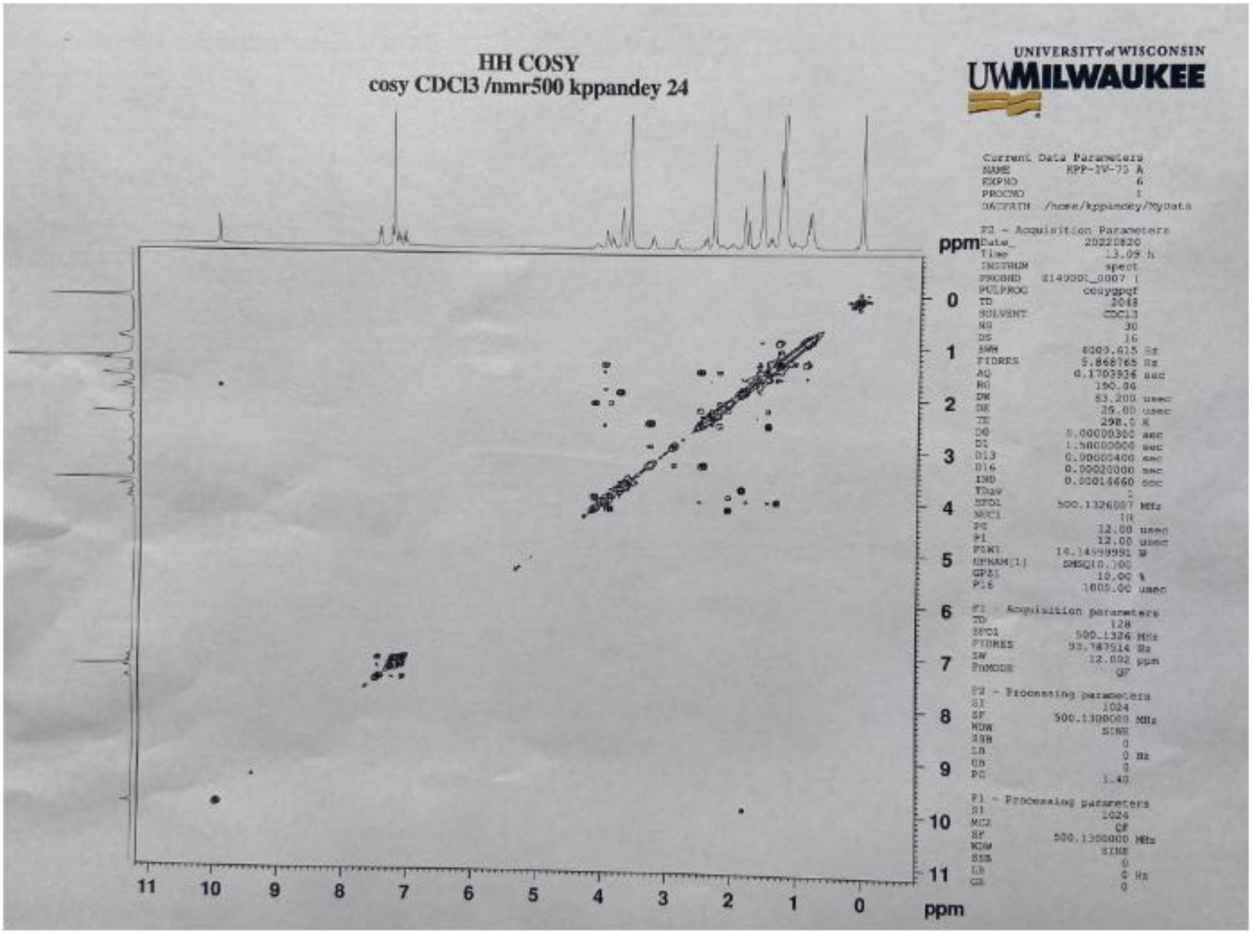

HRMS and LCMS spectra (data)

2.1. HRMS analysis of (6R,10R)-12-((R)-but-3-yn-2-yl)-5-methyl-5,6,7,8,10,11-hexahydro-9H-6,10-epiminocycloocta[b]indol-9-one (4).

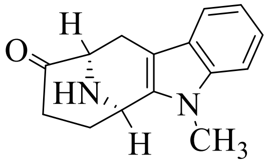

4

Formula Predictor Report - kpp-ii-43_Flowscan 700_6.lcd Page 1 of 1

Data File: C:\LabSolutions\Data\AB\MU\02-19-2019\kpp-ii-43_Flowscan 700_6.lcd

Elmt	Val.	Min	Max	Elmt	Val.	Min	Max	Elmt	Val.	Min	Max	Elmt	Val.	Min	Max	Use Adduct
H	1	15	25	13C	4	0	0	Si	4	0	0	Pb	2	0	0	H
2H	1	0	0	N	3	0	3	S	2	0	2					Na
B	3	0	0	O	2	0	6	Cl	1	0	2					NH4
C	4	18	22	F	1	0	1	Br	1	0	0					

Error Margin (ppm): 1000
HC Ratio: unlimited
Max Isotopes: all
MSn Iso RI (%): 75.00

DBE Range: -2.0 - 1000.0
Apply N Rule: yes
Isotope RI (%): 1.00
MSn Logic Mode: AND

Electron Ions: both
Use MSn Info: no
Isotope Res: 10000
Max Results: 10

Event#: 1 MS(E+) Ret. Time : 0.620 -> 0.647 - 0.727 -> 0.731 Scan# : 187 -> 195 - 219 -> 221

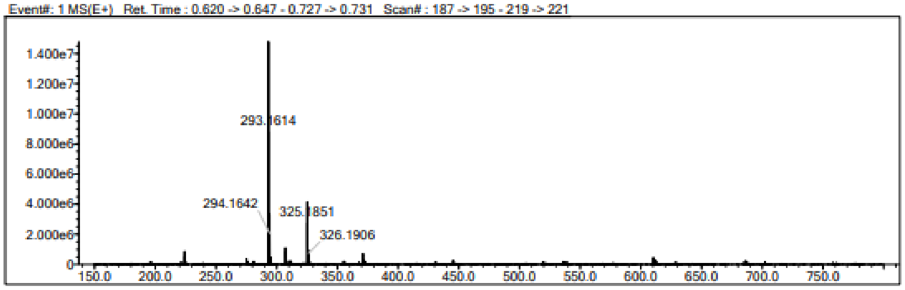

Measured region for 293.1614 m/z

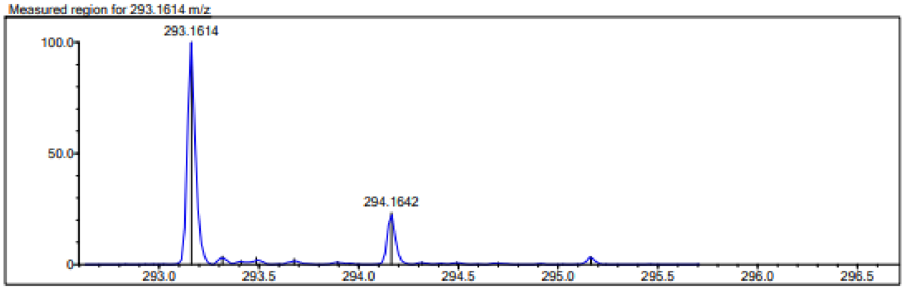

C19 H20 N2 O [M+H]+ : Predicted region for 293.1648 m/z

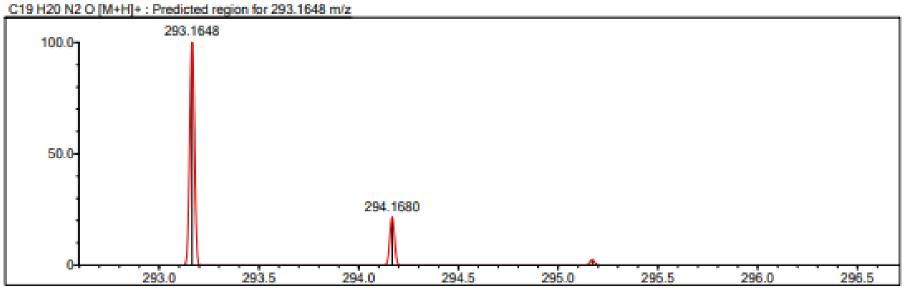

Rank	Score	Formula (M)	Ion	Meas. m/z	Pred. m/z	Df. (mDa)	Df. (ppm)	Iso	DBE
1	22.81	C19 H20 N2 O	[M+H]+	293.1614	293.1648	-3.4	-11.60	63.83	11.0

2.2. HRMS analysis of (6*R*,10*R*)-12-benzyl-5-methyl-5,6,7,8,10,11-hexahydro-9*H*-6,10-epiminocycloocta[b]indol-9-one (15).

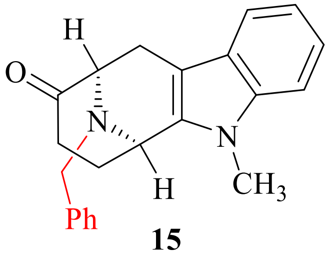

15

Formula Predictor Report - kpp-10100_Flowscan 700_2.lcd　　Page 1 of 1

Data File: C:\LabSolutions\Data\AB\MU\02-19-2019\kpp-10100_Flowscan 700_2.lcd

Elmt	Val.	Min	Max	Elmt	Val.	Min	Max	Elmt	Val.	Min	Max	Elmt	Val.	Min	Max	Use Adduct
H	1	15	25	C	4	15	25	O	2	0	3	Cl	1	0	0	H
2H	1	0	0	13C	4	0	0	Si	4	0	2	Br	1	0	0	Na
B	3	0	0	N	3	0	3	S	2	0	0	Pb	2	0	0	NH4

Error Margin (ppm):	1000	DBE Range:	-2.0 - 1000.0	Electron Ions:	both
HC Ratio:	unlimited	Apply N Rule:	yes	Use MSn Info:	no
Max Isotopes:	all	Isotope RI (%):	1.00	Isotope Res:	10000
MSn Iso RI (%):	75.00	MSn Logic Mode:	AND	Max Results:	10

Event#: 1 MS(E+)　Ret. Time : 0.493 -> 0.520 - 0.360 -> 0.360　Scan# : 149 -> 157 - 109 -> 109

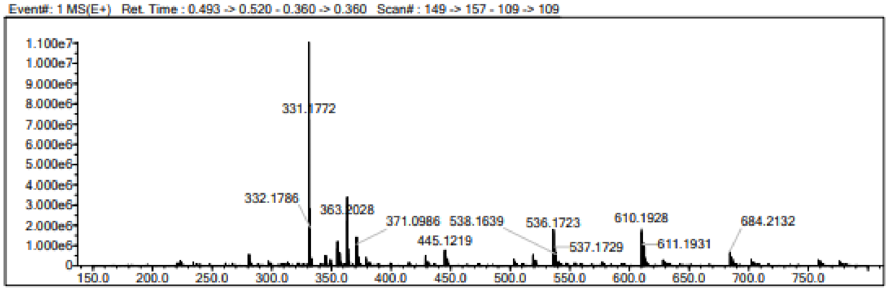

Measured region for 331.1772 m/z

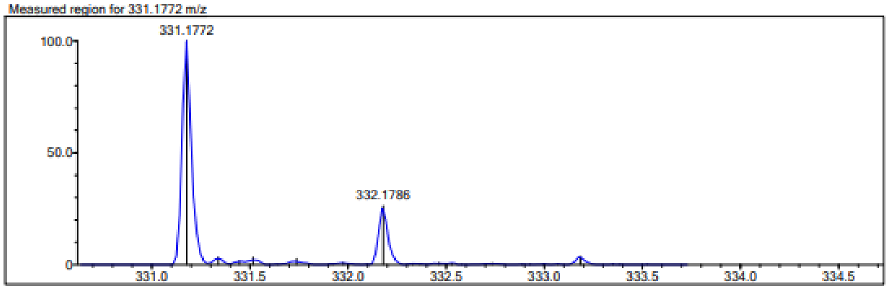

C22 H22 N2 O [M+H]+ : Predicted region for 331.1805 m/z

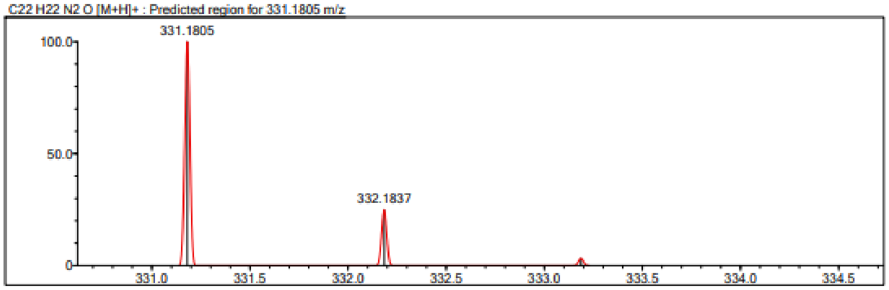

Rank	Score	Formula (M)	Ion	Meas. m/z	Pred. m/z	Df. (mDa)	Df. (ppm)	Iso	DBE
5	28.30	C22 H22 N2 O	[M+H]+	331.1772	331.1805	-3.3	-9.96	70.05	13.0

2.3. HRMS analysis of ((6*R*,7*R*,8*R*,10*S*,11*S*,11a*R*)-5,8-dimethyl-9-methylene-5,6,8,9,10,11,11a,12-octahydro-6,10-methanoindolo[3,2-b]quinolizin-11-yl)methanol (26).

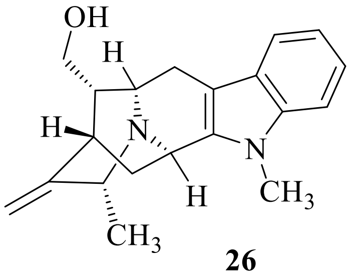

26

Formula Predictor Report - kpp0-ii=66_Flowscan 700_8.lcd — Page 1 of 1

Data File: C:\LabSolutions\Data\AB\MU\02-19-2019\kpp0-ii=66_Flowscan 700_8.lcd

Elmt	Val.	Min	Max	Elmt	Val.	Min	Max	Elmt	Val.	Min	Max	Elmt	Val.	Min	Max	Use Adduct
H	1	15	25	13C	4	0	0	Si	4	0	0	Pb	2	0	0	H
2H	1	0	0	N	3	0	3	S	2	0	2					Na
B	3	0	0	O	2	0	6	Cl	1	0	2					NH4
C	4	18	22	F	1	0	1	Br	1	0	0					

Error Margin (ppm): 1000
HC Ratio: unlimited
Max Isotopes: all
MSn Iso RI (%): 75.00

DBE Range: -2.0 - 1000.0
Apply N Rule: yes
Isotope RI (%): 1.00
MSn Logic Mode: AND

Electron Ions: both
Use MSn Info: no
Isotope Res: 10000
Max Results: 10

Event#: 1 MS(E+) Ret. Time : 0.460 -> 0.507 - 0.760 -> 0.761 Scan# : 139 -> 153 - 229 -> 229

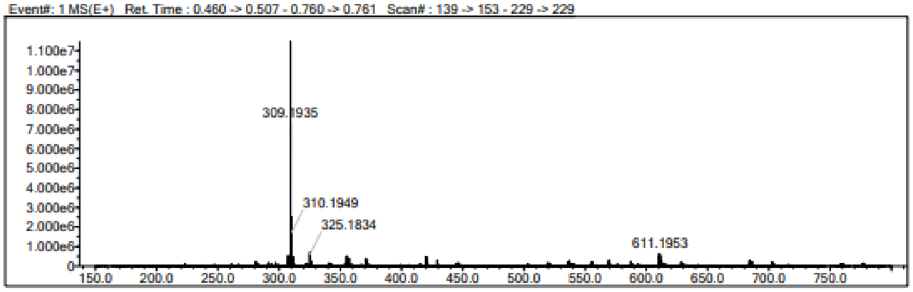

Measured region for 309.1935 m/z

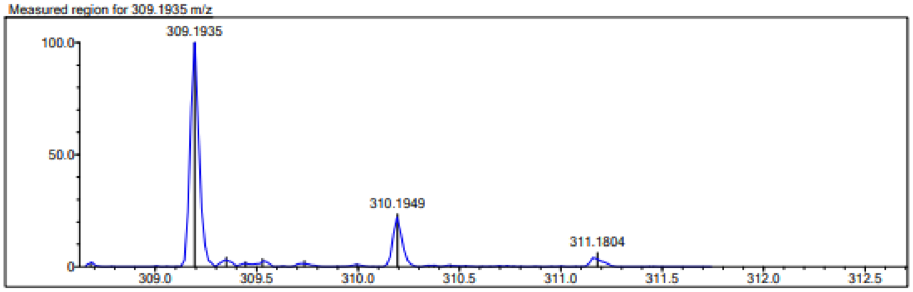

C20 H24 N2 O [M+H]+ : Predicted region for 309.1961 m/z

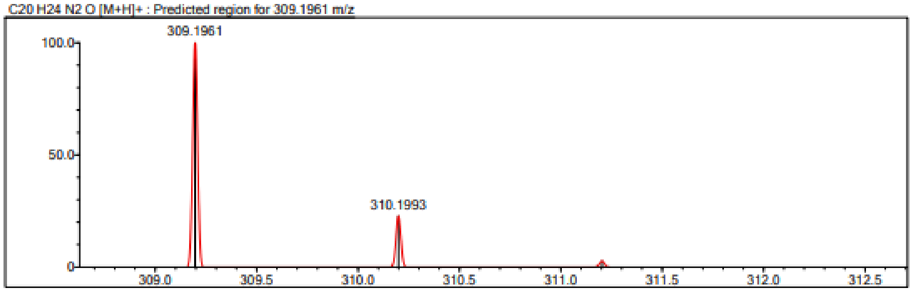

Rank	Score	Formula (M)	Ion	Meas. m/z	Pred. m/z	Df. (mDa)	Df. (ppm)	Iso	DBE
1	29.46	C20 H24 N2 O	[M+H]+	309.1935	309.1961	-2.6	-8.41	52.70	10.0

2.4. HRMS analysis of (6*R*,7*R*,8*R*,10*S*,11*S*,11a*R*)-5,8-dimethyl-9-methylene-11-(((triisopropylsilyl)oxy) methyl)-5,6,8,9,10,11,11a,12-octahydro-6,10-methanoindolo[3,2-b]quinolizine (27).

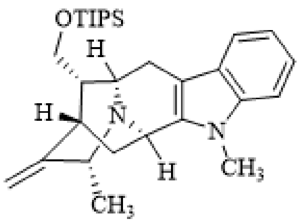

Scan positive no column.lcm

Formula Predictor Result	**C29 H44 N2 O Si**
Mass	465.32999
Error Margin	10 ppm
DBE Range	0 - 1000
Electron Ions	Both configurations
HC Ratio	Not Used
Nitrogen Rule	Used

Score	Pred. (M)	Pred. m/z	Meas. m/z	Diff. (mDa)	Formulae (M)	Ion	Diff. (ppm)	Iso Score	DBE
91.46	464.32229	465.32957	465.32999	0.42	C29 H44 N2 O Si	[M+H]+	0.903	90.96	9.0

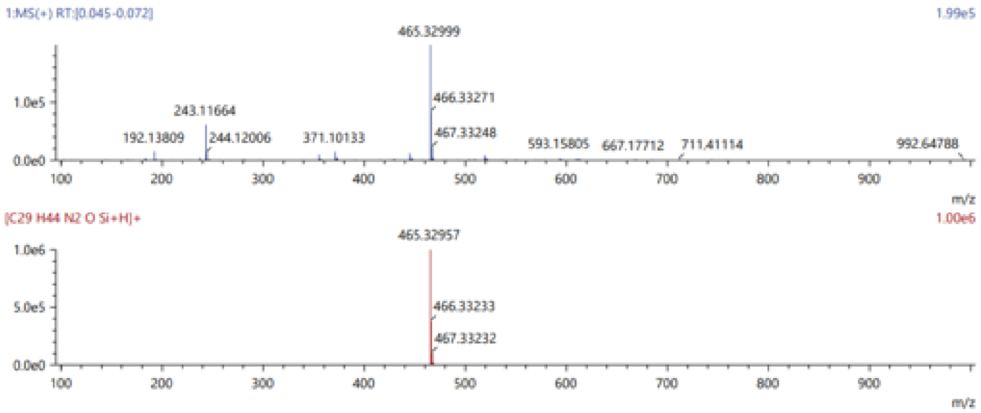

2.5. HRMS analysis of ((6*R*,7*S*,8*R*,9*R*,10*R*,11*S*,11a*R*)-5,8-dimethyl-11-(((triisopropylsilyl)oxy)methyl)-5,6,8,9,10,11,11a,12-octahydro-6,10-methanoindolo[3,2-b]quinolizin-9-yl)methanol **(28)**.

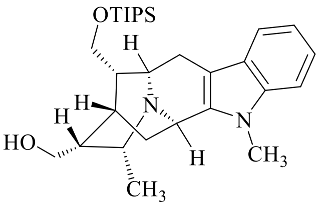

28

Formula Predictor Report - kpp-ii-70_Flowscan 700_4.lcd   Page 1 of 1

Data File: C:\LabSolutions\Data\AB\MU\02-19-2019\kpp-ii-70_Flowscan 700_4.lcd

Elmt	Val.	Min	Max	Elmt	Val.	Min	Max	Elmt	Val.	Min	Max	Elmt	Val.	Min	Max	Use Adduct
H	1	40	50	C	4	29	35	O	2	0	3	Cl	1	0	0	H
2H	1	0	0	13C	4	0	0	Si	4	0	2	Br	1	0	0	Na
B	3	0	0	N	3	0	3	S	2	0	0	Pb	2	0	0	NH4

Error Margin (ppm):	1000	DBE Range:	-2.0 - 1000.0	Electron Ions:	both
HC Ratio:	unlimited	Apply N Rule:	yes	Use MSn Info:	no
Max Isotopes:	all	Isotope RI (%):	1.00	Isotope Res:	10000
MSn Iso RI (%):	75.00	MSn Logic Mode:	AND	Max Results:	10

Event#: 1 MS(E+) Ret. Time : 0.420 -> 0.573 - 0.207 -> 0.282 Scan# : 127 -> 173 - 63 -> 85

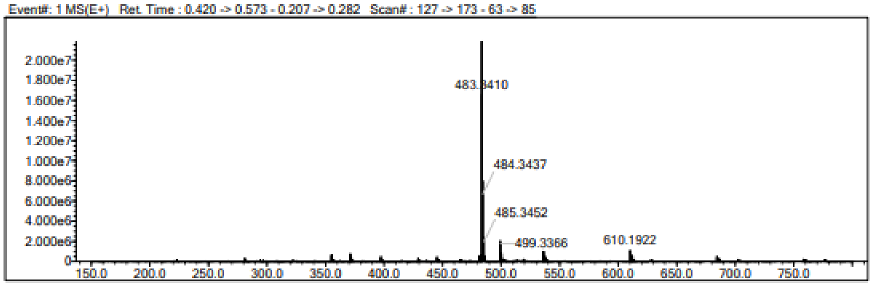

Measured region for 483.3410 m/z

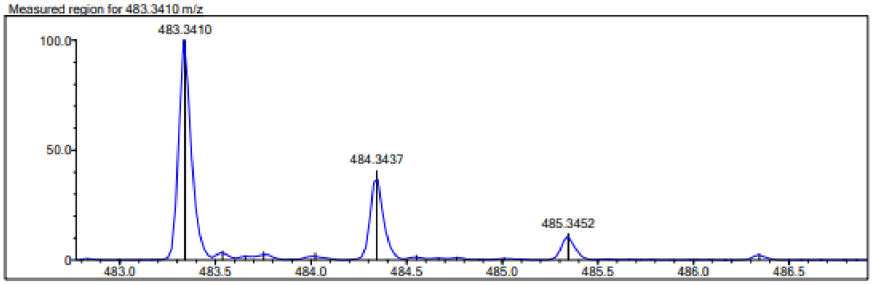

C29 H46 N2 O2 Si [M+H]+ : Predicted region for 483.3401 m/z

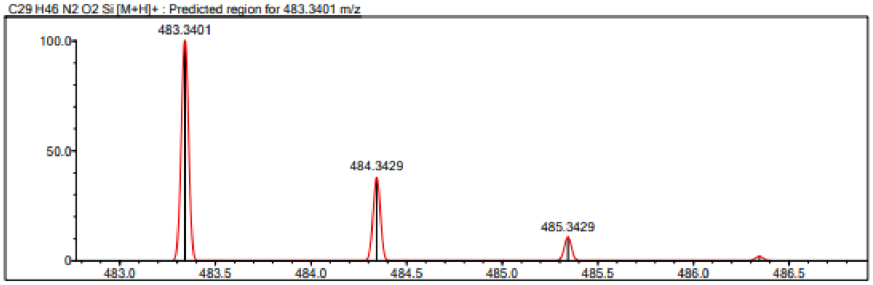

Rank	Score	Formula (M)	Ion	Meas. m/z	Pred. m/z	Df. (mDa)	Df. (ppm)	Iso	DBE
1	85.56	C29 H46 N2 O2 Si	[M+H]+	483.3410	483.3401	0.9	1.86	87.44	9.0

2.6. 6*R*,7*S*,8*R*,9*R*,10*S*,11*S*,11a*R*)-5,8-dimethyl-11-(((triisopropylsilyl)oxy) methyl)-5,6,8,9,10,11,11a,12-octahydro-6,10-methanoindolo[3,2-b]quinolizine-9-carbaldehyde **(29)**.

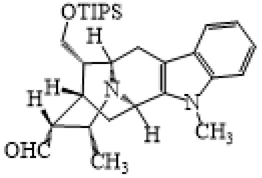

Scan positive no column.lcm

Formula Predictor Result	**C29 H44 N2 O2 Si**
Mass	481.32519
Error Margin	10 ppm
DBE Range	0 - 1000
Electron Ions	Both configurations
HC Ratio	Not Used
Nitrogen Rule	Used

Score	Pred. (M)	Pred. m/z	Meas. m/z	Diff. (mDa)	Formulae (M)	Ion	Diff. (ppm)	Iso Score	DBE
52.29	480.31721	481.32448	481.32519	0.71	C29 H44 N2 O2 Si	[M+H]+	1.475	47.80	9.0

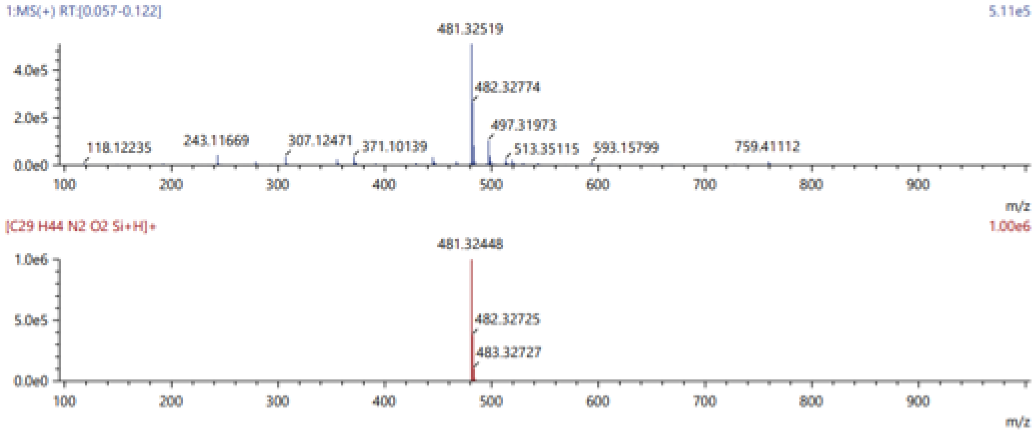

2.7. LCMS analysis of ((6*R*,7*R*,8*R*,10*S*,11*S*,11a*R*)-5,8-dimethyl-9-methylene-5,6,8,9,10,11,11a,12-octahydro-6,10-methanoindolo[3,2-b]quinolizin-11-yl)methanol (30).

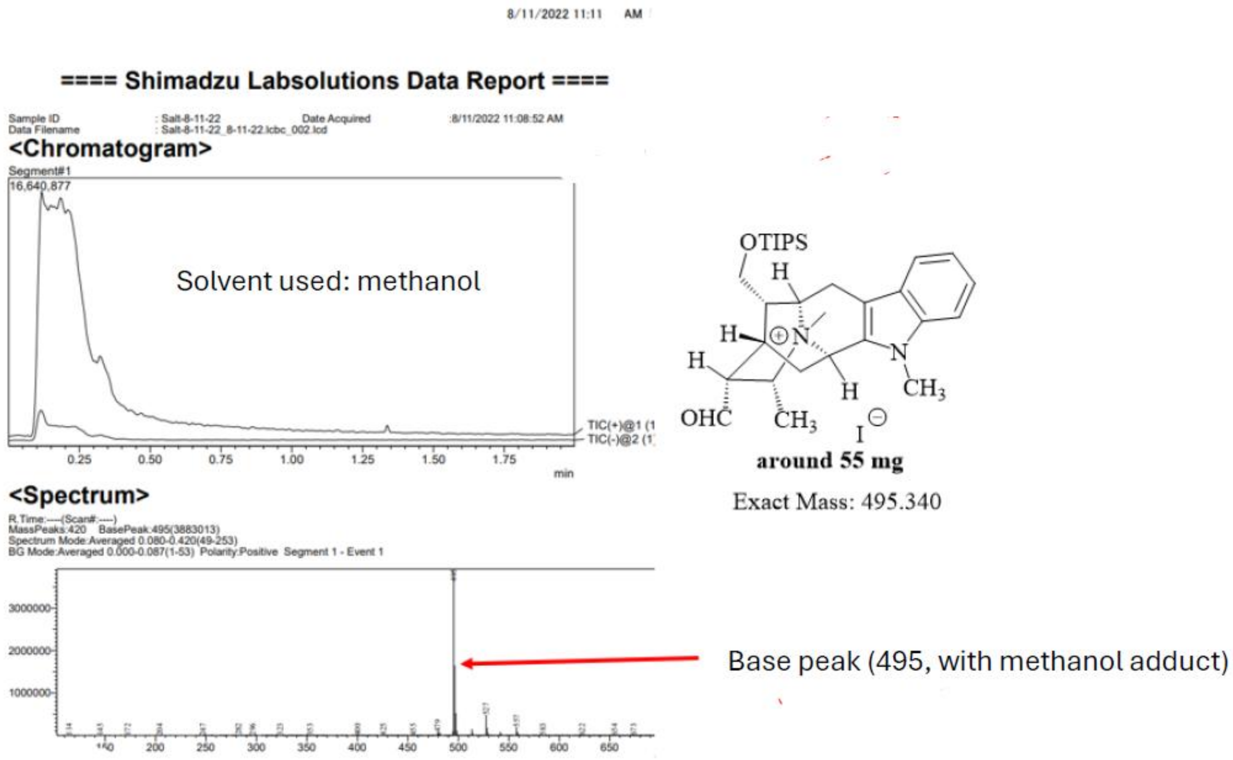

2.8. HRMS analysis of (6*R*,7*S*,8*R*,9*R*,10*S*,11*S*,11a*R*)-9-formyl-5,7,8-trimethyl-11-(((triisopropylsilyl)oxy)methyl)-6,7,8,9,10,11,11a,12-octahydro-5*H*-6,10-methanoindolo[3,2-b]quinolizin-7-ium **(30)**.

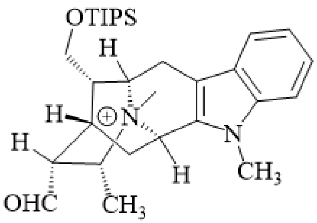

7-29-2022.lcb

Formula Predictor Result	**C30 H46 N2 O2 Si**
Mass	495.34352
Error Margin	100 ppm
DBE Range	0 - 1000
Electron Ions	Both configurations
HC Ratio	0 - 3
Nitrogen Rule	Used

Score	Pred. (M)	Pred. m/z	Meas. m/z	Diff. (mDa)	Formulae (M)	Ion	Diff. (ppm)	Iso Score	DBE
12.76	494.33286	495.34013	495.34352	3.39	C30 H46 N2 O2 Si	[M+H]+	6.844	14.17	9.0

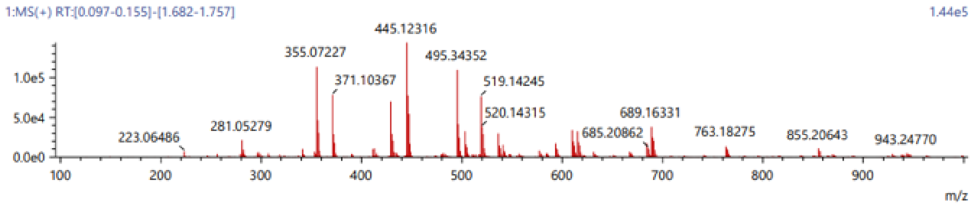

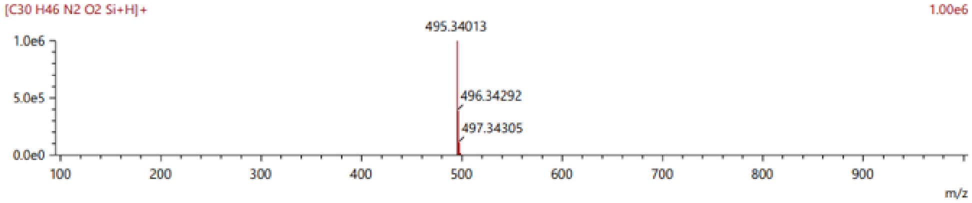

2.9. HRMS analysis of 31.

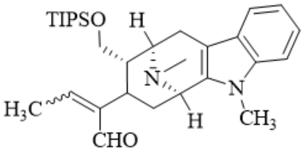

7-29-2022.lcb

Formula Predictor Result	**C30 H46 N2 O2 Si**
Mass	495.34396
Error Margin	100 ppm
DBE Range	0 - 1000
Electron Ions	Both configurations
HC Ratio	0 - 3
Nitrogen Rule	Used

Score	Pred. (M)	Pred. m/z	Meas. m/z	Diff. (mDa)	Formulae (M)	Ion	Diff. (ppm)	Iso Score	DBE
2.82	494.33286	495.34013	495.34396	3.83	C30 H46 N2 O2 Si	[M+H]+	7.732	3.13	9.0

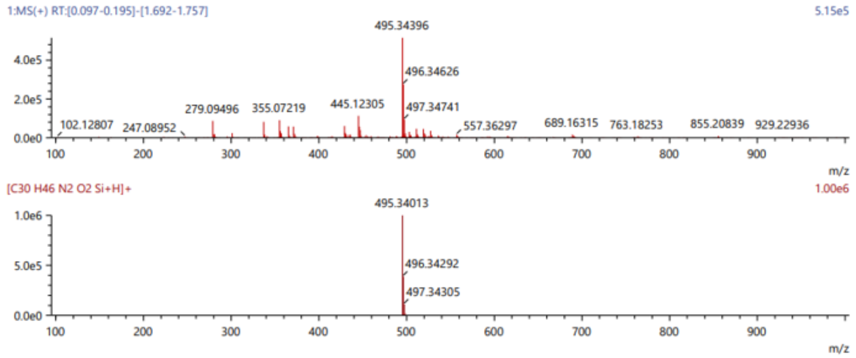

2.10. LCMS anaysis of (+)-talcarpine (1).

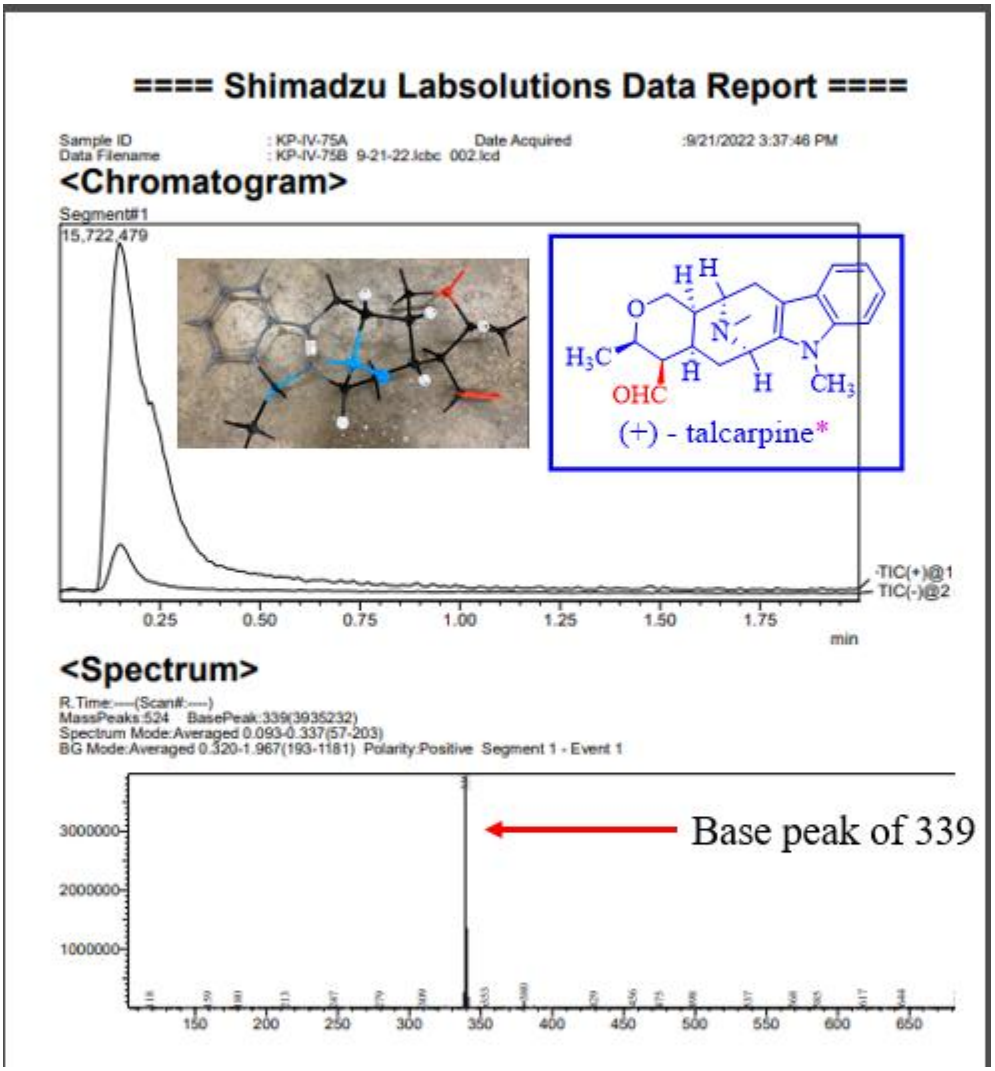

2.11. HRMS analysis of (+)-talcarpine (1).

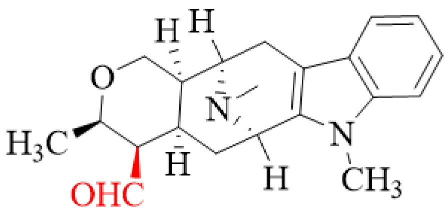

(+) - talcarpine
(unnatural enantiomer)

10-3-2022.lcb

Formula Predictor Result	**C21 H26 N2 O2**
Mass	339.20751
Error Margin	5 ppm
DBE Range	0 - 1000
Electron Ions	Both configurations
HC Ratio	0 - 3
Nitrogen Rule	Used

Score	Pred. (M)	Pred. m/z	Meas. m/z	Diff. (mDa)	Formulae (M)	Ion	Diff. (ppm)	Iso Score	DBE
54.62	338.19943	339.20670	339.20751	0.81	C21 H26 N2 O2	[M+H]+	2.388	51.10	10.0

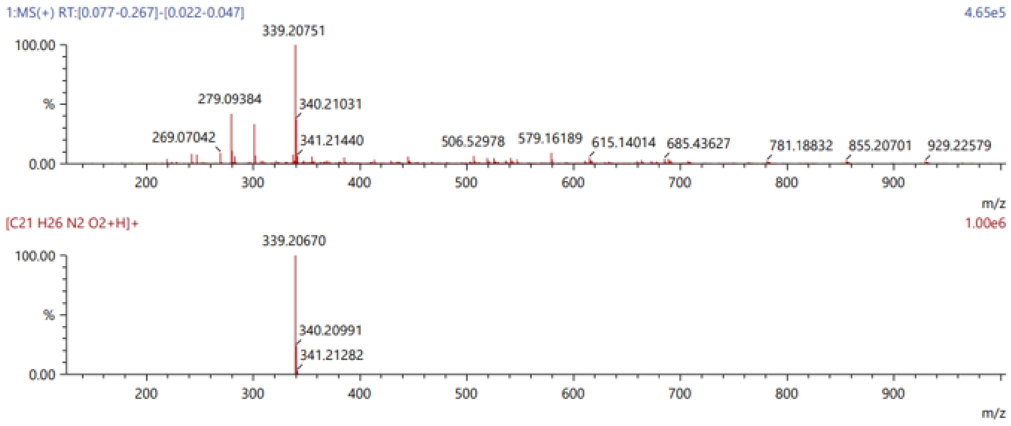

2.12. LCMS anaysis of (-) N_4-methyl, N_4-21-secotalpinine **(2)**.

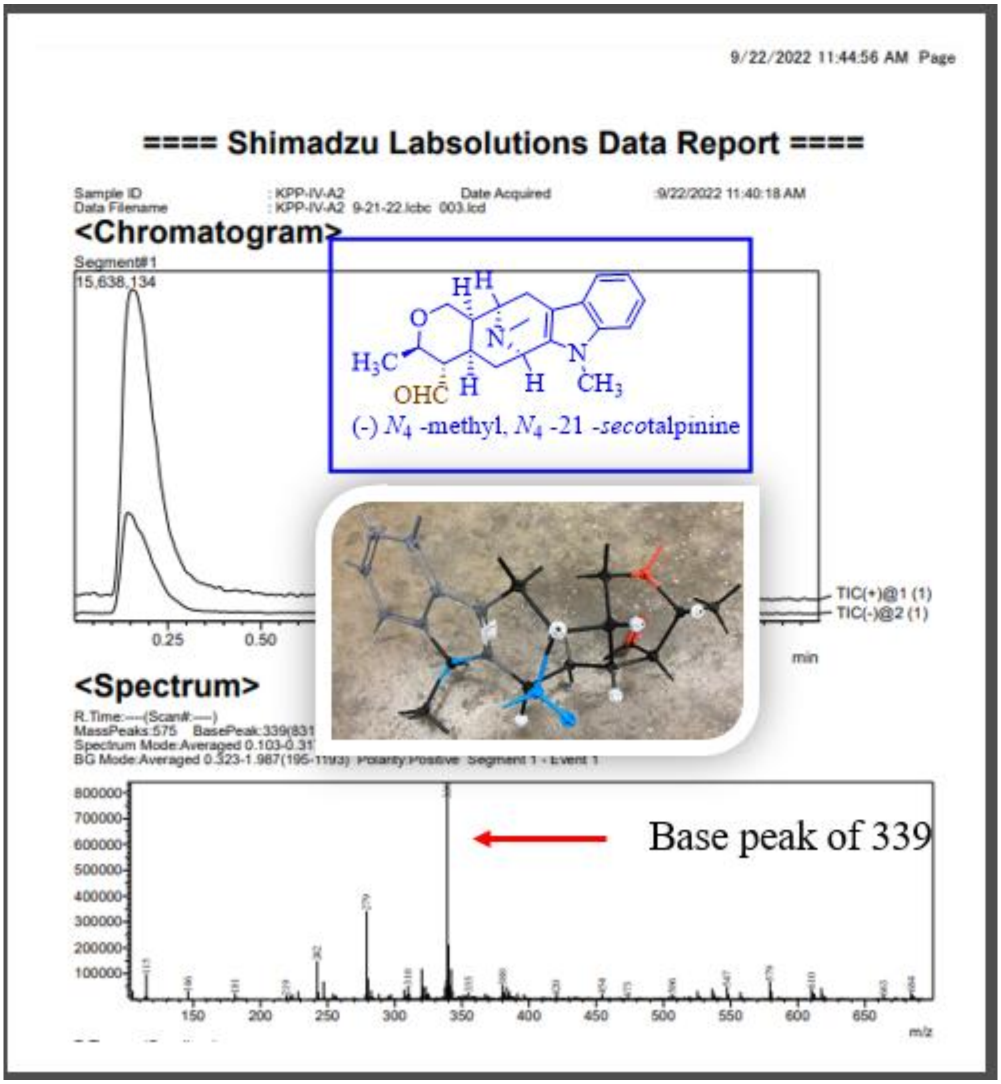

2.13. HRMS analysis of (-)-N_4-methyl, N_4-21-secotalpinine **2**.

10-3-2022.lcb

Formula Predictor Result	**C21 H26 N2 O2**
Mass	339.2074
Error Margin	5 ppm
DBE Range	0 - 1000
Electron Ions	Both configurations
HC Ratio	0 - 3
Nitrogen Rule	Used

Score	Pred. (M)	Pred. m/z	Meas. m/z	Diff. (mDa)	Formulae (M)	Ion	Diff. (ppm)	Iso Score	DBE
55.19	338.19943	339.20670	339.20740	0.70	C21 H26 N2 O2	[M+H]+	2.064	51.45	10.0

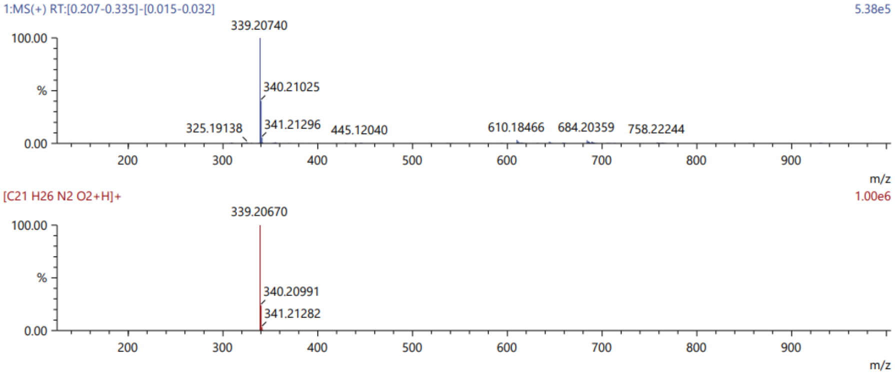

X ray crystallographic data of key intermediates.

3.1. Single-crystal X-ray Diffraction Analysis of pentacyclic ketone 11.

Table A1. Crystal data and structure refinement for **11.**

Empirical formula	$C_{19}H_{20}N_2O$	
Formula weight	292.37	
Temperature	293(2) K	
Wavelength	1.54178 Å	
Crystal system	Monoclinic	
Space group	$P2_1$	
Unit cell dimensions	a = 10.3448(2) Å	a= 90°.
	b = 7.8977(2) Å	b= 117.6662(4)°.
	c = 10.7674(2) Å	g = 90°.
Volume	779.12(3) $Å^3$	
Z	2	
Density (calculated)	1.246 Mg/m^3	
Absorption coefficient	0.609 mm^{-1}	
F(000)	312	
Crystal size	0.330 x 0.270 x 0.088 mm^3	
Theta range for data collection	4.637 to 74.631°.	
Index ranges	-12<=h<=12, -9<=k<=9, -13<=l<=13	
Reflections collected	17046	
Independent reflections	3160 [R_{int} = 0.0234]	
Completeness to theta = 67.679°	99.1 %	
Refinement method	Full-matrix least-squares on F^2	
Data / restraints / parameters	3160 / 1 / 201	
Goodness-of-fit on F^2	1.022	
Final R indices [I>2sigma(I)]	R_1 = 0.0300, wR_2 = 0.0830	
R indices (all data)	R_1 = 0.0301, wR_2 = 0.0831	
Absolute structure parameter	0.07(4)	
Largest diff. peak and hole	0.156 and -0.117 e.$Å^{-3}$	

Table A2. Atomic coordinates (x 10^4) and equivalent isotropic displacement parameters ($Å^2$x 10^3) for **11**. U(eq) is defined as one third of the trace of the orthogonalized U^{ij} tensor.

	x	y	z	U(eq)

N(1)	8010(2)	4675(2)	6899(2)	46(1)
C(2)	6675(2)	4951(2)	6856(2)	38(1)
C(3)	5442(2)	3716(2)	6239(2)	36(1)
N(4)	4096(1)	4609(2)	6031(1)	36(1)
C(5)	4368(2)	5411(2)	7377(2)	39(1)
C(6)	5403(2)	6925(2)	7721(2)	45(1)
C(7)	6699(2)	6404(2)	7547(2)	41(1)
C(8)	8136(2)	7099(2)	8059(2)	47(1)
C(9)	8856(3)	8533(3)	8854(2)	63(1)
C(10)	10314(3)	8773(3)	9193(2)	79(1)
C(11)	11068(3)	7634(4)	8782(2)	76(1)
C(12)	10398(2)	6224(4)	8008(2)	63(1)
C(13)	8926(2)	5984(2)	7644(2)	48(1)
C(14)	5754(2)	2151(2)	7222(2)	42(1)
C(15)	4780(2)	2294(2)	7951(2)	42(1)
C(16)	4941(2)	4066(2)	8539(2)	41(1)
O(17)	5422(2)	4384(2)	9769(1)	62(1)
C(18)	1411(2)	4133(3)	4927(2)	55(1)
C(19)	2915(2)	3322(2)	5599(2)	41(1)
C(20)	3203(2)	2173(2)	6833(2)	45(1)
C(21)	2243(3)	1208(4)	6970(3)	77(1)
C(22)	8422(3)	3212(3)	6351(3)	70(1)

Table A3. Bond lengths [Å] and angles [°] for **11.**

N(1)-C(2)	1.378(2)	N(1)-C(13)	1.380(2)
N(1)-C(22)	1.449(3)	C(2)-C(7)	1.361(2)
C(2)-C(3)	1.494(2)	C(3)-N(4)	1.482(2)
C(3)-C(14)	1.561(2)	C(3)-H(3)	0.9800
N(4)-C(5)	1.4842(19)	N(4)-C(19)	1.488(2)
C(5)-C(6)	1.531(2)	C(5)-C(16)	1.535(2)
C(5)-H(5)	0.9800	C(6)-C(7)	1.496(2)
C(6)-H(6A)	0.9700	C(6)-H(6B)	0.9700
C(7)-C(8)	1.433(2)	C(8)-C(9)	1.406(3)
C(8)-C(13)	1.407(3)	C(9)-C(10)	1.389(4)
C(9)-H(9)	0.9300	C(10)-C(11)	1.391(4)
C(10)-H(10)	0.9300	C(11)-C(12)	1.371(4)
C(11)-H(11)	0.9300	C(12)-C(13)	1.398(3)
C(12)-H(12)	0.9300	C(14)-C(15)	1.541(2)
C(14)-H(14A)	0.9700	C(14)-H(14B)	0.9700
C(15)-C(16)	1.513(2)	C(15)-C(20)	1.515(2)
C(15)-H(15)	0.9800	C(16)-O(17)	1.205(2)
C(18)-C(19)	1.519(2)	C(18)-H(18A)	0.9600
C(18)-H(18B)	0.9600	C(18)-H(18C)	0.9600
C(19)-C(20)	1.520(2)	C(19)-H(19)	0.9800
C(20)-C(21)	1.314(3)	C(21)-H(21A)	0.9300
C(21)-H(21B)	0.9300	C(22)-H(22A)	0.9600
C(22)-H(22B)	0.9600	C(22)-H(22C)	0.9600
C(2)-N(1)-C(13)	107.93(15)	C(2)-N(1)-C(22)	126.33(15)
C(13)-N(1)-C(22)	125.61(16)	C(7)-C(2)-N(1)	110.57(14)
C(7)-C(2)-C(3)	126.05(14)	N(1)-C(2)-C(3)	123.16(14)
N(4)-C(3)-C(2)	107.74(12)	N(4)-C(3)-C(14)	111.29(12)
C(2)-C(3)-C(14)	110.50(13)	N(4)-C(3)-H(3)	109.1
C(2)-C(3)-H(3)	109.1	C(14)-C(3)-H(3)	109.1
C(3)-N(4)-C(5)	108.47(11)	C(3)-N(4)-C(19)	107.44(12)
C(5)-N(4)-C(19)	110.63(12)	N(4)-C(5)-C(6)	111.31(13)
N(4)-C(5)-C(16)	109.10(13)	C(6)-C(5)-C(16)	112.43(13)
N(4)-C(5)-H(5)	107.9	C(6)-C(5)-H(5)	107.9
C(16)-C(5)-H(5)	107.9	C(7)-C(6)-C(5)	108.77(13)
C(7)-C(6)-H(6A)	109.9	C(5)-C(6)-H(6A)	109.9
C(7)-C(6)-H(6B)	109.9	C(5)-C(6)-H(6B)	109.9
H(6A)-C(6)-H(6B)	108.3	C(2)-C(7)-C(8)	106.59(15)
C(2)-C(7)-C(6)	120.68(15)	C(8)-C(7)-C(6)	132.61(16)
C(9)-C(8)-C(13)	118.40(19)	C(9)-C(8)-C(7)	134.8(2)
C(13)-C(8)-C(7)	106.83(15)	C(10)-C(9)-C(8)	118.2(2)
C(10)-C(9)-H(9)	120.9	C(8)-C(9)-H(9)	120.9
C(9)-C(10)-C(11)	121.9(2)	C(9)-C(10)-H(10)	119.0
C(11)-C(10)-H(10)	119.0	C(12)-C(11)-C(10)	121.2(2)

Table A4. (continued).

C(12)-C(11)-H(11)	119.4	C(10)-C(11)-H(11)	119.4
C(11)-C(12)-C(13)	117.3(3)	C(11)-C(12)-H(12)	121.4
C(13)-C(12)-H(12)	121.4	N(1)-C(13)-C(12)	129.0(2)
N(1)-C(13)-C(8)	108.08(15)	C(12)-C(13)-C(8)	122.92(19)
C(15)-C(14)-C(3)	108.00(12)	C(15)-C(14)-H(14A)	110.1
C(3)-C(14)-H(14A)	110.1	C(15)-C(14)-H(14B)	110.1
C(3)-C(14)-H(14B)	110.1	H(14A)-C(14)-H(14B)	108.4

C(16)-C(15)-C(20)	105.03(14)	C(16)-C(15)-C(14)	108.07(13)
C(20)-C(15)-C(14)	107.95(14)	C(16)-C(15)-H(15)	111.8
C(20)-C(15)-H(15)	111.8	C(14)-C(15)-H(15)	111.8
O(17)-C(16)-C(15)	124.21(16)	O(17)-C(16)-C(5)	123.86(16)
C(15)-C(16)-C(5)	111.90(13)	C(19)-C(18)-H(18A)	109.5
C(19)-C(18)-H(18B)	109.5	H(18A)-C(18)-H(18B)	109.5
C(19)-C(18)-H(18C)	109.5	H(18A)-C(18)-H(18C)	109.5
H(18B)-C(18)-H(18C)	109.5	N(4)-C(19)-C(18)	111.82(15)
N(4)-C(19)-C(20)	109.59(12)	C(18)-C(19)-C(20)	114.20(15)
N(4)-C(19)-H(19)	106.9	C(18)-C(19)-H(19)	106.9
C(20)-C(19)-H(19)	106.9	C(21)-C(20)-C(15)	122.61(18
C(21)-C(20)-C(19)	126.37(18)	C(15)-C(20)-C(19)	111.01(13)
C(20)-C(21)-H(21A)	120.0	C(20)-C(21)-H(21B)	120.0
H(21A)-C(21)-H(21B)	120.0	N(1)-C(22)-H(22A)	109.5
N(1)-C(22)-H(22B)	109.5	H(22A)-C(22)-H(22B)	109.5
N(1)-C(22)-H(22C)	109.5	H(22A)-C(22)-H(22C)	109.5
H(22B)-C(22)-H(22C)	109.5		

Table A5. Anisotropic displacement parameters ($Å^2$x 10^3) for **11**. The anisotropic displacement factor exponent takes the form: $-2p^2[h^2a^{*2}U^{11} + ... + 2 h k a^* b^* U^{12}]$.

	U^{11}	U^{22}	U^{33}	U^{23}	U^{13}	U^{12}
N(1)	43(1)	48(1)	54(1)	-9(1)	29(1)	-9(1)
C(2)	43(1)	36(1)	41(1)	-4(1)	23(1)	-6(1)
C(3)	40(1)	36(1)	36(1)	-7(1)	21(1)	-5(1)
N(4)	38(1)	38(1)	32(1)	0(1)	16(1)	0(1)
C(5)	42(1)	40(1)	37(1)	-2(1)	21(1)	4(1)
C(6)	57(1)	36(1)	44(1)	-6(1)	26(1)	1(1)
C(7)	50(1)	35(1)	38(1)	-4(1)	21(1)	-7(1)
C(8)	57(1)	44(1)	36(1)	0(1)	18(1)	-15(1)
C(9)	83(1)	52(1)	45(1)	-9(1)	22(1)	-26(1)
C(10)	85(2)	80(2)	49(1)	-9(1)	12(1)	-48(1)
C(11)	60(1)	103(2)	51(1)	3(1)	14(1)	-39(1)
C(12)	49(1)	85(2)	53(1)	6(1)	21(1)	-17(1)
C(13)	47(1)	54(1)	41(1)	1(1)	19(1)	-13(1)
C(14)	41(1)	33(1)	52(1)	-1(1)	21(1)	0(1)
C(15)	42(1)	41(1)	39(1)	8(1)	16(1)	-1(1)
C(16)	39(1)	51(1)	34(1)	1(1)	18(1)	-1(1)
O(17)	78(1)	70(1)	35(1)	-3(1)	24(1)	2(1)
C(18)	39(1)	67(1)	49(1)	6(1)	12(1)	3(1)
C(19)	38(1)	45(1)	36(1)	0(1)	14(1)	-4(1)
C(20)	41(1)	47(1)	46(1)	4(1)	19(1)	-4(1)
C(21)	54(1)	87(2)	78(1)	27(1)	22(1)	-20(1)
C(22)	60(1)	66(1)	101(2)	-24(1)	52(1)	-7(1)

Table A6. Hydrogen coordinates (x 10^4) and isotropic displacement parameters ($Å^2$x 10^3) for **11.**

	x	y	z	U(eq)
H(3)	5328	3335	5327	43
H(5)	3433	5827	7280	47
H(6A)	4906	7860	7097	54
H(6B)	5714	7293	8677	54
H(9)	8370	9300	9144	76
H(10)	10799	9722	9710	94
H(11)	12045	7833	9037	91
H(12)	10902	5458	7736	76
H(14A)	5536	1113	6680	51
H(14B)	6775	2131	7917	51
H(15)	5022	1429	8678	50
H(18A)	1275	4754	4109	82
H(18B)	1329	4890	5585	82
H(18C)	679	3267	4664	82
H(19)	2976	2615	4880	49
H(21A)	2522	564	7778	92
H(21B)	1285	1168	6260	92
H(22A)	8768	3577	5707	105
H(22B)	7589	2489	5872	105
H(22C)	9181	2600	7109	105

Table A7. Torsion angles [°] for KPP-II-50 **11**.

C(13)-N(1)-C(2)-C(7)	0.2(2)	C(22)-N(1)-C(2)-C(7)	176.1(2)
C(13)-N(1)-C(2)-C(3)	-174.71(14)	C(22)-N(1)-C(2)-C(3)	1.2(3)
C(7)-C(2)-C(3)-N(4)	20.7(2)	N(1)-C(2)-C(3)-N(4)	-165.14(14)
C(7)-C(2)-C(3)-C(14)	-101.03(18)	N(1)-C(2)-C(3)-C(14)	73.09(18)
C(2)-C(3)-N(4)-C(5)	-52.90(16)	C(14)-C(3)-N(4)-C(5)	68.39(16)
C(2)-C(3)-N(4)-C(19)	-172.52(11)	C(14)-C(3)-N(4)-C(19)	-51.24(15)
C(3)-N(4)-C(5)-C(6)	71.37(16)	C(19)-N(4)-C(5)-C(6)	-171.02(13)
C(3)-N(4)-C(5)-C(16)	-53.27(16)	C(19)-N(4)-C(5)-C(16)	64.34(16)
N(4)-C(5)-C(6)-C(7)	-48.69(18)	C(16)-C(5)-C(6)-C(7)	74.05(17)
N(1)-C(2)-C(7)-C(8)	0.18(19)	C(3)-C(2)-C(7)-C(8)	174.92(15)
N(1)-C(2)-C(7)-C(6)	-176.31(15)	C(3)-C(2)-C(7)-C(6)	-1.6(2)
C(5)-C(6)-C(7)-C(2)	14.6(2)	C(5)-C(6)-C(7)-C(8)	-160.79(17)
C(2)-C(7)-C(8)-C(9)	-178.8(2)	C(6)-C(7)-C(8)-C(9)	-2.9(3)
C(2)-C(7)-C(8)-C(13)	-0.49(18)	C(6)-C(7)-C(8)-C(13)	175.40(18)
C(13)-C(8)-C(9)-C(10)	-0.2(3)	C(7)-C(8)-C(9)-C(10)	178.0(2)
C(8)-C(9)-C(10)-C(11)	-0.6(3)	C(9)-C(10)-C(11)-C(12)	0.6(4)
C(10)-C(11)-C(12)-C(13)	0.3(3)	C(2)-N(1)-C(13)-C(12)	177.44(18)
C(22)-N(1)-C(13)-C(12)	1.5(3)	C(2)-N(1)-C(13)-C(8)	-0.5(2)
C(22)-N(1)-C(13)-C(8)	-176.5(2)	C(11)-C(12)-C(13)-N(1)	-178.88(19)
C(11)-C(12)-C(13)-C(8)	-1.2(3)	C(9)-C(8)-C(13)-N(1)	179.26(16)
C(7)-C(8)-C(13)-N(1)	0.63(19)	C(9)-C(8)-C(13)-C(12)	1.1(3)
C(7)-C(8)-C(13)-C(12)	-177.49(17)	N(4)-C(3)-C(14)-C(15)	-13.88(17)
C(2)-C(3)-C(14)-C(15)	105.77(15)	C(3)-C(14)-C(15)-C(16)	-48.74(17)
C(3)-C(14)-C(15)-C(20)	64.37(16)	C(20)-C(15)-C(16)-O(17)	127.24(18)
C(14)-C(15)-C(16)-O(17)	-117.71(18)	C(20)-C(15)-C(16)-C(5)	-50.81(16)
C(14)-C(15)-C(16)-C(5)	64.23(17)	N(4)-C(5)-C(16)-O(17)	170.62(16)
C(6)-C(5)-C(16)-O(17)	46.6(2)	N(4)-C(5)-C(16)-C(15)	-11.32(17)
C(6)-C(5)-C(16)-C(15)	-135.30(14)	C(3)-N(4)-C(19)-C(18)	-162.24(14)
C(5)-N(4)-C(19)-C(18)	79.53(17)	C(3)-N(4)-C(19)-C(20)	70.08(16)
C(5)-N(4)-C(19)-C(20)	-48.15(18)	C(16)-C(15)-C(20)-C(21)	-111.0(2)
C(14)-C(15)-C(20)-C(21)	133.9(2)	C(16)-C(15)-C(20)-C(19)	67.80(17)
C(14)-C(15)-C(20)-C(19)	-47.33(19)	N(4)-C(19)-C(20)-C(21)	160.8(2)
C(18)-C(19)-C(20)-C(21)	34.4(3)	N(4)-C(19)-C(20)-C(15)	-18.0(2)
C(18)-C(19)-C(20)-C(15)	-144.29(16)		

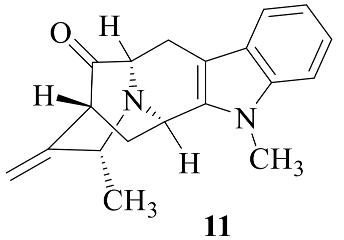

11

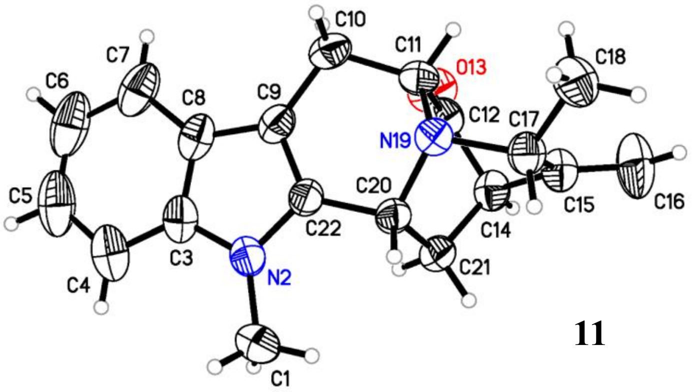

11

3.2. X-ray Crystal Data for Compound 19.

Table A8. Crystal data and structure refinement for **19**.

Empirical formula	$C_{19}H_{20}N_2O$	
Formula weight	292.37	
Temperature	296(2) K	
Wavelength	0.71073 Å	
Crystal system	Monoclinic	
Space group	$P2_1$	
Unit cell dimensions	a = 8.7184(3) Å	a= 90°.
	b = 8.4717(3) Å	b= 92.9370(10)°.
	c = 10.3859(3) Å	g = 90°.
Volume	766.09(4) $Å^3$	
Z	2	
Density (20°C)	1.267 Mg/m^3	
Absorption coefficient	0.079 mm^{-1}	
F(000)	312	
Crystal size	0.452 x 0.355 x 0.272 mm^3	
Theta range for data collection	1.963 to 29.151°.	
Index ranges	-10<=h<=11, -11<=k<=11, -14<=l<=14	
Reflections collected	8923	
Independent reflections	4066 [R_{int} = 0.0142]	
Completeness to theta = 25.242°	99.2 %	
Refinement method	Full-matrix least-squares on F^2	
Data / restraints / parameters	4066 / 1 / 201	
Goodness-of-fit on F^2	0.916	
Final R indices [I>2sigma(I)]	R_1 = 0.0340, wR_2 = 0.0954	
R indices (all data)	R_1 = 0.0368, wR_2 = 0.0992	
Absolute structure parameter	0.4(3)	
Largest diff. peak and hole	0.212 and -0.162 e.$Å^{-3}$	

Table A9. Atomic coordinates (x 10^4) and equivalent isotropic displacement parameters ($Å^2$x 10^3) for **19**. U(eq) is defined as one third of the trace of the orthogonalized U^{ij} tensor.

	x	y	z	U(eq)
C(1)	634(2)	6863(2)	11114(2)	45(1)
C(2)	-20(2)	6638(2)	10100(2)	36(1)
C(3)	-826(2)	6373(2)	8827(2)	34(1)
C(3A)	-2332(2)	7291(2)	8738(2)	43(1)
N(4)	98(1)	6754(1)	7712(1)	32(1)
C(5)	1029(2)	8200(2)	7870(2)	34(1)
C(6)	2561(2)	7972(2)	8644(2)	39(1)
O(6)	3189(2)	9094(2)	9167(2)	61(1)
C(7)	3334(2)	6369(2)	8639(2)	46(1)
C(8)	2288(2)	4963(2)	8314(2)	38(1)
C(9)	1044(2)	5427(2)	7284(1)	33(1)
C(10)	1755(2)	5974(2)	6074(2)	33(1)
N(11)	2494(2)	4992(2)	5236(1)	36(1)
C(11)	2513(2)	3280(2)	5231(2)	44(1)
C(12)	3106(2)	5934(2)	4301(1)	36(1)
C(13)	3982(2)	5519(2)	3263(2)	43(1)
C(14)	4420(2)	6713(3)	2454(2)	48(1)
C(15)	4007(2)	8286(3)	2664(2)	48(1)
C(16)	3180(2)	8712(2)	3716(2)	42(1)
C(17)	2728(2)	7526(2)	4559(2)	35(1)
C(18)	1890(2)	7520(2)	5714(2)	34(1)
C(19)	1345(2)	8851(2)	6523(2)	38(1)

Table A10. Bond lengths [Å] and angles [°] for **19**.

C(1)-C(2)	1.187(3)	C(1)-H(1)	0.9300
C(2)-C(3)	1.482(2)	C(3)-N(4)	1.4804(19)
C(3)-C(3A)	1.524(2)	C(3)-H(3A)	0.9800
C(3A)-H(3D)	0.9600	C(3A)-H(3E)	0.9600
C(3A)-H(3F)	0.9600	N(4)-C(5)	1.4739(18)
N(4)-C(9)	1.4767(19)	C(5)-C(6)	1.535(2)
C(5)-C(19)	1.543(2)	C(5)-H(5A)	0.9800
C(6)-O(6)	1.211(2)	C(6)-C(7)	1.516(3)
C(7)-C(8)	1.527(3)	C(7)-H(7A)	0.9700
C(7)-H(7B)	0.9700	C(8)-C(9)	1.535(2)
C(8)-H(8A)	0.9700	C(8)-H(8B)	0.9700
C(9)-C(10)	1.503(2)	C(9)-H(9A)	0.9800
C(10)-C(18)	1.369(2)	C(10)-N(11)	1.386(2)
N(11)-C(12)	1.385(2)	N(11)-C(11)	1.451(2)
C(11)-H(11A)	0.9600	C(11)-H(11B)	0.9600
C(11)-H(11C)	0.9600	C(12)-C(13)	1.398(2)
C(12)-C(17)	1.417(2)	C(13)-C(14)	1.381(3)
C(13)-H(13A)	0.9300	C(14)-C(15)	1.400(3)
C(14)-H(14A)	0.9300	C(15)-C(16)	1.387(3)
C(15)-H(15A)	0.9300	C(16)-C(17)	1.403(2)
C(16)-H(16A)	0.9300	C(17)-C(18)	1.435(2)
C(18)-C(19)	1.498(2)	C(19)-H(19C)	0.9700
C(19)-H(19A)	0.9700		
C(2)-C(1)-H(1)	180.0	C(1)-C(2)-C(3)	179.33(19)
N(4)-C(3)-C(2)	114.36(12)	N(4)-C(3)-C(3A)	110.00(12)
C(2)-C(3)-C(3A)	110.18(13)	N(4)-C(3)-H(3A)	107.3
C(2)-C(3)-H(3A)	107.3	C(3A)-C(3)-H(3A)	107.3
C(3)-C(3A)-H(3D)	109.5	C(3)-C(3A)-H(3E)	109.5
H(3D)-C(3A)-H(3E)	109.5	C(3)-C(3A)-H(3F)	109.5
H(3D)-C(3A)-H(3F)	109.5	H(3E)-C(3A)-H(3F)	109.5
C(5)-N(4)-C(9)	110.68(11)	C(5)-N(4)-C(3)	114.38(12)
C(9)-N(4)-C(3)	113.83(11)	N(4)-C(5)-C(6)	114.46(12)
N(4)-C(5)-C(19)	108.61(12)	C(6)-C(5)-C(19)	109.20(13)
N(4)-C(5)-H(5A)	108.1	C(6)-C(5)-H(5A)	108.1
C(19)-C(5)-H(5A)	108.1	O(6)-C(6)-C(7)	120.92(17)
O(6)-C(6)-C(5)	119.84(18)	C(7)-C(6)-C(5)	119.01(13)
C(6)-C(7)-C(8)	116.06(14)	C(6)-C(7)-H(7A)	108.3
C(8)-C(7)-H(7A)	108.3	C(6)-C(7)-H(7B)	108.3
C(8)-C(7)-H(7B)	108.3	H(7A)-C(7)-H(7B)	107.4
C(7)-C(8)-C(9)	110.29(13)	C(7)-C(8)-H(8A)	109.6
C(9)-C(8)-H(8A)	109.6	C(7)-C(8)-H(8B)	109.6
C(9)-C(8)-H(8B)	109.6	H(8A)-C(8)-H(8B)	108.1
N(4)-C(9)-C(10)	106.09(12)	N(4)-C(9)-C(8)	111.79(12)

Table A11. (continued).

C(10)-C(9)-C(8)	110.78(13)	N(4)-C(9)-H(9A)	109.4
C(10)-C(9)-H(9A)	109.4	C(8)-C(9)-H(9A)	109.4
C(18)-C(10)-N(11)	110.66(14)	C(18)-C(10)-C(9)	124.69(13)
N(11)-C(10)-C(9)	124.39(13)	C(12)-N(11)-C(10)	107.63(13)
C(12)-N(11)-C(11)	124.67(15)	C(10)-N(11)-C(11)	127.44(15)
N(11)-C(11)-H(11A)	109.5	N(11)-C(11)-H(11B)	109.5
H(11A)-C(11)-H(11B)	109.5	N(11)-C(11)-H(11C)	109.5

H(11A)-C(11)-H(11C)	109.5	H(11B)-C(11)-H(11C)	109.5
N(11)-C(12)-C(13)	129.90(16)	N(11)-C(12)-C(17)	108.30(14)
C(13)-C(12)-C(17)	121.79(15)	C(14)-C(13)-C(12)	117.65(18)
C(14)-C(13)-H(13A)	121.2	C(12)-C(13)-H(13A)	121.2
C(13)-C(14)-C(15)	121.40(17)	C(13)-C(14)-H(14A)	119.3
C(15)-C(14)-H(14A)	119.3	C(16)-C(15)-C(14)	121.21(17)
C(16)-C(15)-H(15A)	119.4	C(14)-C(15)-H(15A)	119.4
C(15)-C(16)-C(17)	118.65(17)	C(15)-C(16)-H(16A)	120.7
C(17)-C(16)-H(16A)	120.7	C(16)-C(17)-C(12)	119.21(15)
C(16)-C(17)-C(18)	134.04(15)	C(12)-C(17)-C(18)	106.75(13)
C(10)-C(18)-C(17)	106.63(13)	C(10)-C(18)-C(19)	122.17(14)
C(17)-C(18)-C(19)	130.96(14)	C(18)-C(19)-C(5)	108.42(12)
C(18)-C(19)-H(19C)	110.0	C(5)-C(19)-H(19C)	110.0
C(18)-C(19)-H(19A)	110.0	C(5)-C(19)-H(19A)	110.0
H(19C)-C(19)-H(19A)	108.		

Table A12. Anisotropic displacement parameters ($Å^2 x 10^3$) for **19**. The anisotropic displacement factor exponent takes the form: $-2p^2[h^2a^{*2}U^{11} + ... + 2 h k a^* b^* U^{12}]$.

	U^{11}	U^{22}	U^{33}	U^{23}	U^{13}	U^{12}
C(1)	45(1)	42(1)	46(1)	1(1)	-5(1)	-2(1)
C(2)	32(1)	34(1)	42(1)	5(1)	4(1)	0(1)
C(3)	30(1)	32(1)	40(1)	2(1)	2(1)	-2(1)
C(3A)	31(1)	51(1)	47(1)	-1(1)	1(1)	4(1)
N(4)	31(1)	25(1)	39(1)	0(1)	3(1)	-1(1)
C(5)	36(1)	26(1)	40(1)	-3(1)	5(1)	-2(1)
C(6)	36(1)	41(1)	41(1)	-3(1)	3(1)	-9(1)
O(6)	59(1)	54(1)	70(1)	-12(1)	-7(1)	-20(1)
C(7)	34(1)	52(1)	51(1)	-3(1)	-6(1)	2(1)
C(8)	39(1)	35(1)	41(1)	4(1)	3(1)	8(1)
C(9)	36(1)	24(1)	38(1)	0(1)	2(1)	-1(1)
C(10)	37(1)	27(1)	35(1)	-2(1)	1(1)	1(1)
N(11)	44(1)	27(1)	37(1)	-3(1)	3(1)	2(1)
C(11)	56(1)	27(1)	49(1)	-6(1)	-3(1)	5(1)
C(12)	36(1)	36(1)	34(1)	-3(1)	-1(1)	-2(1)
C(13)	39(1)	50(1)	40(1)	-11(1)	0(1)	0(1)
C(14)	38(1)	67(1)	37(1)	-5(1)	3(1)	-7(1)
C(15)	41(1)	60(1)	41(1)	7(1)	3(1)	-12(1)
C(16)	43(1)	40(1)	43(1)	5(1)	1(1)	-7(1)
C(17)	35(1)	35(1)	35(1)	-1(1)	-1(1)	-2(1)
C(18)	39(1)	27(1)	37(1)	1(1)	4(1)	0(1)
C(19)	45(1)	24(1)	45(1)	1(1)	7(1)	1(1)

Table A13. Hydrogen coordinates (x 10^4) and isotropic displacement parameters (Å^{2}x 10^3) for **19**.

	x	y	z	U(eq)
H(1)	1146	7040	11909	53
H(3A)	-1085	5248	8771	41
H(3D)	-2895	7025	7949	64
H(3E)	-2117	8402	8751	64
H(3F)	-2931	7024	9456	64
H(5A)	426	8986	8317	40
H(7A)	3832	6192	9483	55
H(7B)	4131	6398	8021	55
H(8A)	1808	4610	9085	46
H(8B)	2893	4097	7998	46
H(9A)	382	4514	7086	39
H(11A)	3555	2915	5329	66
H(11B)	2055	2901	4430	66
H(11C)	1942	2892	5931	66
H(13A)	4260	4476	3122	52
H(14A)	5003	6467	1756	57
H(15A)	4291	9059	2087	57
H(16A)	2931	9763	3859	51
H(19C)	414	9304	6126	45
H(19A)	2122	9671	6595	45

Table A14. Torsion angles [°] for **19**.

C(2)-C(3)-N(4)-C(5)	-40.66(18)	C(3A)-C(3)-N(4)-C(5)	83.94(16)
C(2)-C(3)-N(4)-C(9)	87.94(16)	C(3A)-C(3)-N(4)-C(9)	-147.46(14)
C(9)-N(4)-C(5)-C(6)	-48.11(17)	C(3)-N(4)-C(5)-C(6)	82.07(16)
C(9)-N(4)-C(5)-C(19)	74.20(15)	C(3)-N(4)-C(5)-C(19)	-155.63(12)
N(4)-C(5)-C(6)-O(6)	-157.51(16)	C(19)-C(5)-C(6)-O(6)	80.51(19)
N(4)-C(5)-C(6)-C(7)	28.0(2)	C(19)-C(5)-C(6)-C(7)	-94.00(17)
O(6)-C(6)-C(7)-C(8)	162.78(17)	C(5)-C(6)-C(7)-C(8)	-22.8(2)
C(6)-C(7)-C(8)-C(9)	36.5(2)	C(5)-N(4)-C(9)-C(10)	-55.49(16)
C(3)-N(4)-C(9)-C(10)	174.05(12)	C(5)-N(4)-C(9)-C(8)	65.37(16)
C(3)-N(4)-C(9)-C(8)	-65.09(16)	C(7)-C(8)-C(9)-N(4)	-58.67(17)
C(7)-C(8)-C(9)-C(10)	59.43(17)	N(4)-C(9)-C(10)-C(18)	19.1(2)
C(8)-C(9)-C(10)-C(18)	-102.44(19)	N(4)-C(9)-C(10)-N(11)	-167.46(14)
C(8)-C(9)-C(10)-N(11)	71.02(19)	C(18)-C(10)-N(11)-C(12)	-0.70(19)
C(9)-C(10)-N(11)-C(12)	-174.96(14)	C(18)-C(10)-N(11)-C(11)	-174.96(16)
C(9)-C(10)-N(11)-C(11)	10.8(3)	C(10)-N(11)-C(12)-C(13)	178.33(16)
C(11)-N(11)-C(12)-C(13)	-7.2(3)	C(10)-N(11)-C(12)-C(17)	-0.54(18)
C(11)-N(11)-C(12)-C(17)	173.91(16)	N(11)-C(12)-C(13)-C(14)	178.63(17)
C(17)-C(12)-C(13)-C(14)	-2.6(2)	C(12)-C(13)-C(14)-C(15)	0.1(3)
C(13)-C(14)-C(15)-C(16)	2.0(3)	C(14)-C(15)-C(16)-C(17)	-1.6(3)
C(15)-C(16)-C(17)-C(12)	-0.9(2)	C(15)-C(16)-C(17)-C(18)	179.82(18)
N(11)-C(12)-C(17)-C(16)	-177.92(14)	C(13)-C(12)-C(17)-C(16)	3.1(2)
N(11)-C(12)-C(17)-C(18)	1.50(18)	C(13)-C(12)-C(17)-C(18)	-177.47(15)
N(11)-C(10)-C(18)-C(17)	1.63(19)	C(9)-C(10)-C(18)-C(17)	175.86(14)
N(11)-C(10)-C(18)-C(19)	-173.45(15)	C(9)-C(10)-C(18)-C(19)	0.8(3)
C(16)-C(17)-C(18)-C(10)	177.41(18)	C(12)-C(17)-C(18)-C(10)	-1.90(18)
C(16)-C(17)-C(18)-C(19)	-8.1(3)	C(12)-C(17)-C(18)-C(19)	172.58(17)
C(10)-C(18)-C(19)-C(5)	13.5(2)	C(17)-C(18)-C(19)-C(5)	-160.21(16)
N(4)-C(5)-C(19)-C(18)	-48.15(17)	C(6)-C(5)-C(19)-C(18)	77.29(16)

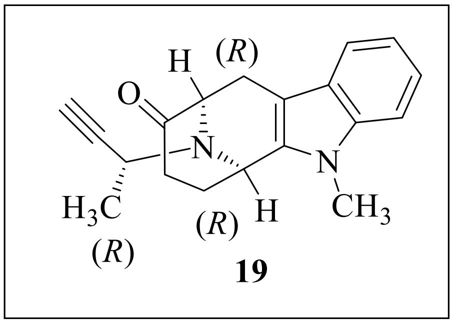

19

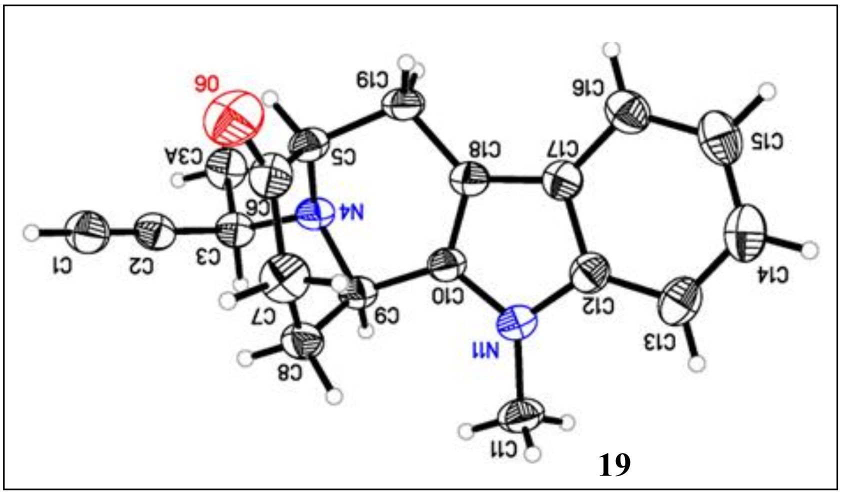

19

References

1. Rahman, M. T.; Deschamps, J. R.; Imler, G. H.; Cook, J. M., Total Synthesis of Sarpagine-Related Bioactive Indole Alkaloids. Chemistry **2018,** *24* (10), 2354-2359.

2. Rahman, M. T., Shorter and Improved Access to the Key Tetracyclic Core of Sarpagine-Macroline-Ajmaline Indole Alkaloids: The Total Synthesis of Alkaloids Macrocarpines Ag, Talcarpine, N (4)-methyl-n (4), 21-secotalpinine, Deoxyperaksine, Dihydroperaksine, Talpinine, O-acetyltalpinine, and N (4)-methyltalpinine. Ph.D. Dissertation. University of Wisconsin Milwaukee. Milwaukee, WI, 2018.

3. Edwankar, C. R., Part I: The First Regio-and Atropdiastereoselective Total Synthesis of The Dimeric Indole Alkaloid (+)-Dispegatrine, as well as The First Total Synthesis of The Sarpagine Alkaloids (+)-Spegatrine, Lochvinerine,(+)-Lochneram and an Improved Total Synthesis of (+)-10-Methoxyvellosimine,(+)-Lochnerine and (+)-Sarpagine. Part II: Studies Directed Toward The Total Synthesis of The Carbon-19 Methyl Substituted Sarpagine-Macroline Alkaloids (+)-Macro-salhine Chloride as well as Macrocarpine A, B and C. Ph.D. Dissertation. The University of Wisconsin-Milwaukee, Milwaukee, WI, 2011.

4. Edwankar, R. V.; Edwankar, C. R.; Deschamps, J. R.; Cook, J. M., General Strategy for Synthesis of C-19 Methyl-Substituted Sarpagine/Macroline/Ajmaline Indole Alkaloids Including Total Synthesis of 19 (S), 20 (R)-Dihydroperaksine, 19 (S), 20 (R)-Dihydroperaksine-17-al, and Peraksine. *J. Org. Chem.* **2014,** *79* (21), 10030-10048.

5. Wearing, X. Z. Enantiospecific Stereospecific Total Synthesis of the Oxindole Alkaloid (+)-Alstonisine and Stereocontrolled Total Synthesis of (-)-11 -methoxy- 17-epivincamajine as well as Studies Directed Toward the Total Synthesis of N_{b}-demethylalstophylline oxindole. Ph.D. Dissertation, Univeristy of Wisconsin-Milwaukee, Milwaukee, WI, 2004.

6. Wong, W.-H.; Lim, P.-B.; Chuah, C.-H., Oxindole Alkaloids From Alstonia macrophylla. *Phytochem.* **1996,** *41*(1), 313-315.

7. Kam t.-S., Choo, Y.-M.; Komiyama, K., Yeun-Mun Choo, Kanki Komiyama, Unusual Spirocyclic Macroline Alkaloids, Nitrogenous Derivatives, and a Cytotoxic Bisindole From Alstonia, Tetrahedron, **2004**, *60* (18), 3957-3966.
