# Supplementary material for: First Total Synthesis of the Unnatural (+)-Talcarpine and (−)‑N 4‑Methyl,N 4‑21-secotalpinine
Source: ACS Omega. 2026 Apr 29;11(18):26942–56. doi: 10.1021/acsomega.5c13509 (PMC13176970; doi:10.1021/acsomega.5c13509)
Supplement: Supplementary file 1 [file ao5c13509_si_001.zip › X-ray data for review/Cook 185 (compound 19)/cook185_experimental.doc]

**Single-crystal X-ray Diffraction Analysis of** KPP-II-43.

C_19_H_20_N_2_O, FW = 292.37, monoclinic, P2_1_, a = 8.7184(3) Å, b = 8.4717(3) Å, c = 10.3859(3) Å, *α* = 90°, *β* = 92.9370(10)°, *γ* = 90°, V = 766.09(4) Å^3^, Z = 2, *ρ*calc (298 K) = 1.267 Mg/m^3^, *μ* = 0.079 mm^-1^, *F*(000) = 312, R_1_ = 0.0340 for XXXX observed (I > 2σI) reflections and 0.0368 for all XXXX reflections, Goodness-of-fit = 0.916, 201 parameters.

A irregular colorless crystal of dimensions 0.452 x 0.355 x 0.272 mm^2^ was mounted on a MiteGen MicroMesh using a small amount of Cargille Immersion Oil. Data were collected on a Bruker three-circle platform diffractometer equipped with a SMART APEX II CCD detector. The crystals were irradiated using graphite monochromated MoK_α_ radiation (λ = 0.71073)].

Data collection was performed and the unit cell was initially refined using *APEX3* [v2015.5-2].^1^ Data Reduction was performed using *SAINT* [v8.34A]^2^ and *XPREP* [v2014/2]^3^. Corrections were applied for Lorentz, polarization, and absorption effects using *SADABS* [v2014/2].^4^ The structure was solved and refined with the aid of the programs SHELXL-2014/7 within WingX.^5,6^ The full-matrix least-squares refinement on F^2^ included atomic coordinates and anisotropic thermal parameters for all non-H atoms. The H atoms were included using a riding model.

1. Bruker (2015). APEX3 v2015.5-2. Bruker AXS Inc., Madison, Wisconsin, USA.
2. Bruker (2013). SAINT v8.34A. Bruker AXS Inc., Madison, Wisconsin, USA.
3. Bruker (2014). XPREP v2014/2. Bruker AXS Inc., Madison, Wisconsin, USA.
4. Bruker (2014). SADABS v2014/5, Bruker AXS Inc., Madison, Wisconsin, USA.
5. Sheldrick, G. M. (2014). SHELXL-2014/7. University of Göttingen, Germany.
6. Farrugia, L. J. (2012). J. Appl. Cryst. 45, 849-854

**Acknowledge**

The authors thank the Office of Naval Research (Award No. N00014-15-WX-0-0149).
