# Supplementary material for: First Total Synthesis of the Unnatural (+)-Talcarpine and (−)‑N 4‑Methyl,N 4‑21-secotalpinine
Source: ACS Omega. 2026 Apr 29;11(18):26942–56. doi: 10.1021/acsomega.5c13509 (PMC13176970; doi:10.1021/acsomega.5c13509)
Supplement: Supplementary file 1 [file ao5c13509_si_001.zip › X-ray data for review/cook 196 (compound 11)/cook196_experimental (1).doc]

**Single-crystal X-ray Diffraction Analysis of KPP-II-50.**

C_19_H_20_N_2_O, FW = 292.37, monoclinic, P2_1_, a = 10.3448(2) Å, b = 7.8977(2) Å, c = 10.7674(2) Å, *α* = 90°, *β* = 117.6662(4)°, *γ* = 90°, V = 779.12(3) Å^3^, Z = 2, *ρ*calc (293K) = 1.246 Mg/m^3^, *μ* = 0.609 mm^-1^, *F*(000) = 312, R_1_ = 0.0300 for 3148 observed (I > 2σI) reflections and 0.0301 for all 3160 reflections, Goodness-of-fit = 1.022, 201 parameters.

A clear colorless plate crystal of dimensions 0.330 x 0.270 x 0.088 mm was mounted on a MiteGen MicroMesh using a small amount of Cargille Immersion Oil. Data were collected on a Bruker three-circle platform diffractometer equipped with a PHOTON II CPAD detector. The crystals were irradiated using a 1μs microfocus CuK_α_ source (λ = 1.54178) with Montel optics. Data was collected at room temperature (20°C).

Data collection was performed and the unit cell was initially refined using *APEX3* [v2015.5-2].^1^ Data Reduction was performed using *SAINT* [v8.34A]^2^ and *XPREP* [v2014/2]^3^. Corrections were applied for Lorentz, polarization, and absorption effects using *SADABS* [v2014/2].^4^ The structure was solved and refined with the aid of the program SHELXL-2014/7.^5^ The full-matrix least-squares refinement on F^2^ included atomic coordinates and anisotropic thermal parameters for all non-H atoms. Hydrogen atoms were located from the difference electron-density maps and added using a riding model.

1. Bruker (2015). APEX3 v2015.5-2. Bruker AXS Inc., Madison, Wisconsin, USA.
2. Bruker (2013). SAINT v8.34A. Bruker AXS Inc., Madison, Wisconsin, USA.
3. Bruker (2014). XPREP v2014/2. Bruker AXS Inc., Madison, Wisconsin, USA.
4. Bruker (2014). SADABS v2014/5, Bruker AXS Inc., Madison, Wisconsin, USA.
5. Sheldrick, G. M. (2014). SHELXL-2014/7. University of Göttingen, Germany.

**Acknowledge**

The authors thank the Office of Naval Research (Award No. N00014-15-WX-0-0149).
