# Supplementary material for: First Total Synthesis of the Unnatural (+)-Talcarpine and (−)‑N 4‑Methyl,N 4‑21-secotalpinine
Source: ACS Omega. 2026 Apr 29;11(18):26942–56. doi: 10.1021/acsomega.5c13509 (PMC13176970; doi:10.1021/acsomega.5c13509)
Supplement: Supplementary file 2 [file ao5c13509_si_002.zip › FID for publications/26/13C NMR (DEPT135)/pdata/1/email_KPP-II-66 (2022)_3_1.pdf]

DEPT 135 Spectrum
c13dept135 CDCl3 /nmr500 kppandey 10

UNIVERSITY of WISCONSIN UWMILWAUKEE

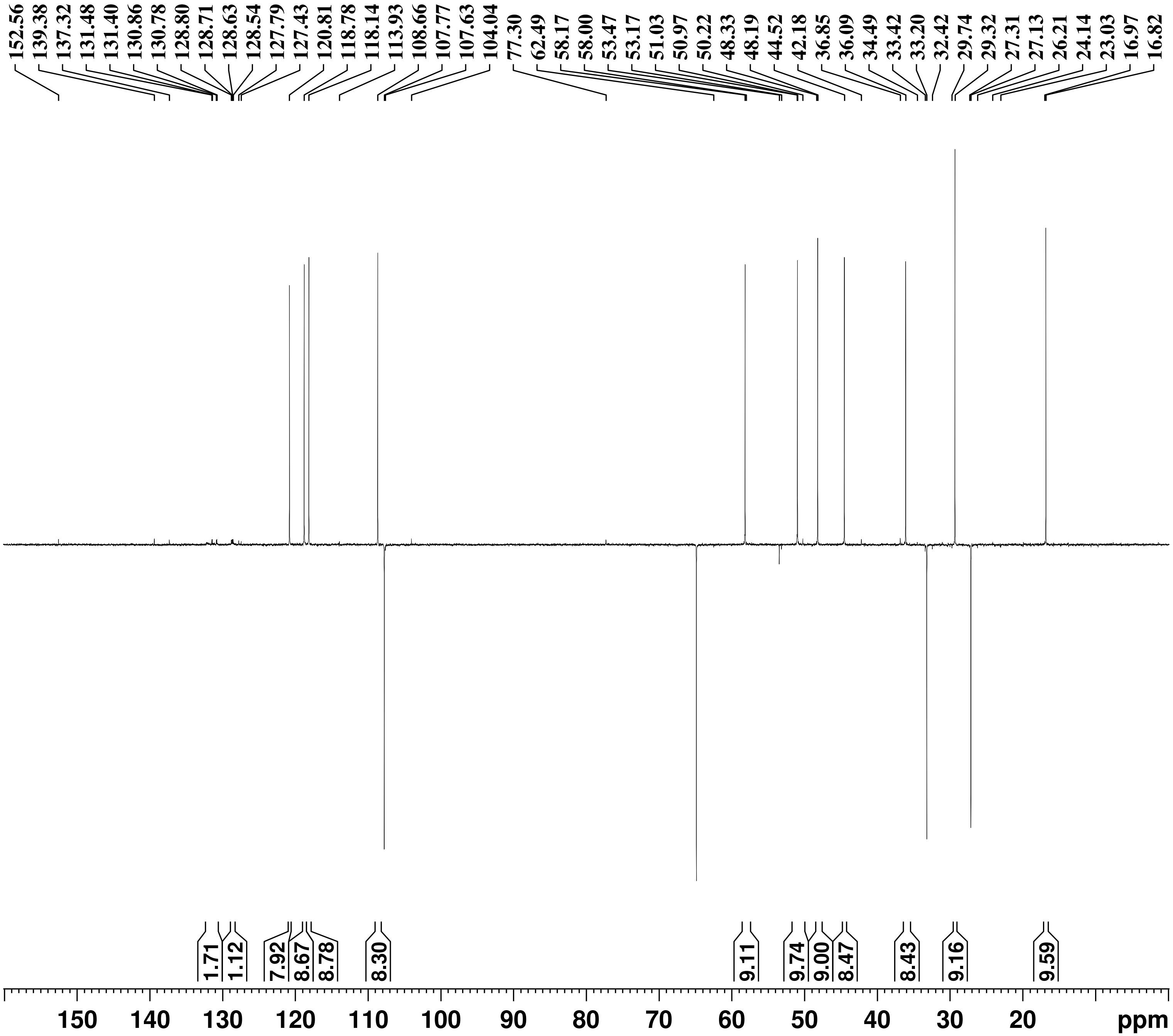


```
Current Data Parameters
NAME       KPP-II-66 (2022)
EXPNO                     3
PROCNO                    1
DATPATH  /nmr500/data/kppandey/nmr

F2 - Acquisition Parameters
Date_              20220218
Time                  17.04 h
INSTRUM               spect
PROBHD   Z149001_0007 (
PULPROG          deptsp135
TD                    65536
SOLVENT               CDCl3
NS                      128
DS                        8
SWH               20161.291 Hz
FIDRES             0.615274 Hz
AQ                1.6252928 sec
RG                   190.86
DW                   24.800 usec
DE                    23.06 usec
TE                    298.0 K
CNST2           145.0000000
D1               2.00000000 sec
D2               0.00344828 sec
D12              0.00002000 sec
TD0                       1
SFO1            125.7678491 MHz
NUC1                    13C
P1                    10.00 usec
P13                 2000.00 usec
PLW0                      0 W
PLW1            64.21800232 W
SPNAM[5]        Crp60comp.4
SPOAL5                0.500
SPOFFS5                   0 Hz
SPW5             9.81169987 W
SFO2            500.1320005 MHz
NUC2                     1H
CPDPRG[2            waltz16
P3                    12.00 usec
P4                    24.00 usec
PCPD2                 80.00 usec
PLW2            14.14599991 W
PLW12            0.31829000 W

F2 - Processing parameters
SI                    32768
SF              125.7577885 MHz
WDW                      EM
SSB                       0
LB                     1.00 Hz
GB                        0
PC                     1.40
```
