# Supplementary material for: First Total Synthesis of the Unnatural (+)-Talcarpine and (−)‑N 4‑Methyl,N 4‑21-secotalpinine
Source: ACS Omega. 2026 Apr 29;11(18):26942–56. doi: 10.1021/acsomega.5c13509 (PMC13176970; doi:10.1021/acsomega.5c13509)
Supplement: Supplementary file 2 [file ao5c13509_si_002.zip › FID for publications/26/13C NMR/pdata/1/email_KPP-II-66 (2022)_2_1.pdf]

Carbon Spectrum ns=256

[illegible]

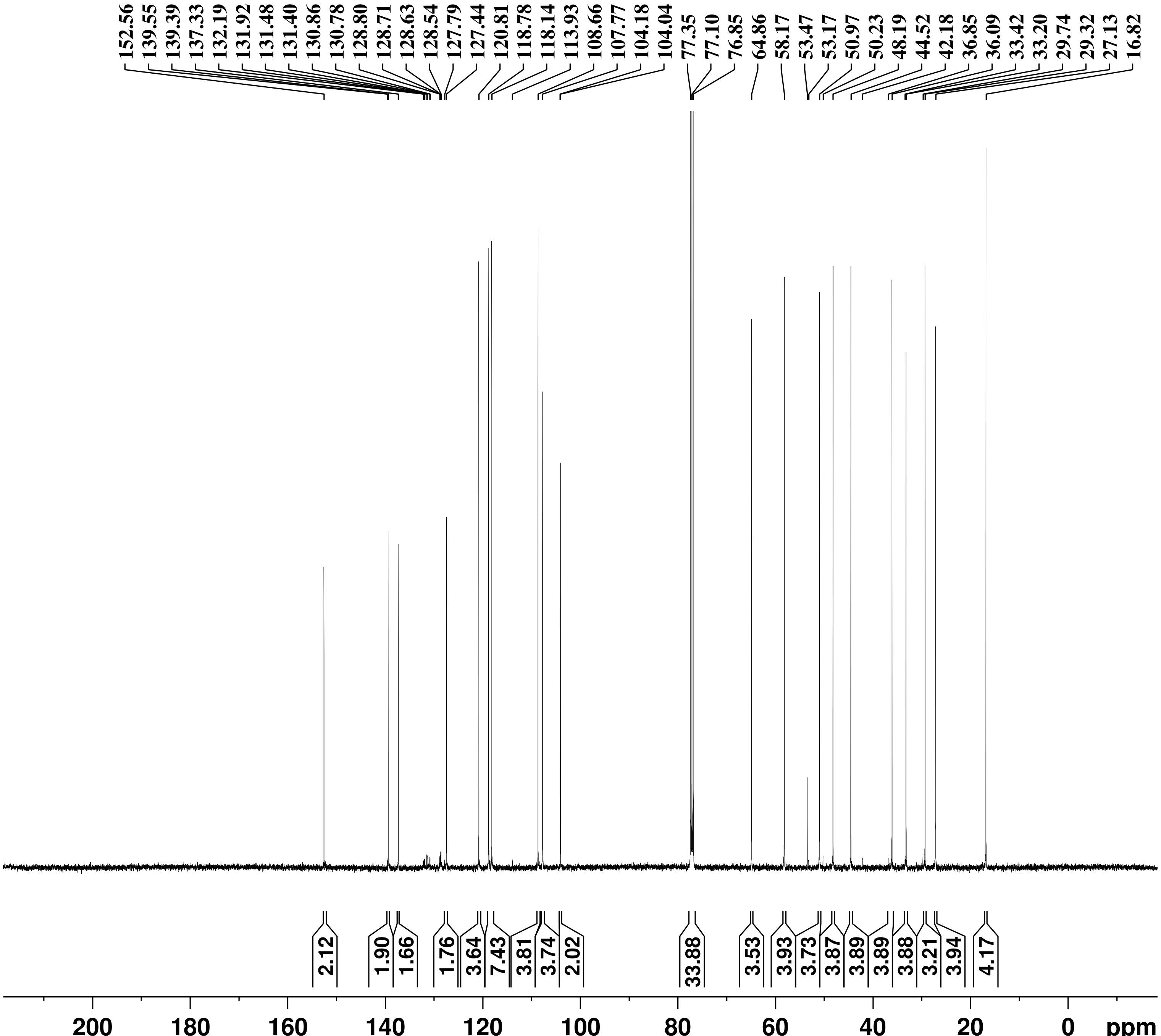

UNIVERSITY of WISCONSIN
UWMILWAUKEE

```
Current Data Parameters
NAME      KPP-II-66 (2022)
EXPNO                   2
PROCNO                  1
DATPATH  /nmr500/data/kppandey/nmr

F2 - Acquisition Parameters
Date_            20220218
Time               16.32 h
INSTRUM             spect
PROBHD   Z149001_0007 (
PULPROG           zgpg30
TD                 65536
SOLVENT            CDCl3
NS                   256
DS                     0
SWH            29761.904 Hz
FIDRES          0.908261 Hz
AQ             1.1010048 sec
RG                190.86
DW                16.800 usec
DE                 22.42 usec
TE                 298.0 K
D1            2.00000000 sec
D11           0.03000000 sec
TD0                    1
SFO1         125.7703643 MHz
NUC1                 13C
P0                  3.33 usec
P1                 10.00 usec
PLW1         64.21800232 W
SFO2         500.1320005 MHz
NUC2                  1H
CPDPRG[2         waltz16
PCPD2              80.00 usec
PLW2         14.14599991 W
PLW12         0.31829000 W
PLW13         0.16010000 W

F2 - Processing parameters
SI                 65536
SF           125.7577885 MHz
WDW                   EM
SSB                    0
LB                  1.00 Hz
GB                     0
PC                  1.40
```
