# Supplementary material for: First Total Synthesis of the Unnatural (+)-Talcarpine and (−)‑N 4‑Methyl,N 4‑21-secotalpinine
Source: ACS Omega. 2026 Apr 29;11(18):26942–56. doi: 10.1021/acsomega.5c13509 (PMC13176970; doi:10.1021/acsomega.5c13509)
Supplement: Supplementary file 2 [file ao5c13509_si_002.zip › FID for publications/26/1H NMR/pdata/1/email_KPP-II-66 (2022)_1_1.pdf]

Proton Spectrum -4-16ppm ns=16

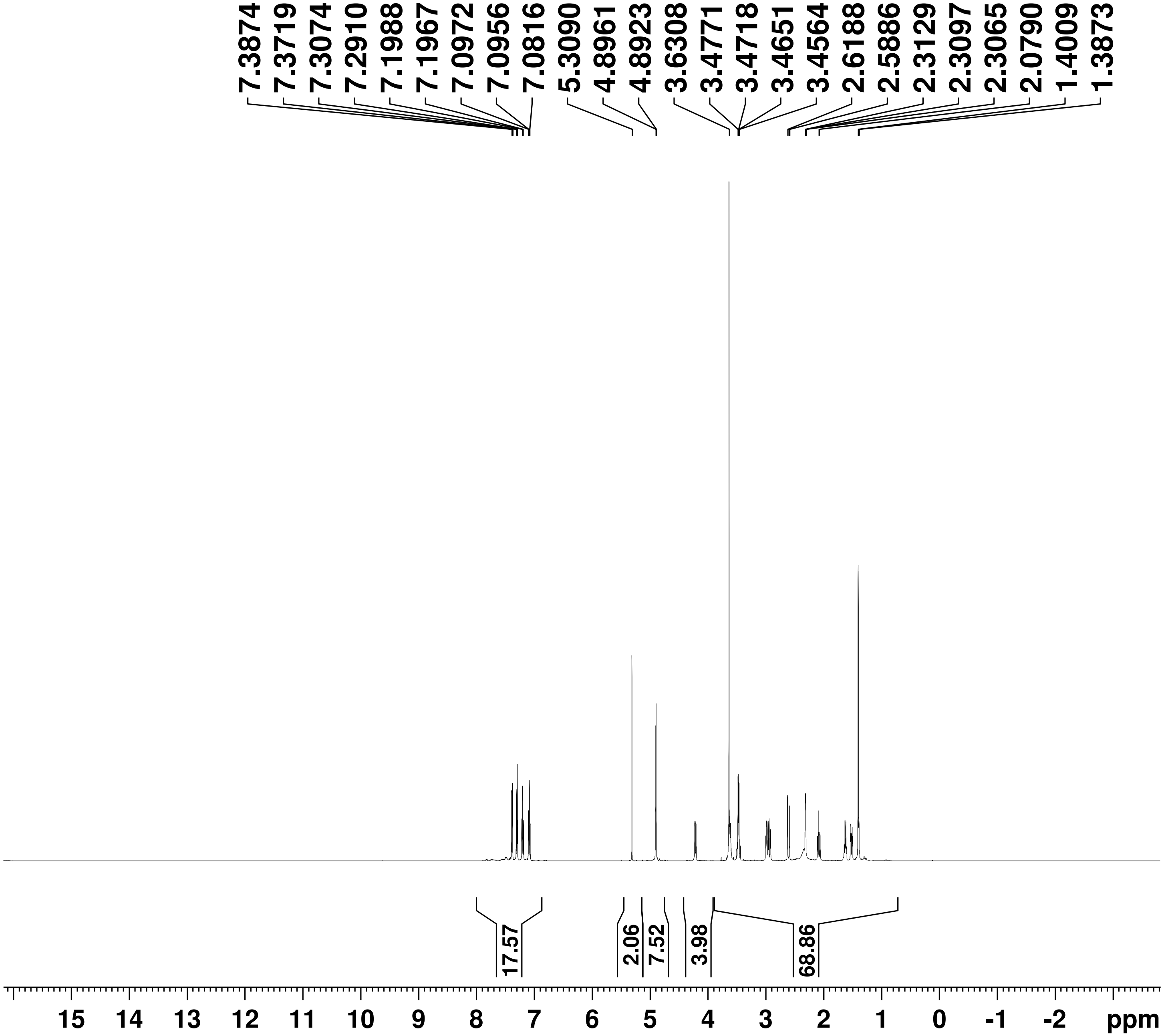

UNIVERSITY of WISCONSIN UWMILWAUKEE

```
Current Data Parameters
NAME     KPP-II-66 (2022)
EXPNO                   1
PROCNO                  1
DATPATH  /nmr500/data/kppandey/nmr

F2 - Acquisition Parameters
Date_            20220218
Time                16.16 h
INSTRUM             spect
PROBHD   Z149001_0007 (
PULPROG              zg30
TD                  65536
SOLVENT             CDCl3
NS                     16
DS                      0
SWH             10000.000 Hz
FIDRES           0.305176 Hz
AQ              3.2767999 sec
RG                  34.14
DW                 50.000 usec
DE                  25.00 usec
TE                  298.0 K
D1             1.00000000 sec
TD0                     1
SFO1          500.1330883 MHz
NUC1                   1H
P0                   4.00 usec
P1                  12.00 usec
PLW1          14.14599991 W

F2 - Processing parameters
SI                  65536
SF            500.1300000 MHz
WDW                    EM
SSB                     0
LB                   0.30 Hz
GB                      0
PC                   1.00
```
