# Supplementary material for: First Total Synthesis of the Unnatural (+)-Talcarpine and (−)‑N 4‑Methyl,N 4‑21-secotalpinine
Source: ACS Omega. 2026 Apr 29;11(18):26942–56. doi: 10.1021/acsomega.5c13509 (PMC13176970; doi:10.1021/acsomega.5c13509)
Supplement: Supplementary file 2 [file ao5c13509_si_002.zip › FID for publications/27/13C NMR (DEPT135)/pdata/1/email_KPP-II-69-2022-4.2 g sacle_3_1.pdf]

DEPT 135 Spectrum
c13dept135 CDCl3 /nmr500 kppandey 24

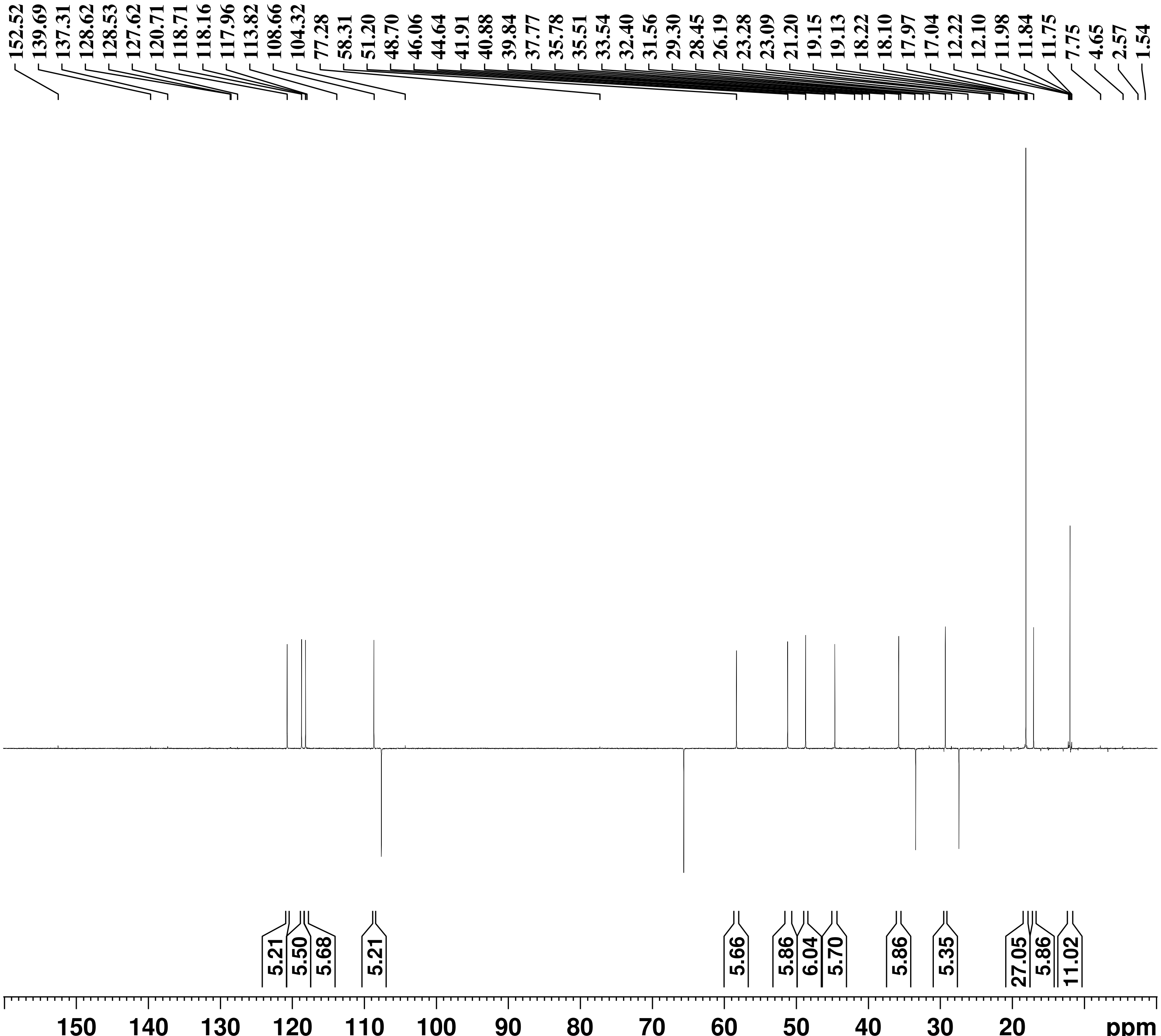

UNIVERSITY of WISCONSIN
UWMILWAUKEE

```
Current Data Parameters
NAME      KPP-II-69-2022-4.2 g sacle
EXPNO                    3
PROCNO                   1
DATPATH   /nmr500/data/kppandey/nmr

F2 - Acquisition Parameters
Date_           20220329
Time               11.02 h
INSTRUM            spect
PROBHD    Z149001_0007 (
PULPROG        deptsp135
TD                 65536
SOLVENT            CDCl3
NS                   128
DS                     8
SWH            20161.291 Hz
FIDRES          0.615274 Hz
AQ             1.6252928 sec
RG                190.86
DW                24.800 usec
DE                 23.06 usec
TE                 298.0 K
CNST2        145.0000000
D1            2.00000000 sec
D2            0.00344828 sec
D12           0.00002000 sec
TD0                    1
SFO1         125.7678491 MHz
NUC1                 13C
P1                 10.00 usec
P13              2000.00 usec
PLW0                   0 W
PLW1         64.21800232 W
SPNAM[5]     Crp60comp.4
SPOAL5             0.500
SPOFFS5                0 Hz
SPW5          9.81169987 W
SFO2         500.1320005 MHz
NUC2                  1H
CPDPRG[2         waltz16
P3                 12.00 usec
P4                 24.00 usec
PCPD2              80.00 usec
PLW2         14.14599991 W
PLW12         0.31829000 W

F2 - Processing parameters
SI                 32768
SF           125.7577885 MHz
WDW                   EM
SSB                    0
LB                  1.00 Hz
GB                     0
PC                  1.40
```
