# Supplementary material for: First Total Synthesis of the Unnatural (+)-Talcarpine and (−)‑N 4‑Methyl,N 4‑21-secotalpinine
Source: ACS Omega. 2026 Apr 29;11(18):26942–56. doi: 10.1021/acsomega.5c13509 (PMC13176970; doi:10.1021/acsomega.5c13509)
Supplement: Supplementary file 2 [file ao5c13509_si_002.zip › FID for publications/27/1H NMR/pdata/1/email_KPP-II-69-2022-4.2 g sacle_1_1.pdf]

Proton Spectrum -4-16ppm ns=16

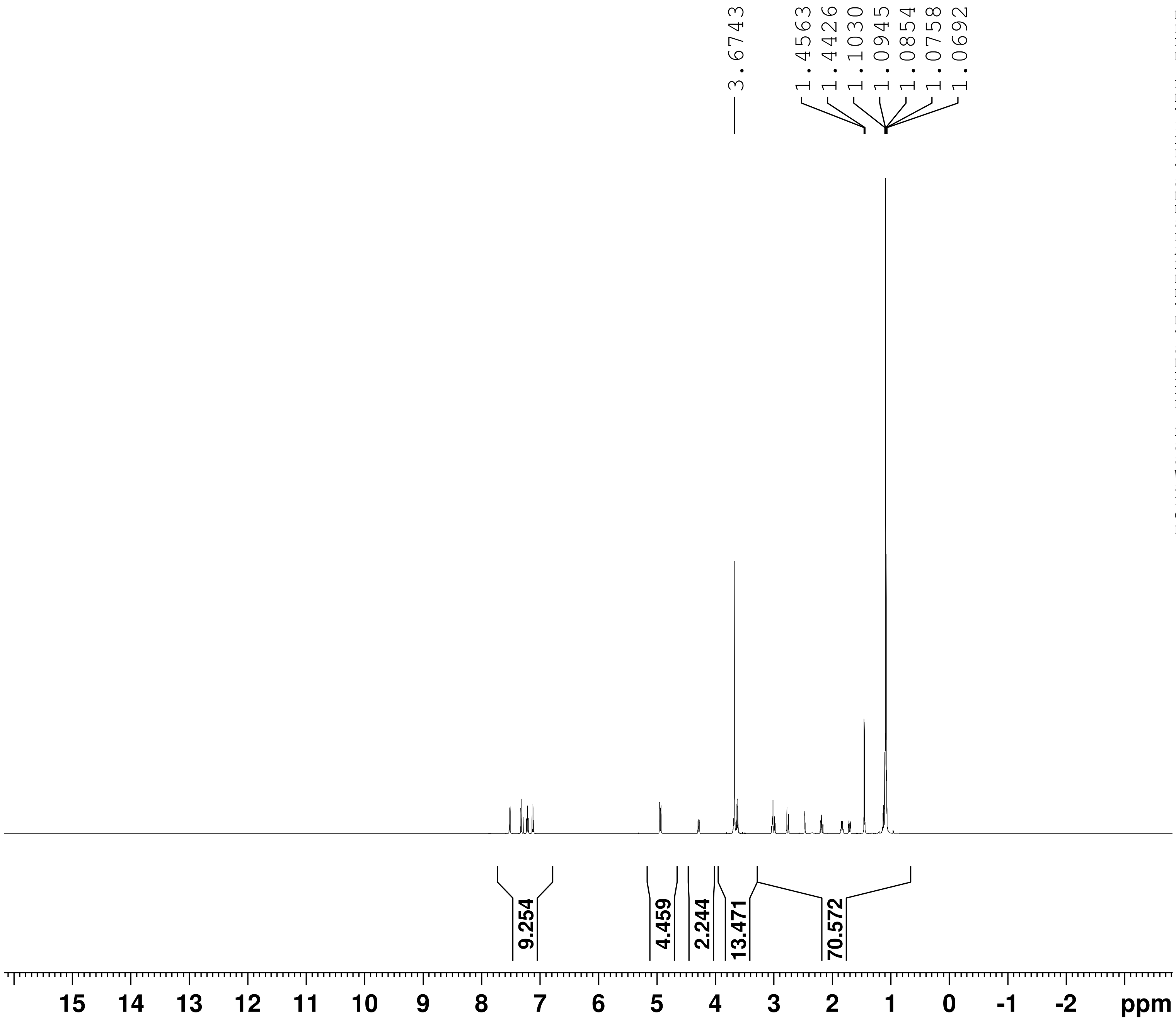


```
NAME       KPP-II-69-2022-4.2 g sacle
EXPNO                    1
PROCNO                   1
DATPATH   /nmr500/data/kppandey/nmr

F2 - Acquisition Parameters
Date_          20220329
Time              10.36 h
INSTRUM           spect
PROBHD   Z149001_0007 (
PULPROG            zg30
TD                65536
SOLVENT           CDCl3
NS                   16
DS                    0
SWH           10000.000 Hz
FIDRES         0.305176 Hz
AQ            3.2767999 sec
RG                27.15
DW               50.000 usec
DE                25.00 usec
TE                298.0 K
D1           1.00000000 sec
TD0                   1
SFO1        500.1330883 MHz
NUC1                 1H
P0                 4.00 usec
P1                12.00 usec
PLW1        14.14599991 W

F2 - Processing parameters
SI                65536
SF          500.1300000 MHz
WDW                  EM
SSB                   0
LB                 0.30 Hz
GB                    0
PC                 1.00
```
